# Multiomics Characterization of the Canada Goose Fecal Microbiome Reveals Selective Efficacy of Simulated Metagenomes

**DOI:** 10.1128/spectrum.02384-22

**Published:** 2022-11-01

**Authors:** Joshua C. Gil, Sarah M. Hird

**Affiliations:** a Department of Molecular and Cell Biology, University of Connecticut, Storrs, Connecticut, USA; b Institute for Systems Genomics, University of Connecticut, Storrs, Connecticut, USA; University of Nevada Reno

**Keywords:** Canada goose, avian, metagenome, metatranscriptome, microbiome

## Abstract

16S rRNA amplicon sequences are predominantly used to identify the taxonomic composition of a microbiome, but they can also be used to generate simulated metagenomes to circumvent costly empirical shotgun sequencing. The effectiveness of using “simulated metagenomes” (shotgun metagenomes simulated from 16S rRNA amplicons using a database of full genomes closely related to the amplicons) in nonmodel systems is poorly known. We sought to determine the accuracy of simulated metagenomes in a nonmodel organism, the Canada goose (Branta canadensis), by comparing metagenomes and metatranscriptomes to simulated metagenomes derived from 16S amplicon sequencing. We found significant differences between the metagenomes, metatranscriptomes, and simulated metagenomes when comparing enzymes, KEGG orthologies (KO), and metabolic pathways. The simulated metagenomes accurately identified the majority (>70%) of the total enzymes, KOs, and pathways. The simulated metagenomes accurately identified the majority of the short-chain fatty acid metabolic pathways crucial to folivores. When narrowed in scope to specific genes of interest, the simulated metagenomes overestimated the number of antimicrobial resistance genes and underestimated the number of genes related to the breakdown of plant matter. Our results suggest that simulated metagenomes should not be used in lieu of empirical sequencing when studying the functional potential of a nonmodel organism’s microbiome. Regarding the function of the Canada goose microbiome, we found unexpected amounts of fermentation pathways, and we found that a few taxa are responsible for large portions of the functional potential of the microbiome.

**IMPORTANCE** The taxonomic composition of a microbiome is predominately identified using amplicon sequencing of 16S rRNA genes, but as a single marker, it cannot identify functions (genes). Metagenome and metatranscriptome sequencing can determine microbiome function but can be cost prohibitive. Therefore, computational methods have been developed to generate simulated metagenomes derived from 16S rRNA sequences and databases of full-length genomes. Simulated metagenomes can be an effective alternative to empirical sequencing, but accuracy depends on the genomic database used and whether the database contains organisms closely related to the 16S sequences. These tools are effective in well-studied systems, but the accuracy of these predictions in a nonmodel system is less known. Using a nonmodel bird species, we characterized the function of the microbiome and compared the accuracy of 16S-derived simulated metagenomes to sequenced metagenomes. We found that the simulated metagenomes reflect most but not all functions of empirical metagenome sequencing.

## INTRODUCTION

All vertebrates host microorganisms in and on their bodies. These “host-associated microbiomes” have strong effects on host biology that range from beneficial to detrimental (1, 2). On the beneficial end of the spectrum, the microbiota can help modulate the host’s immune system, break down indigestible nutrients (e.g., cellulose/hemicellulose) or harmful substances (e.g., xenobiotics), regulate hormones, exclude pathogens, and synthesize vital nutrients (3–8). On the detrimental end of the spectrum, a “dysbiotic” microbiome can harm the host by allowing pathogenic bacteria to colonize the gut or by stimulating excessive inflammation, potentially leading to gastrointestinal disorders and an overall decline in health (9). Understanding the composition and diversity of the microbiome is required to elucidate the role of the microbiome in host health, fitness, and evolution. The microbiome is dynamic and influenced by many intrinsic (from the host) and extrinsic factors (from the host’s environment) (5). Intrinsic influences include host anatomy, morphology, sex, age, reproduction strategy, and immune system; extrinsic influences can include host habitat, the type of food available, altitude, season, and environmental contamination (5, 10–15).

Numerous microbiome studies use a single marker, the 16S rRNA gene (here 16S), to taxonomically identify the microbiota. 16S sequencing is an effective method to identify what microbes constitute the microbiota (although the taxonomic level of an amplicon that can be confidently assigned varies and rarely includes species or strain), but 16S data are unable to describe the functions (i.e., genes) of the microbiome (16). Taxonomic composition and functional repertoire are two complementary aspects of the microbiome; they are not independent, but they are also not entirely overlapping. Two microbes may have identical 16S sequences, but they can have significant differences in their metabolic potential (17–19). Conversely, microbes can have identical copies of a gene and belong to distally related taxa; this results in microbes or communities of microbes that are more functionally similar than they are taxonomically similar (20–22). For a microbiome, having a specific taxon can be less important than having a specific metabolic function performed (20, 23, 24). Thus, the microbiome can perform metabolic functions with taxonomically distinct microbiomes. This functional redundancy provides stability for the community in the event that one or more organisms are removed (20, 23).

To characterize the functional potential of these communities, the total genomic DNA from the microbiome can be extracted and sequenced. The total genomic DNA is referred to as the metagenome (MG), and it contains the functional potential of the microbiome (16, 25); it is “potential” because it describes every gene present, not the genes currently being used. Similarly, the total RNA, or metatranscriptome (MT), present in a microbiome sample can be extracted, converted to cDNA, and sequenced. The metatranscriptome contains the genes and pathways that are actively being transcribed (2, 16).

Metagenomes and metatranscriptomes provide functional as well as taxonomic insight into the microbial community but are more expensive than 16S sequencing (16). Shotgun-omics sequencing is more costly because sampling requires extra preparation for DNA or RNA preservation; presequence processing, including ribodepletion of rRNA, is often required to enrich for mRNA, and higher sequencing depth is required to assemble and annotate the genes. 16S sequencing is significantly less expensive than shotgun-omics approaches because it is a single marker accessible with a single PCR. However, 16S sequencing does not contain information about the functional characteristics of the microbial community (16). Computational methods have been developed with the intention to circumvent the need for metagenomic or metatranscriptomic sequencing; metagenome simulation software uses publicly available genomic resources to create a “simulated metagenome” (simulatedMG) from empirical 16S sequence data. One such piece of software is PICRUSt2 (26), which uses data from 16S rRNA sequencing, identifies closely related organisms in a database of complete genomes, and simulates the metagenome (including what genes may be present and the quantity of the each gene) based on how many amplicons from a particular organism are present in the 16S data. This form of metagenome simulation can be largely accurate for samples that originate from well-studied organisms (e.g., human microbiome and model organisms); however, because PICRUSt2 is dependent on genomic databases, it can provide weaker predictions for nonmodel systems (26–28). Simulating metagenomes from diverse systems could greatly increase our understanding of the functional potential of such communities; but to do so with confidence, it is essential to understand where and how such methods work well and where and how they do not.

Herein, we collected 16S data and shotgun metagenomes (MGs) and metatranscriptomes (MTs) to compare to simulated metagenomes (simulatedMGs) using a wild, nonmodel bird species, the Canada goose (Branta canadensis). Canada geese are a ubiquitous species across North America and are one of the few folivores in the avian clade. They thrive in many urban and periurban environments (29, 30) and are often a public concern because waterfowl can pose a substantial risk to human health as a vector for avian flu, coliform contamination, and antimicrobial resistance genes (AMRs) (31–39). Canada geese are not currently known to be significant vectors for zoonotic flus, but they are carriers for many opportunistic pathogens and AMRs despite having generally fewer microbes in their feces and a less diverse microbiome than many other bird species (32–34, 36, 40–42). By studying Canada geese microbiomes, we can obtain a better understanding of the potential risk of pathogens and AMRs as well as elucidate the function of the microbiome in an herbivorous bird species.

Specifically, we collected 16S, MG, and MT data on fecal samples from nine Canada geese and identified the taxa, pathways, and functions present. We also used the 16S data to simulate metagenomes using PICRUSt2 and compared them to the MG and MT. The goals of this research were to

(i) Categorize the Canada goose fecal microbiota by identifying what microbes are present.
(ii) Determine the accuracy of the simulated metagenomes by comparing the diversity and number of shared enzymes (enzyme commission numbers [ECs]), KEGG orthologies (KOs), and metabolic pathways in each method.
(iii) Identify genes or pathways that may be functionally important based on their abundance in each method.
(iv) Look for antimicrobial resistance genes (AMRs) found in Canada geese and see if they are accurately identified by PICRUSt2.
(v) Determine the relative contributions of constituent microbes on the functional potential of the microbiome.

## RESULTS

### Shotgun metaomics data, sequencing, and simulation.

**FIG 1:**
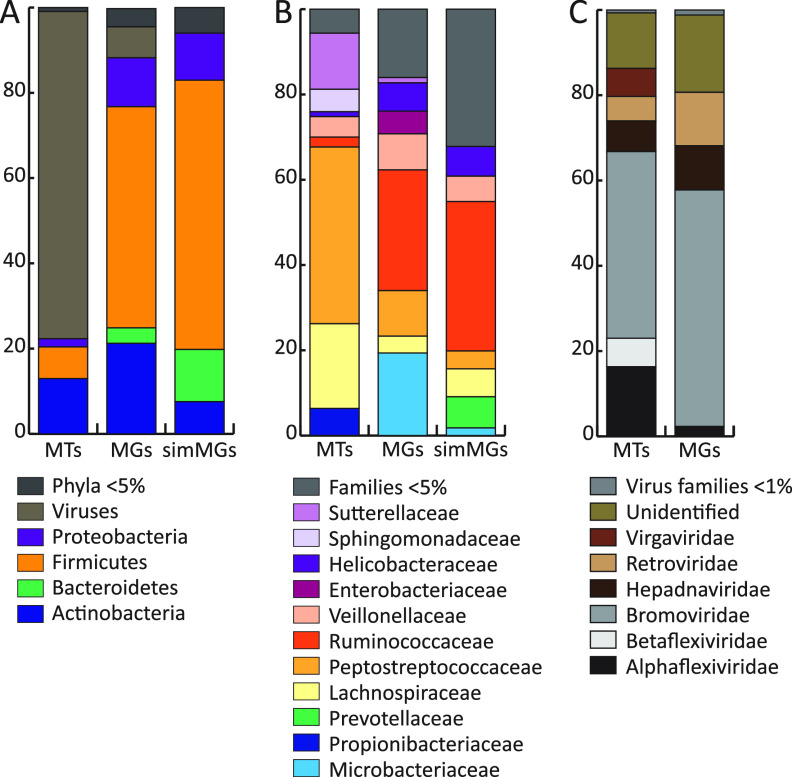
Average taxonomic relative abundance of the different data types: metatranscriptomes (MTs), metagenomes (MGs), and simulated metagenomes (simMGs). (A) Relative abundance bar chart of the averaged phylum with viruses included. Phyla less than 5% of the relative abundance grouped together. (B) Averaged bacterial family relative abundance of the different data types; families less than 5% grouped together. (C) Averaged viral family relative abundance found in the MGs and MTs; viruses less than 1% relative abundance grouped together.

The nine metagenomes (MGs) and nine metatranscriptomes (MTs) were cleaned and trimmed, yielding an average of 22,409,670 reads per MG and 21,116,359 reads per MT. After HUMANn2’s organism-agnostic translated search, 40.78% (*x̄* = 40.78%) of the reads in the metagenomes and 30.69% (*x̄* = 30.69) of the reads in the metatranscriptomes mapped to a known protein sequence. In the MGs and MTs, we identified 1,607,386 unique UniProt IDs; after running the datamining script, we identified 583,903 UniProt IDs that were not associated with prokaryotes. We wrote a datamining script and extracted taxonomic data associated with every UniProt ID, and those that did not contain either “Bacteria” or “Archaea” were removed. Large portions of the MGs and MTs were viruses (Fig. 1A), and the majority of the removed UniProt IDs were associated with viruses. See the Methods for detailed information about the datamining script we used. PICRUSt2 was used to simulate a third metagenomic data set based on the 16S rRNA data from the same samples. PICRUSt2 output 1,129 amplicon sequence variants (ASVs) with a nearest-sequenced taxon index (NSTI) assigned to each sequence. Of these, 71 ASVs (6.2%) were assigned an NSTI greater than 2 and were discarded as too divergent from any sequenced genome. Those 71 ASVs were 29.49% of the total sequences.

### Taxonomic composition.

We characterized the samples at the phylum and (Fig. 1A) family (Fig. 1B) levels for both broad- and fine-scale taxonomic resolution of the microbiota. Phylum-level comparisons showed notable differences in the averaged taxonomic composition of the MG, MT, and simulatedMG samples (Fig. 1A). The predominate phyla detected in all three methods included *Actinobacteria*, *Proteobacteria*, and *Firmicutes*. The simulatedMGs identified more phyla than the MGs and the MTs. We found *Bacteroidetes* in the MGs and simulatedMGs but not in the MTs. Large portions of the MGs and MTs were viruses. See Table S1 in the supplemental material for a complete list of all phyla detected.

A few virus families were detected in both the MGs and MTs (Fig. 1C). The MGs had six virus families present, the most abundant being *Bromoviridae*, which comprised an average of 55.47% of the detected viruses per sample, followed by *Retroviridae* with 12.52% per sample. Approximately 18.13% of the viruses could not be classified into a family in the MGs. The MTs had more total viruses with 10 virus families present. Similar to the MGs, *Bromoviridae* was the most abundant virus family, with 43.77% *Bromoviridae* per sample on average. Similar to the MGs, the unclassified viruses comprised 13.02% of the averaged relative abundance. See Table S2 for a complete list of all virus families detected.

### KO, EC, and pathway count analysis: full data sets.

To characterize the functions of the microbiota, we used KOs (groups of orthologous genes grouped by molecular function) (43, 44), ECs (enzymes manually linked to experimental data) (45), and metabolic pathways (MetaCyc pathways) identified using the MetaCyc database (46, 47). A total of 12,257 KOs, 3,296 ECs, and 633 pathways were identified in the full data set. HUMAnN2 analysis of the MGs yielded 9,783 KOs (*x̄* = 5,366), 2,884 ECs (*x̄* = 1,961), and 588 pathways (*x̄* = 444). The MTs yielded 7,778 KOs (*x̄* = 3,302), 2,089 ECs (*x̄* = 1,159), and 436 pathways (*x̄* = 275). PICRUSt2 analysis (simulatedMGs) of the complementary 16S sequences from the ZymoBionics extractions identified 6,451 KOs (*x̄* = 5,176), 2,021 unique ECs (*x̄* = 1,688), and 388 pathways (*x̄* = 338).

**FIG 2:**
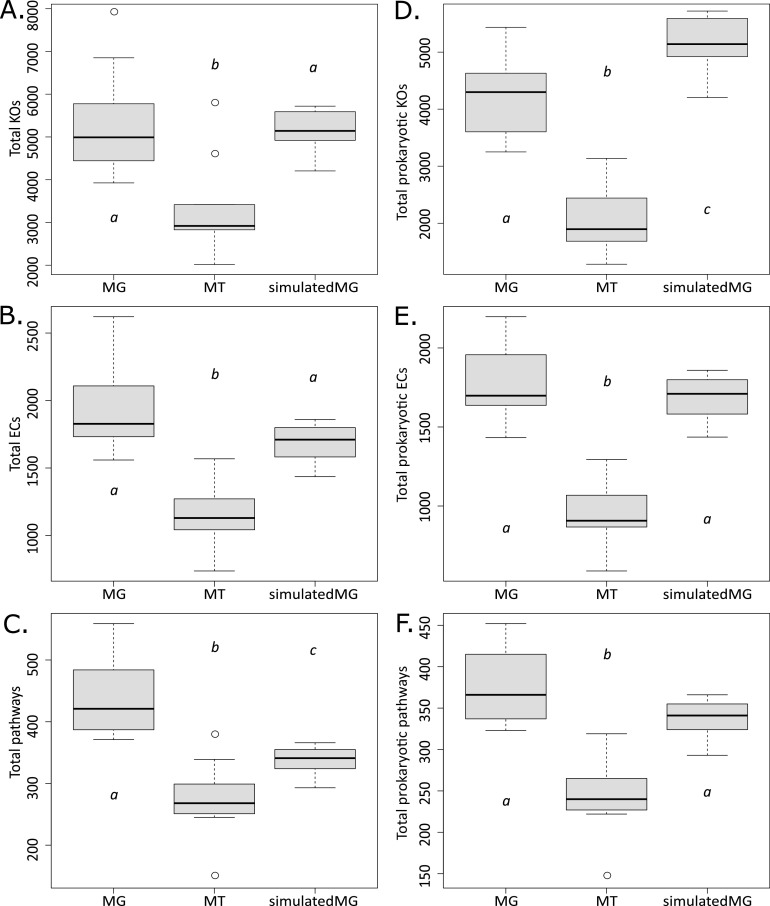
Box plot of the number of identified KOs, ECs, and pathways identified in the MGs, MTs, and simulatedMGs. (A) Total KOs. (B) Total ECs. (C) Total number of pathways detected. (D to F) Number of prokaryotic KOs (D), ECs (E), and pathways (F) identified, respectively. Significance was determined by Wilcoxon rank-sum *t* test at *P* < 0.05, and significance between groups is denoted by “a,” “b,” or “c” on the box plot.

Significant differences in the raw number of KOs, ECs, and pathways found in each method were calculated using the Wilcoxon rank-sum *t* test (Fig. 2A to C). We found no significant difference in the number of KOs between the simulatedMGs data and MGs (simulatedMGs:MGs, *P* = 0.6511). There were significant differences in KOs between the simulatedMGs and MTs as well as the MGs and MTs (simulatedMGs:MTs, *P* = 0.012; MGs:MTs, *P* = 0.012). We detected no significant difference between the number of ECs identified by the simulatedMGs and MGs (simulatedMGs:MGs, *P* = 0.11349). There were significant differences between the comparisons of simulatedMGs:MTs and MGs:MTs (*P* = 0.00058 and *P* = 0.00025, respectively). There were significant differences in the number of pathways identified in all three methods (simulatedMGs:MGs, *P* = 0.00012; simulatedMGs:MTs, *P* = 0.01922; MGs:MTs, *P* = 0.00033).

### KO, EC, and pathway count analysis: prokaryote-only data sets.

Because the simulation software only simulates bacteria and archaea, we removed the viral and eukaryotic KOs, ECs, and pathways for a more “fair” comparison. This yielded 6,089 KOs (*x̄* = 4,338), 2,409 ECs (*x̄* = 1,813), and 454 pathways (*x̄* = 358) for the MGs. The MTs yielded 3,929 KOs (*x̄* = 2,112), 1,539 ECs (*x̄* = 954), and 318 pathways (*x̄* = 217).

We tested the KOs, ECs, and pathways that belong only to prokaryotes again using the Wilcoxon rank-sum *t* test (Fig. 2D to F). We found significant differences between the number of KOs between the simulatedMGs data and MGs (simulatedMGs:MGs, *P* = 0.01061). There were significant differences in KOs between the simulatedMGs and MTs as well as the MGs and MTs (simulatedMGs:MTs, *P* = 0.012; MGs:MTs, *P* = 0.012). We detected no significant difference between the number of ECs identified by the simulatedMGs and MGs (simulatedMGs:MGs, *P* = 0.6647). There were significant differences between the comparisons of simulatedMGs:MTs and MGs:MTs (*P* = 0.00012 and *P* = 0.00012, respectively). We found that significant differences were still observed between simulatedMGs:MTs (*P* = 0.0000082) and MGs:MTs (*P* = 0.00012); however, there was no longer a significant difference in the number of pathways identified between the simulatedMGs and MGs (*P* = 0.81012).

### Beta diversity of simulated metagenomes, sequenced metagenomes, and metatranscriptomes.

**FIG 3:**
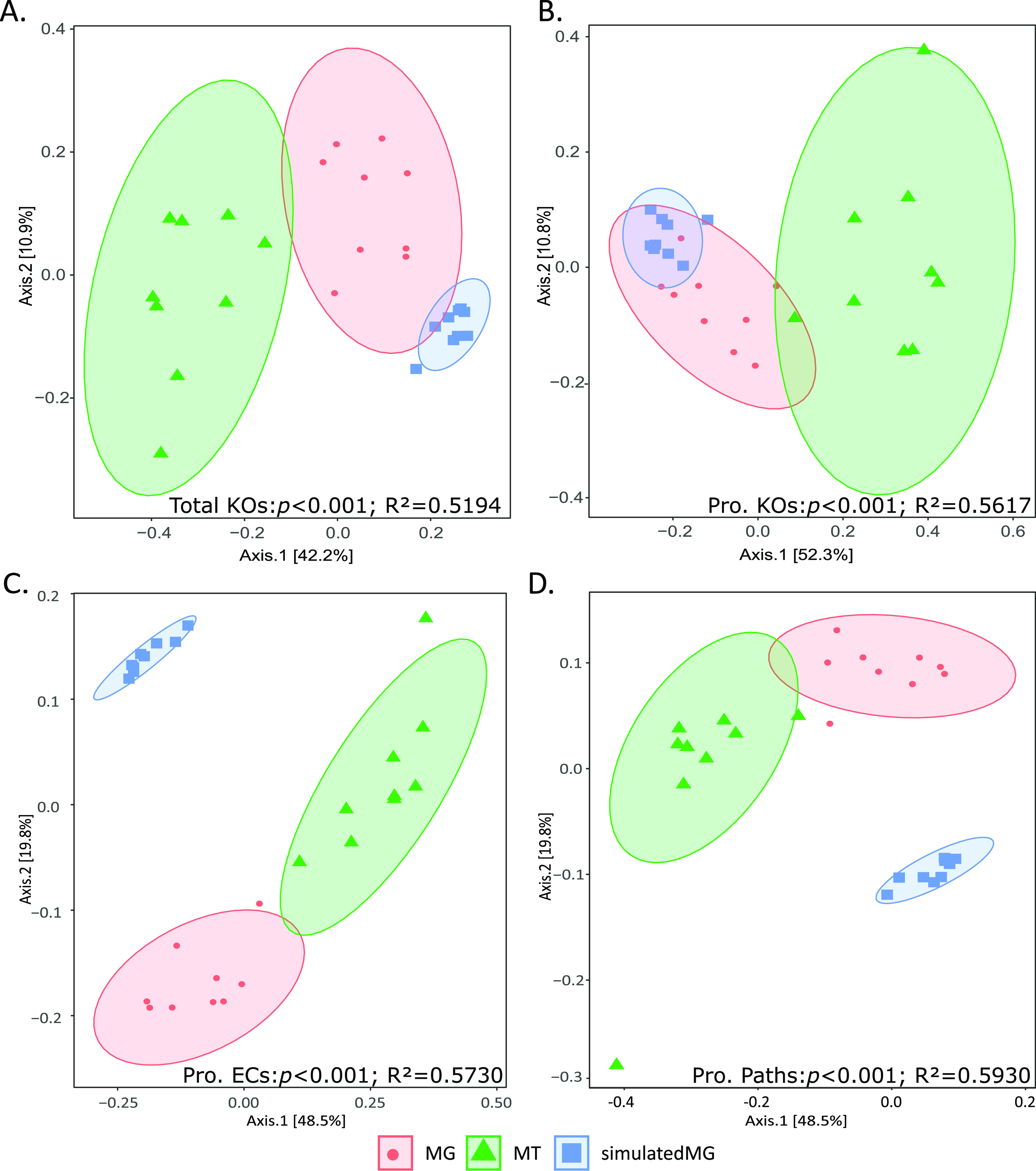
PCoA ordinations of the Jaccard similarity for the ECs, KOs, and pathways for each of the three data types: MGs, MTs, and simulatedMGs. (A) Total KOs. (B) Prokaryote-only KOs. (C) Prokaryote-only ECs. (D) Prokaryotic metabolic pathways. Each dot represents one sample, red circles are the MGs, green triangles are the MTs, and blue squares are the simulatedMGs. PERMANOVA shows significant differences (*P* < 0.001) between the different methods for the KOs, ECs, and pathways in both the total data set and prokaryotic-only data set; 95% confidence intervals are placed around each of the three groups.

To see how the samples compared to one another, principal coordinate analysis (PCoA) ordinations were generated to compare the three data types. Visual analysis of the PCoA plots showed that the simulatedMGs, MGs, and MTs cluster separately for KOs (Fig. 3A), ECs, and pathways (Fig. S1). Using a permutational multivariate analysis of variance (PERMANOVA), there was a significant difference between the simulatedMGs, MGs, and MTs for KOs, ECs, and pathways, and greater than 50% of the variation was explained by data type (KOs, *P* < 0.001, *R*^2^ = 0.5194; ECs, *P* < 0.001, *R*^2^ = 0.51433; pathways, *P* < 0.001, *R*^2^ = 0.60249).

When viral and eukaryotic KOs, ECs, and pathways were removed, the simulatedMGs, MGs, and MTs clustered separately for the KOs, ECs, and pathways (Fig. 3B to D). Using a PERMANOVA, there was a significant difference between the simulatedMGs, MGs, and MTs for KOs, ECs, and pathways (KOs, *P* < 0.001, *R*^2^ = 0.56167; ECs, *P* < 0.001, *R*^2^ = 0.5370; pathways, *P* < 0.001, *R*^2^ = 0.59299).

### Comparative analyses.

**FIG 4:**
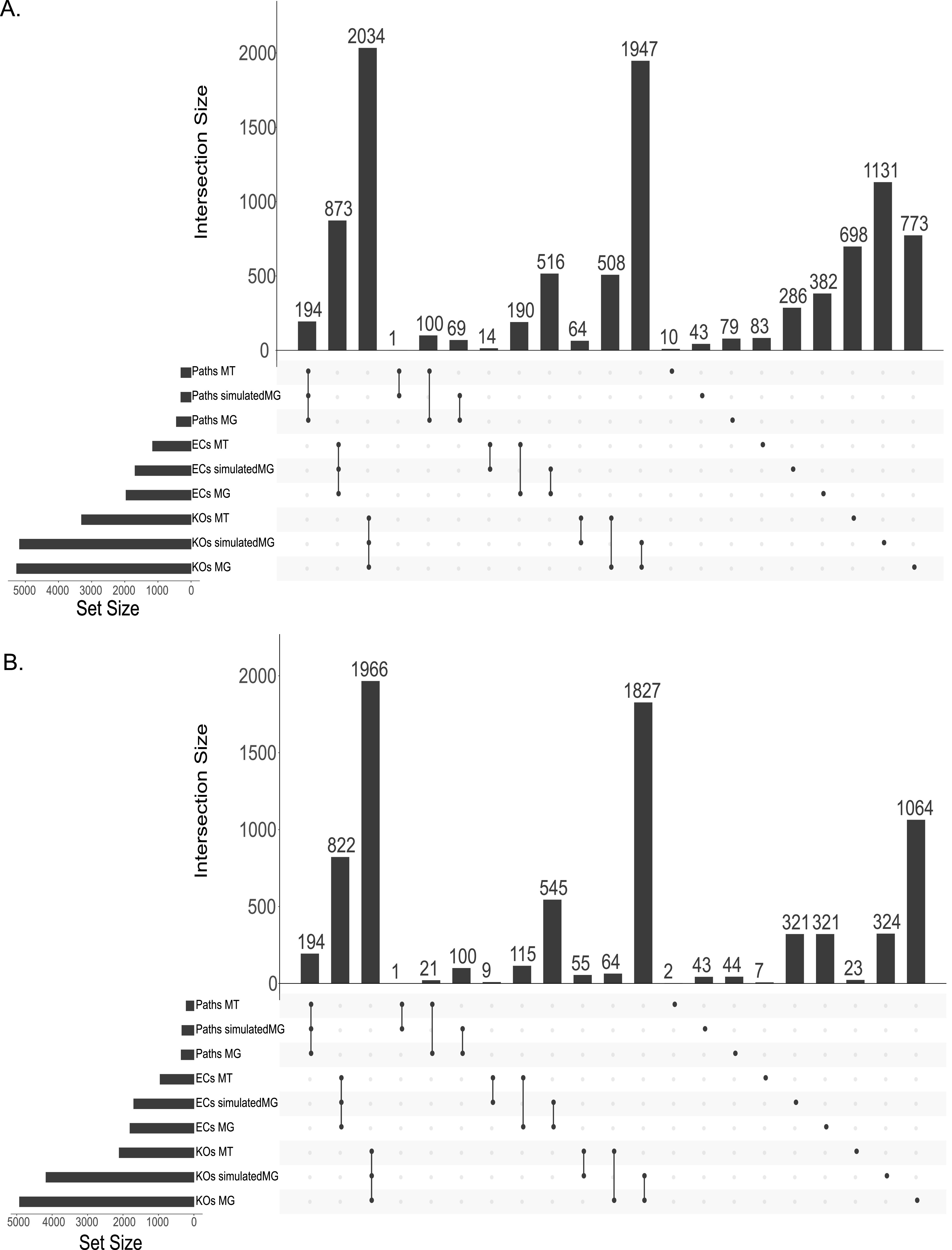
UpSet plot for averaged shared KEGG orthologies (KOs), enzyme commission numbers (ECs), and pathways (Paths) for all nine samples. UpSet plots are an alternative to complex Venn diagrams. (A) Total data set showing the KOs, ECs, and pathways that were found in only the MGs, MTs, and simulatedMGs or found in some combination of the three. (B) Prokaryote-only data showing the KOs, ECs, and pathways found in only the MGs, MTs, and simulatedMGs or found in some combination of the three. The black dot (•) indicates that the KOs, ECs, and pathways were only the MGs, MTs, or simulatedMGs. Two black dots connected by a black line (•-•) indicate that it was shared by two (MGs:MTs, MGs:simulatedMGs, or MTs:simulatedMGs). Three black dots connected by a black line (•-•-•) indicate that the KOs, ECs, and pathways were shared by all three (MGs:MTs:simualtedMGs).

To identify how many of the KOs, ECs, and pathways were shared versus unique in the total data set and in the prokaryote-only data, we used UpSet plots (Fig. 4). In the total data set, we found that the largest portions of KOs, ECs, and pathways were found in the intersection of all three data types followed by the intersection of only simulatedMGs and MGs. The intersection of the simulatedMGs and the MTs consistently had the fewest KOs, ECs, and pathways.

We calculated the percent similarity of the identified KOs, ECs, and pathways (Table 1). The greatest percent similarity of the pairwise comparisons was the simulatedMGs and the MGs for the KOs, ECs, and pathways, followed by MGs and MTs and simulatedMGs and MTs (KOs, *x̄* = 49.05%; ECs, *x̄* = 61.94%; pathways, *x̄* = 63.11%). The average percent similarity of the KOs, ECs, and pathways was lowest between all three methods.

**TABLE 1:** Averaged percent similarity of shared KOs, ECs, and pathways between the different data types

Dataset comparison	Total dataset (nonprokaryotes included)^*a*^			Prokaryotes only^*a*^		
	KOs, $\bar{X}$ similarity	ECs, $\bar{X}$similarity	Pathway, $\bar{X}$similarity	KOs, $\bar{X}$similarity	ECs, $\bar{X}$similarity	Pathway, $\bar{X}$similarity
simulatedMGs:MGs	76.21%	76.14%	75.29%	81.05%	78.03%	84.27%
simulatedMGs:MTs	49.05%	61.94%	63.15%	55.68%	62.54%	69.51%
MGs:MTs	59.46%	68.13%	73.08%	65.50%	67.73%	74.16%
simulatedMGs:MGs:MTs	44.50%	54.57%	55.12%	50.94%	55.42%	63.46%

a Total data set, including the prokaryotes, eukaryotes, and viral data, compared with only prokaryotic KOs, ECs, and pathways present. Means are rounded to the nearest whole number.

When the nonprokaryotic KOs, ECs, and pathways were removed, the intersection of all three data types still contained the largest amount of KOs, ECs, and pathways (Fig. 4), and second largest was the intersection of the MGs and simulatedMGs. The greatest percent similarity of the pairwise comparisons was the simulatedMGs and the MGs for KOs, ECs, and pathways, followed by MGs and MTs and then simulatedMGs and MTs. The lowest percent similarity for the KOs, ECs, and pathways was in the intersection of the MGs, MTs, and simulatedMGs (Table 1).

We wanted to quantify the effect of the nonprokaryotic data on the averaged percent similarity between the three data types and calculated the change in percent similarity in KOs, ECs, and pathways. The percent similarity of the KOs increased across each pair (MGs:simulatedMGs = Δ+4.84%; MGs:MTs = Δ+6.04%; simulatedMGs:MTs = Δ+6.63). The percent similarity for ECs in MGs:simulatedMGs and simulatedMGs:MTs increased (Δ+1.89 and Δ+0.60%, respectively); however, MGs:MTs decreased (Δ−0.40%). The percent similarity of the pathways in simulatedMGs:MGs had the largest change (Δ+8.98%), followed by simulatedMGs:MTs (Δ+6.36%) and MGs:MTs (Δ+1.08%). The similarity between all three data types increased in the KOs, ECs, and pathways (KOs = Δ+6.44%; ECs = Δ+0.85%; pathways = Δ+8.34%).

### Functionally relevant pathways and enzyme analysis.

**FIG 5:**
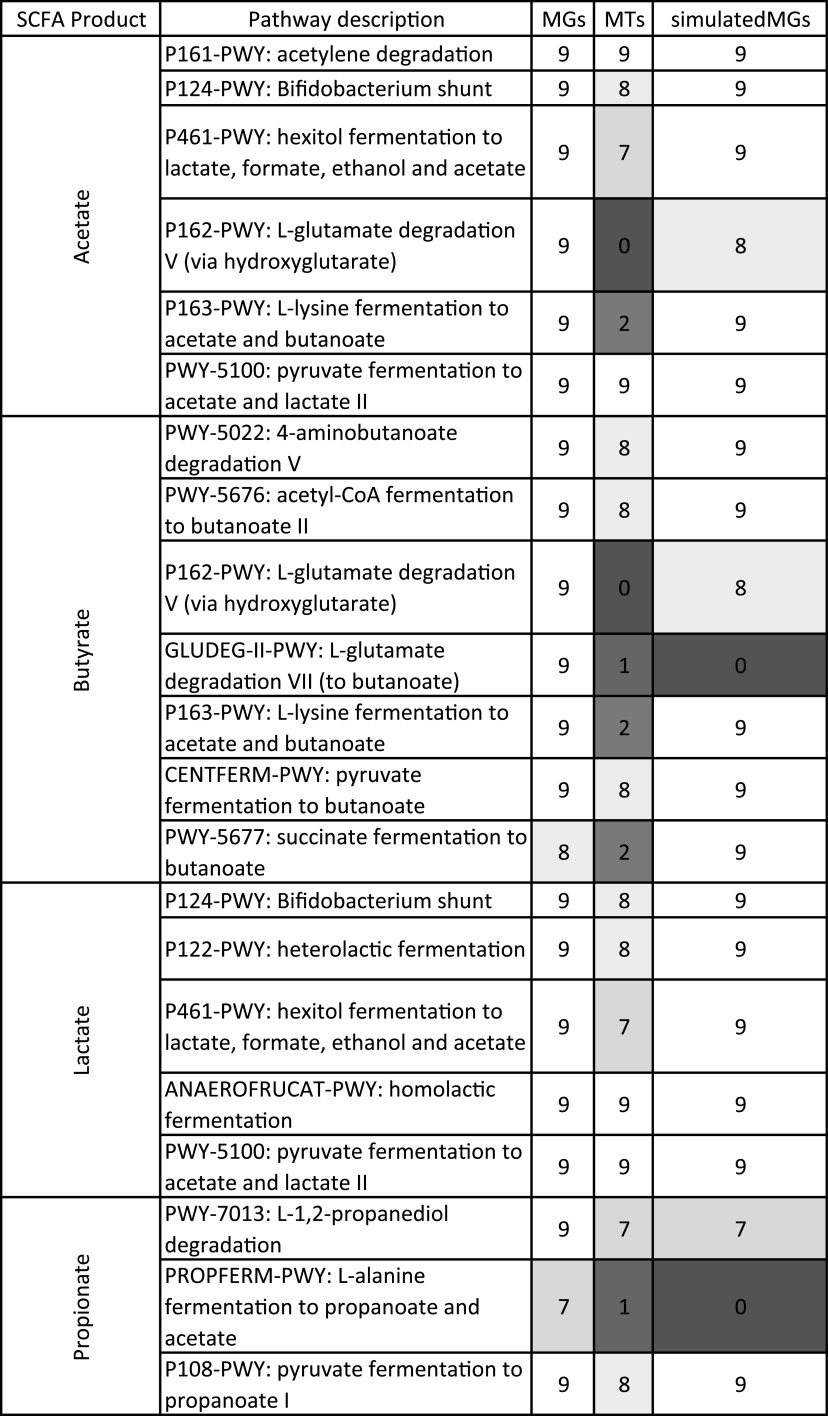
Heat map of SCFA pathways that were found in the different data types. No complete SCFA pathways found in any sample are dark gray. Pathways found in all nine samples in each group are white.

Short-chain fatty acids (SCFAs) are the byproduct of microbial-dependent fermentation of indigestible dietary fibers like cellulose and hemicellulose (3, 48). In herbivores, the main SCFA pathways of interest produce butyrate, acetate, propionate, and lactate (3). The prevalence of these different SCFA pathways varies depending on the organism, and so we were interested in what SCFA pathways were identified depending on the method used (Fig. 5). Six pathways produce acetate, seven produce butyrate, five produce lactate, and three produce propionate. Five of the SCFA pathways produce multiple SCFAs; P162-PWY and P163-PWY produce both acetate and butyrate. Pathways P124-PWY, P461-PWY, and PWY-5100 produce acetate and lactate. All 16 pathways were identified in every metagenome sample with two exceptions; one metagenome did not have the butyrate pathway PWY-5677 (succinate fermentation to butanoate), and two did not have the propionate pathway PROPFERM-PWY (l-alanine fermentation to propanoate and acetate). The simulatedMG samples shared the same pathways, but all nine simulatedMG samples were missing the butyrate pathway GLUDEG-II-PWY (l-glutamate degradation VII to butanoate), and all nine were missing the propionate pathway PROPFERM-PWY (l-alanine fermentation to propanoate and acetate). The metatranscriptomes were missing pathways that were present in the MGs and simulatedMGs, and three pathways were found in all of the samples in every group: P161-PWY, PWY-5100, and ANAEROFRUCAT-PWY. Five pathways in the MTs, P162-PWY, P163-PWY, GLUDEG-II-PWY, PWY-5677, and PROPFERM-PWY, were missing in the majority of the samples. The pathways producing lactate were the most abundant; of the five pathways that yield lactate, two were present in all nine samples in each group, two were present in eight of the MTs, and one was present in seven of the MTs. The five lactate-producing pathways were present in all of the MGs and simulatedMGs.

**FIG 6:**
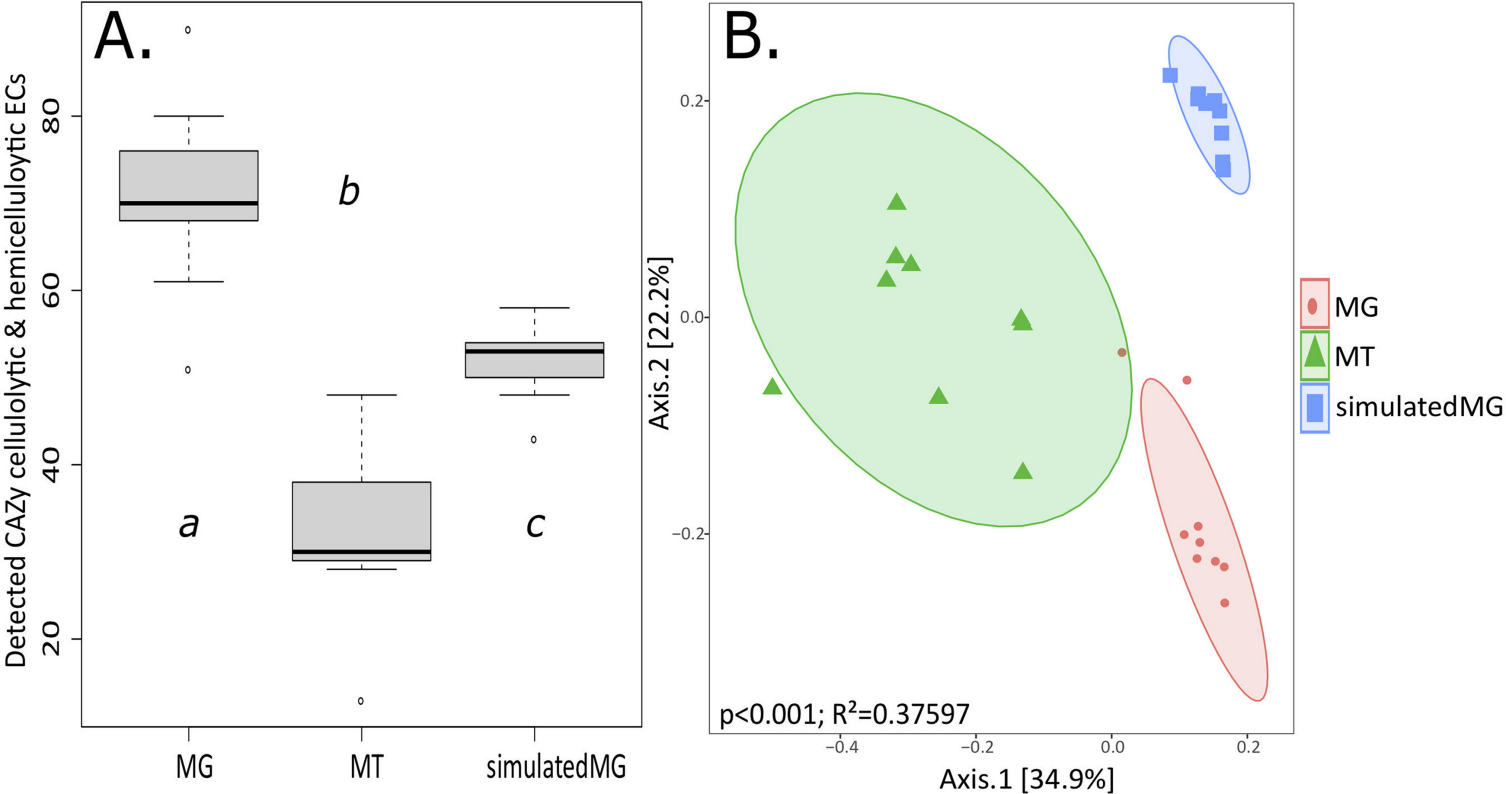
Cellulolytic and hemicellulolytic CAZy ECs. (A) Box plot of the total ECs identified in each data type: MGs, MTs, and simulatedMGs. Significance was identified at a *P* value of <0.01 using the Wilcoxon rank-sum *t* test, and significance is denoted between groups by “a,” “b,” or “c.” (B) PCoA of the Jaccard distances between the three groups showing distinct clustering between the cellulolytic and hemicellulolytic ECs found in each group; 95% confidence intervals are included in the PCoA ordination. PERMANOVA shows significant differences between the three groups (*P* < 0.001).

We identified ECs that were associated with the breakdown of dietary fibers, specifically cellulose and hemicellulose. We searched the Carbohydrate-Active enZymes Database (CAZy) for glycoside hydrolases (GH) associated with the cellulase and hemicellulase family of enzymes (49, 50). We visualized the difference in identified ECs in each group by generating a box plot (Fig. 6A). We found a significant difference in the total ECs identified between each group (Wilcoxon rank-sum *t* test; MG:MT, *P* = 0.0012; MG:simulatedMG, *P* = 0.0027; MT:simulatedMGs, *P* = 0.0013). We identified 102 ECs; the MGs had a total of 97 ECs, and 41 of those ECs were found in all nine samples. The MTs had 67, but only 6 ECs were found in every MT. The simulatedMGs identified 66, with 48 being present in all the simulatedMGs. We found that 35 ECs were shared by every MG and the simulatedMGs. Only 20 ECs were found in at least half of the MGs, MTs, and simulatedMGs. The MGs had 73 ECs that were present in at least 5 samples, the MTs had 29, and the simulatedMGs had 50. The simulatedMGs only identified 60.27% (44) of the ECs that were identified in the MGs (51). See Table S3 for all 102 ECs. We then compared the number of shared ECs between groups by calculating the Jaccard distances between each group and visualized the groups using a PCoA ordination (Fig. 6B). There were distinct separations between the MGs, MTs, and simulatedMGs. Significance between the three groups was tested using a PERMANOVA, and significant differences were observed between the groups (*P* < 0.001, *R*^2^ = 0.37597).

We specifically looked for cellulase (EC 3.2.1.4), which breaks down the cellulose found in plants. Cellulase was found in all of the MGs and all of the simulatedMGs and in eight of the MTs (Table S3). We also looked into enzymes associated with hemicellulose degradation, EC 3.2.1.8 (endo-1,4-beta-xylanase) and EC 3.2.1.37 (xylan 1,4-beta-xylosidase), which were both found in all the MGs and simulatedMGs. We identified EC 3.2.1.8 in five of the MTs and EC 3.2.1.37 in four of the MTs. Many of the ECs that appear relevant to plant fiber degradation were not consistently identified in all the samples; for example, EC 3.2.1.91 (cellulose 1,4-beta-cellobiosidase) breaks down the nonreducing end of cellulose, cleaving a glucose molecule, and was found in five of the MGs and eight of the simulatedMGs and was absent in the MTs. EC 3.2.1.131 (xylan, alpha-1,2-glucuronosidase) breaks down the chain linking the different strands of xylans found in plants and was found in all of the MGs and one of the MTs and was not identified in any of the simulatedMGs.

### Antibiotic resistance genes.

Antimicrobial resistance (AMR) KOs were pulled from the KEGG database (43, 44). We collected 162 unique KOs that were associated with aminoglycosides, fosfomycin, macrolide-lincosamide-streptogramin, penicillin, phenicol, quinolone, rifamycin, sulfonamide, tetracycline, trimethoprim, and vancomycin. The abundance of antibiotic resistance genes was compared between the three data sets. In our total data set, we identified 90 KOs that correspond to the 11 antibiotic groups. The simulatedMGs had at least one sample with a KO from 1 of the 11 antibiotic groups. The MGs did not have any KOs that were associated with fosfomycin, methicillin, sulfonamide, or trimethoprim. The MTs did not have any KOs associated with aminoglycosides, fosfomycin, methicillin, quinolone, sulfonamide, and trimethoprim (Table S4). The simulatedMGs had the most KOs; 82 of the 90 KOs were present in at least one sample versus the MGs, which had 66, and the MTs with only 20 KOs detected in at least on sample. The simulatedMGs identified 24.24% more KOs than the MGs and 310% more KOs than the MTs.

**FIG 7:**
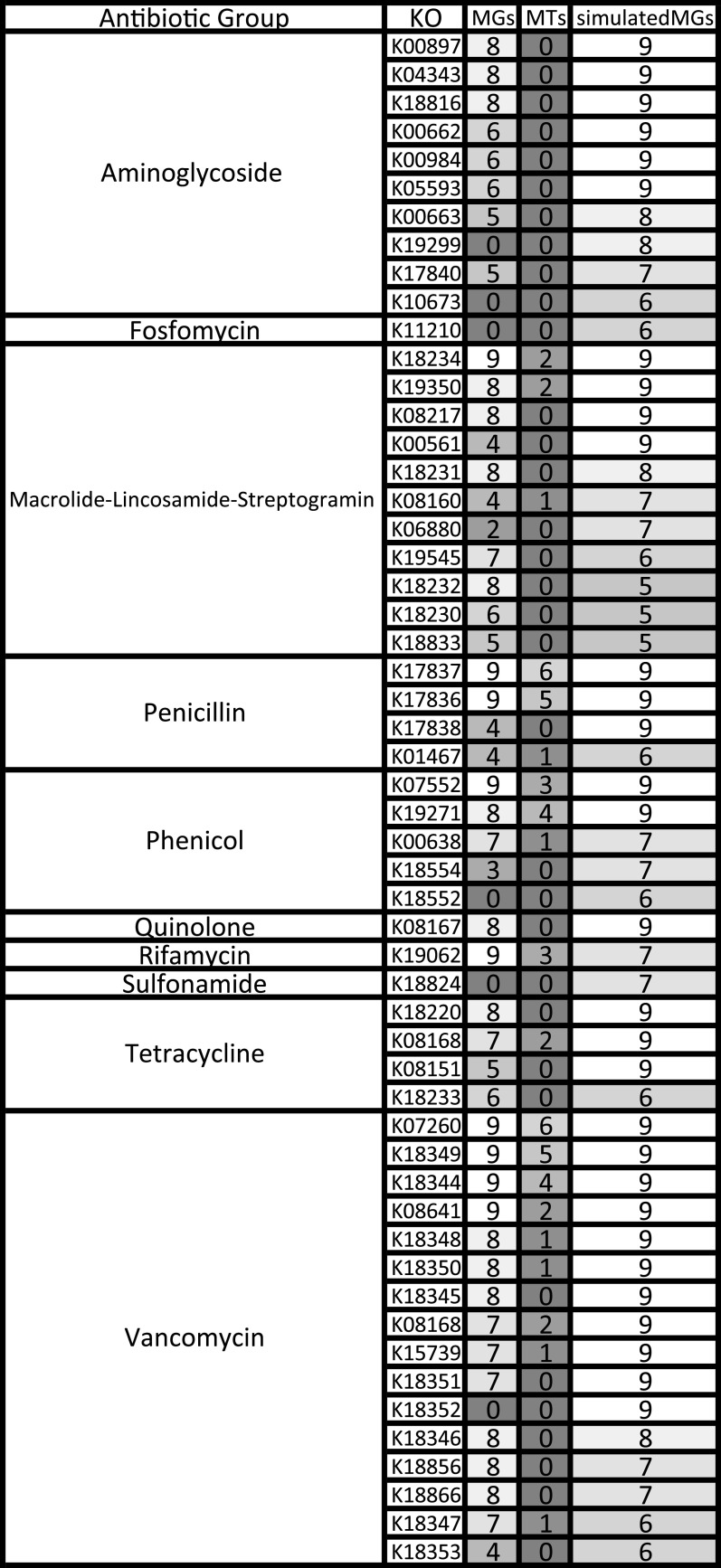
Heat map of 54 of the 90 AMR KOs showing prevalence per each data type: MGs, MTs, and simulatedMGs. KOs presented were found in five or more samples from at least one data type. White indicates KO found in every sample, and dark gray indicates KO not found in any sample.

The MGs shared the same KOs as the simulatedMGs, except that the MGs had 7 KOs that were missing in the simulatedMGs. These KOs present in MGs and not the simulatedMGs were associated with macrolide-lincosamide-streptogramin, penicillin, and aminoglycoside resistance. There were no KOs that were solely found in the MTs; all 20 KOs from the MTs were found in either the MGs or simulatedMGs. The most prevalent antibiotic KOs were associated with vancomycin, phenicol, and penicillin. The MGs identified 41 KOs that were present in at least five samples (Fig. 7). The simulatedMGs identified 54 KOs that were present in at least five samples. The simulatedMGs accurately predicted all KOs found in the MGs. Thirteen KOs were not present in at least five of the MGs; however, only 6 of the 13 were completely absent and not present in any of the MGs. The MTs only had four KOs that were present in more than half of the samples. Two KOs belonged to penicillin, and the other two were associated with vancomycin resistance.

### Taxon-specific contributions to the microbiome.

**FIG 8:**
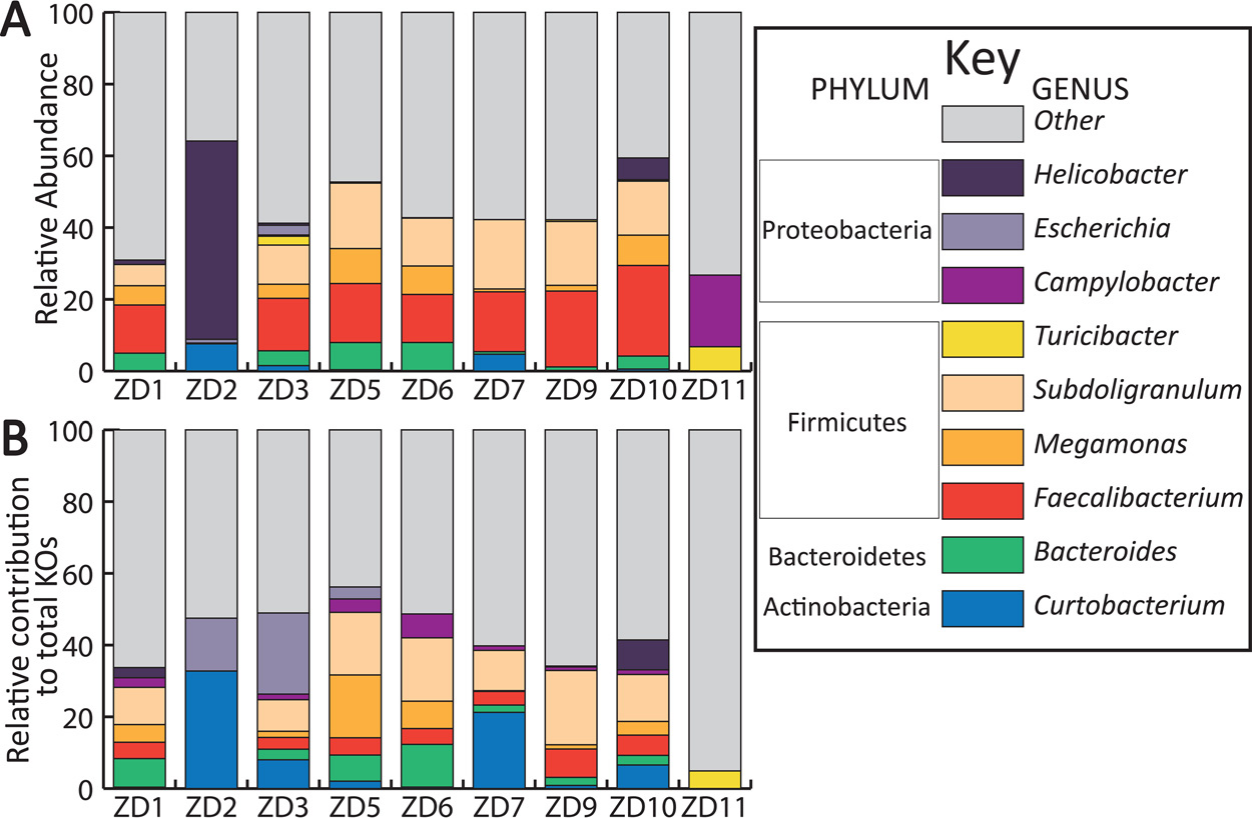
Genera-level relative abundance bar chart and relative contribution of KOs by the nine genera. (A) Genus-level relative abundance for each sample. The genera presented are those that belonged to the 17 genera that were greater than 5% of the relative abundance and were identified in the taxonomically annotated KO table generated by HUMAnN2. Relative abundance was calculated using the 16S rRNA marker sequences used to make the simulatedMGs. (B) Relative contribution of KOs of the nine genera. Samples labels denote ZymoBionics DNA extract (ZD) + sample number (1 to 12).

We identified 17 genera that had a relative abundance greater than 5% in the 16S rRNA data. KOs that originated from 1 of those 17 genera were pulled from the taxonomically labeled KO table produced by HUMAnN2. Nine of the 17 genera were identified: *Bacteroides*, Campylobacter, *Curtobacterium*, Escherichia, *Faecalibacterium*, *Helicobacter*, *Megamonas*, *Subdoligranulum*, and *Turicibacter* in the KO data. The relative abundance of those nine genera in each sample comprised 26.8% to 64.1% (*x̄* = 44.7%) of the genera identified (Fig. 8). See Table S5 for the complete genus-level relative abundance.

We then compared the KOs that originated from those nine genera and quantified the relative contribution to the total number of KOs detected in the MGs (Fig. 8). KOs from seven genera were found in the majority of the MGs: *Bacteroides* (7), Campylobacter (7), *Curtobacterium*, *Faecalibacterium*, *Megamonas*, and *Subdoligranulum*. Three metagenomes had KOs associated with Escherichia and *Helicobacter*. One sample had only *Turicibacter*-associated KOs. The nine genera were responsible for contributing 4.9% to 56.2% (*x̄* = 44.7%); see Table S6 for a complete breakdown of each genus’s contribution of KOs.

## DISCUSSION

Using the fecal microbiome of the Canada goose, we sought to characterize the function (using the metatranscriptome; “MT”), functional potential (using the metagenome; “MG”), and the taxa (using 16S rRNA gene sequencing; “16S”) of a complex wild animal’s microbiota. We also sought to determine the accuracy and utility of using metagenome simulation software in a nonmodel organism (using Picrust2 [26]; “simulatedMGs”). We annotated the -omics data into three groups: KEGG orthologies (KOs), KEGG enzyme commission numbers (ECs), and MetaCyc pathways (47, 52, 53). To determine the accuracy of predictions, we compared the raw numbers of identified KOs, ECs, and pathways in each data type (MGs, MTs, and simulatedMGs). We then looked into specific groups of ECs, KOs, and pathways to determine the accuracy of biologically relevant genes and pathways in the simulatedMGs. Some of these groups included antimicrobial resistance genes (AMRs) and genes and pathways (short-chain fatty acids [SCFAs]) critical for digestion in herbivores (3, 48, 54, 55).

PICRUSt2 is a computational alternative to metagenomic sequencing with high accuracy in many systems (26); however, due to its inherent dependence on genomic databases that largely rely on microbiota associated with humans and model organisms, simulations of nonmodel organisms’ and environmental microbiomes appear to be less accurate (26, 28). Our simulatedMGs did not show a significant difference in the number of KOs and ECs identified compared to the MGs, whereas, the simulatedMGs contained significantly fewer pathways than the MGs. Notably, because PICRUSt2 relies on 16S rRNA data, the simulatedMGs only include bacteria and archaea. When we removed all of the nonprokaryotic KOs, ECs, and pathways, the simulatedMGs contained significantly more KOs than the MGs, and the significant difference in the number of pathways was no longer present between the simulatedMGs and the MGs (Fig. 2). The MTs consistently had significantly fewer KOs, ECs, and pathways than the simulatedMGs and MGs, irrespective of the presence or absence of nonprokaryotic data. Thus, for these data, PICRUSt2 overpredicted the number of KOs but identified commensurate prokaryotic ECs and pathways with MGs. It is possible that the difference observed in KOs could be the result of insufficient sequencing depth in the MGs. We generated rarefaction plots of the KOs and showed that almost all of the MGs and MTs plateaued (Fig. S2 in the supplemental material); therefore, we do not think increasing the sequencing will rectify the discrepancy between the simulatedMGs and the sequenced data. The low number of identified KOs, ECs, and pathways in the MTs was not a surprise, as not all genes and pathways are actively transcribed at the same time and thus were not detected. It is also crucial to note that these comparisons were conducted with relatively low sequence identification rates in the metagenomes (*x̄* = 40.78%) and metatranscriptomes (*x̄* = 30.69%). The number of KOs, ECs, and pathways is most likely incomplete, and we are only observing a fraction of the functional potential of the microbiome. It is likely that many of the unknown sequences are novel or uncharacterized, and it is reasonable to assume that if these unknown sequences were identified, the number of KOs, ECs, and pathways would likely increase to levels that would far exceed PICRUSt2’s reported KOs, ECs, and pathways.

Principal coordinate analysis (PCoA) showed distinct and generally nonoverlapping clusters in the MGs, MTs, and simulatedMGs (Fig. 3). Significant differences between the data types were observed for the KO, EC, and pathway data (PERMANOVA, *P* < 0.001). These differences were observed regardless of the presence or absence of the nonprokaryotic data. We thus conclude that the nonprokaryotic KOs, ECs, and pathways affect the raw counts (discussed above), but their removal does not change the significant differences observed between the MGs, MTs, and simulatedMGs, as whole samples. The simulatedMGs and MGs shared large portions of KOs, ECs, and pathways (Table 1); however, there were many KOs, ECs, and pathways that were found in only the MGs and the simulatedMGs (Fig. 4). The MTs shared lower percent similarity with the MGs and simulatedMGs; however, this is likely due to the identification of KOs, ECs, and pathways that were not being transcribed. Furthermore, the greater identification of KOs, ECs, and pathways by the MGs and simulatedMGs is suggested by the fact that, on average, only 23 KOs, 7 ECs, and 2 pathways were found in only the MTs. This suggests that the majority of the KOs, ECs, and pathways found in the MTs were identified by the MGs, simulatedMGs, or both. Therefore, with these data, our simulatedMGs overpredict and fail to predict numerous KOs, ECs, and pathways compared to sequenced data.

Folivore diets are inherently nutrient poor, and organisms that consume large quantities of grasses and other leafy plants rely on their microbiome to breakdown the cellulolytic or hemicellulolytic foodstuffs into digestible components that the host can then absorb (3, 49). The fermentation of dietary fibers into SCFAs has generally been reported in foregut- or hindgut-fermenting organisms and are characterized by long passage time of the ingested food, frequently requiring several hours to a few days to fully digest (3, 56). Conversely, geese have significantly shorter passage times, ranging from a few hours to as short at 30 min, and show limited microbial fermentation of plant matter in their gastrointestinal tracts (55, 57). It has been suggested that geese rely primarily on a high-volume strategy where they extract small amounts of nutrients from copious amounts of food and use their proventriculus and gizzard to chemically digest and physically crush the plant matter (55, 58). Our MGs and MTs identified numerous ECs that were related to cellulose and hemicellulose degradation as well as SCFA pathways commonly found in ruminants and other herbivores (3, 48, 59). Therefore, the Canada goose fecal microbiome has the potential to ferment using a plethora of pathways; we consistently found 16 SCFA pathways that produce acetate, butyrate, propionate, or lactate in most of the MGs and MTs (Fig. 5). The majority of the MTs were missing four pathways that were present in the majority of the MGs, but our data show that the Canada goose microbiome is actively producing acetate, butyrate, lactate, and propionate (Fig. 5). We suspect that the discrepancy between the MTs and the MGs is because either the necessary substrates were not present and thus there was no need for the particular pathway or because of the pathway quantification in HUMAnN2. HUMAnN2 requires at least one read for each step of the pathway to be present for it to be counted; therefore, it is possible that one of the necessary enzymes in the pathway might have already been present in sufficient quantities, and thus there were no active transcriptions occurring, leading to the pathways appearing as absent. The simulatedMGs identified 14 of the 16 pathways found in the MGs, with two notable exceptions. PROFERM-PWY (l-alanine fermentation to propionate and acetate) and GLUDEG-II-PWY (l-glutamate degradation VII to butanoate) were identified in most of the MGs but were not found in any of the simulatedMGs. The simulatedMGs were less accurate with identifying ECs relevant to fiber digestion and more accurate with identifying complete SCFA pathways.

The presence of SCFA pathways in the MGs and MTs suggests that the Canada goose microbiome is both capable of and is actively fermenting the food they ingest, which is contrary to the accepted dietary strategy for many geese. For example, the white-fronted goose (*Anser albifrons*), the bean goose (*Anser fabalis*), and the swan goose (*Anser cygnoides*) have all shown limited ability to ferment plant matter (60); however, the Taihu goose (*Anser cygnoides*) (61) does use microbial-dependent fermentation (55). It is possible that microbial fermentation is species dependent and varies across goose species. Canada geese most likely rely on the high-volume strategy and mechanical means to break down their food, but our data suggest that they may also benefit from their microbiome’s fermentation of the ingested plant matter. Because we do see that fermentation is occurring in an organism with a quick passage time, further study is necessary to determine speed and efficiency of the Canada goose’s microbiome as well as what bacteria are participating.

Antimicrobial resistance is a growing concern in the medical community, and infection caused by an antibiotic-resistant bacterium can lead to dangerous medical situations and can be lethal (62). Because of the dangers posed by antibiotic resistance, it is crucial to describe and understand reservoirs of antimicrobial resistance genes (AMRs). Due to the frequency of defecation and their preferred habitat, geese can potentially introduce numerous antibiotic-resistant bacteria in close proximity to humans. We compiled AMR KOs from the KEGG database and found 101 AMR KOs. More than half of these (55) were found in at least five samples in the MGs, MTs, or simulatedMGs. Vancomycin, aminoglycosides, and macrolide-lincosamide-streptogramin KOs were the most frequently detected AMRs in the Canada goose. The simulatedMGs overpredicted the amount of AMR KOs, identifying 24.24% more AMR KOs than the MGs (Table S4). One possible explanation for the overprediction is that antibiotic resistance in bacteria is of interest to human health, and there may be a bias toward sequencing the genomes of AMR bacteria. This could have led to an overrepresentation of bacteria with AMR genes deposited in databases used for metagenome predictions.

The Canada goose microbiome had large quantities of *Actinobacteria*, *Proteobacteria*, and *Firmicutes*. The majority of the MTs were dominated by viruses and were also missing *Bacteroidetes*, which were found in large quantities in the MGs and simulatedMGs. In Canada geese from Maryland, Ohio, and Ontario, *Actinobacteria* was a minor phylum in the fecal microbiomes (3.8%, 11.3%, and 2.7%, respectively) (37, 40). Our data estimated *Actinobacteria* to be much more prevalent, comprising an average of 22% of the phyla identified in the MGs and 7% of the phyla identified in the simulatedMGs. It is possible that this difference is due to sampling locality; our samples originated from the northeast United States, and geographic distance is influential in the abundance of different microbes in the environment (63). *Bacteroides* appear to be a major constituent of the microbiome in Canada geese across different populations (37, 40) and in our MG and simulatedMG data as well; however, *Bacteroidetes* were not detected in our MTs. This suggests that *Bacteroidetes* might appear as key constituents of the microbiome but may not be particularly active members and could be a transient phylum. More research should be conducted to verify if *Bacteroidetes* are important members of the goose microbiome.

Our results support existing data that show the microbiome of Canada geese to be less diverse and with lower bacterial loads than other birds (32). We were able to get high genus-level resolution, identifying 17 genera at greater than 5% of the overall relative abundance; of those, nine were responsible for large portions of the total KOs identified in the metagenomes (Fig. 7). Many of the genera identified contain species that are coliforms and pathogens, for example, *Helicobacter*, Escherichia/*Shigella*, Campylobacter, *Bacteroides*, and *Clostridium*. One of the more consistent genera across the samples was *Subdoligranulum*, a close relative of *Faecalibacterium*, which has been primarily found in humans and is characterized as a butyrate producer (64). When we compared the relative contribution of these nine genera to the total KOs identified, we saw that *Subdoligranulum* and *Faecalibacterium* were responsible for a large portion of the identified KOs in the MGs. Other genera that contributed large portions of the identified KOs were *Turicibacter*, *Megamonas*, *Helicobacter*, Escherichia, *Curtobacterium*, Campylobacter, and *Bacteroides*. Our data show that a few genera can be responsible for the large portions of the identified KOs in most of our samples (Fig. 7). This suggests that in most Canada geese, these genera are key members and are responsible for large portions of the functional potential of the microbiome. This could vary from population to population; little is known about the compositional variation of the Canada goose’s microbiome across its entire range. A common axiom in microbiology is “a diverse microbiome is important for gut health.” Our data suggest that in the Canada goose, having a diverse microbiome might not be as critical as in other organisms, and the processes needed for goose health may be attained from relatively few taxa.

Viral pathogens in Canada goose feces are also of interest to the public and can be identified using -omics methods. Characterization of the viromes in the MGs and MTs (Fig. 1C) found that *Bromoviridae* was the most abundant virus family, with a *x̄* of 43.7% for MTs and a *x̄* of 55.4% for MGs. *Bromoviridae* are plant RNA viruses, and we speculate that we detected *Bromoviridae* in the MGs because the viruses might have been undergoing reverse transcription. The next most abundant virus family was *Alphaflexiviridae*, with a *x̄* of 16.3% for MTs, and *Retroviridae*, with a *x̄* of 12.5% for MGs. We did not find any viruses that were of concern to human health, but there were some viruses in trace amounts that can infect geese, for example, UR2 sarcoma virus (*Retroviridae*), goose adenovirus A (*Adenoviridae*), avian myelocytomatosis (*Retroviridae*), and avian sapelovirus (*Picornaviridae*). We also detected plant viruses, such as peanut stunt virus (*Bromoviridae*), tobacco ringspot virus (*Secoviridae*), and tobacco mosaic virus (*Virgaviridae*) (Table S7). Therefore, Canada geese feces may be a potential vector for agricultural crop viruses. More work is needed to fully assess the vector potential for Canada geese on crop production as well as other avian pathogens.

### Conclusion.

Taxa and functions are equally important aspects of a host-associated microbiome, and to fully understand the microbiome, we must determine what methods are best for identifying taxa and function in the microbiomes of nonmodel organisms. In this work, we compared the accuracy of different -omics-based methods to simulated metagenomes generated by PICRUSt2 in the Canada goose fecal microbiome. The Canada goose microbiome had few taxa that contributed a large amount to the microbiome’s functional potential, and we found plant-degrading, fermentation, and antimicrobial resistance genes. We found a significant difference between the simulated metagenomes and the sequenced -omics data at different functional levels (ECs, KOs, and metabolic pathways). The simulated metagenomes accurately predicted most pathways that were identified in the metagenomes and metatranscriptomes; however, it was less accurate when trying to identify functionally relevant genes for the goose’s digestion (underestimated) or antimicrobial resistance genes (overestimated). The discrepancies discovered here are likely due to deficiencies or biases in public databases and demonstrate the need for more whole-metagenome sequencing from different sources to improve the accuracy of future predictions in nonmodel systems.

## MATERIALS AND METHODS

### Sample collection.

For the comparative -omics analyses, 12 fecal samples were collected and stored in 9 mL of ZymoBionics DNA/RNA shield, preserving both DNA and RNA (Connecticut, *n* = 9, and New York, *n* = 3; Zymo Research, Irvine, CA, USA). Samples were collected in the summer of 2018 with the Connecticut Department of Energy and Environmental Protection (DEEP) and New York Department of Environmental Management (DEM). Fecal samples were collected from molting resident (nonmigratory) Canada geese during routine annual banding. The sex, age, and location were recorded for each sample (Table S8 in the supplemental material). While Canada geese were being held for banding, sterile weigh boats were held under each goose to catch the fecal pellet. Sterile tweezers were used to collect a portion of the fecal pellet. Samples were stored on ice until they could be transferred to a −80°C freezer.

### Extraction.

The 12 samples preserved in DNA/RNA shield were first vortexed at maximum speed for 1 min to resuspend the bacteria. The samples were then allowed to sit on the benchtop for 5 min to allow the heavier plant matter to settle on the bottom. One milliliter of the supernatant was aliquoted into a new 1.5-mL microcentrifuge tube. The samples were extracted using the standard protocol of ZymoBionics dual DNA and RNA extraction kit. Negative extraction controls were included in each extraction step.

### Total RNA and genomic DNA quality control (QC).

Purified genomic DNA was quantified using the double-stranded DNA (dsDNA) high-sensitivity assay for Qubit 3.0 (Life Technologies, Carlsbad, CA, USA). To assess fragmentation, genomic DNA was analyzed on an Agilent TapeStation 4200 (Agilent Technologies, Santa Clara, CA, USA) using the genomic DNA assay (Agilent Technologies, Santa Clara, CA, USA). DNA integrity numbers (DINe) were recorded for each sample. Total RNA was quantified, and purity ratios were determined for each sample using a NanoDrop 2000 spectrophotometer (Thermo Fisher Scientific, Waltham, MA, USA). To assess RNA quality, total RNA was analyzed on an Agilent TapeStation 4200 (Agilent Technologies, Santa Clara, CA, USA) using the RNA high-sensitivity assay following the manufacturer’s protocol. Ribosomal integrity numbers (RINe) were recorded for each sample. Three samples had insufficient DINe and RINe values for successful metagenomic and metatranscriptomic sequencing.

### Illumina metagenome and metatranscriptome library preparation and sequencing.

Genomic DNA samples were normalized to 0.2 ng/μL in preparation for whole-genome shotgun library preparation using the Illumina Nextera XT library preparation kit following the manufacturer’s protocol (Illumina, San Diego, CA, USA). Libraries were validated for length (average library length of 450 bp; average insert size of 315 bp) and adapter dimer removal using an Agilent TapeStation 4200 D5000 high-sensitivity assay (Agilent Technologies, Santa Clara, CA, USA) and then quantified and normalized using the dsDNA high-sensitivity assay for Qubit 3.0 (Life Technologies, Carlsbad, CA, USA).

Total RNA samples (250 ng of Qubit-quantified total RNA input) were prepared for prokaryotic/eukaryotic transcriptome sequencing using the Illumina Ribo-Zero total RNA library preparation kit (Illumina, San Diego, CA) following the manufacturer’s protocol. Libraries were validated for length and adapter dimer removal using an Agilent TapeStation 4200 D1000 high-sensitivity assay (Agilent Technologies, Santa Clara, CA, USA) and then quantified and normalized using the dsDNA high-sensitivity assay for Qubit 3.0 (Life Technologies, Carlsbad, CA, USA).

Sample libraries were prepared for Illumina sequencing by denaturing and diluting the libraries per the manufacturer’s protocol (Illumina, San Diego, CA, USA). All samples were combined into one sequencing pool, proportioned according to expected number of reads, and run as one sample pool on the Illumina HiSeq. Target read depth was achieved per sample with paired-end 150-bp reads.

### 16S rRNA sequencing.

The reserved DNA extracts not used for metagenomic sequencing were used to amplify and sequence the V4 region of the 16S rRNA gene at the University of Connecticut Microbial Analysis, Resources and Services center, using the standard protocols. Extracts were quantified using the Quant-iT PicoGreen kit (Invitrogen, Thermo Fisher Scientific). Partial bacterial 16S rRNA genes (V4, 0.8 pmol each of 515F and 806R with Illumina adapters and 8-bp dual indices [65]) were amplified in 15-μL reactions (done in triplicate) using GoTaq (Promega) with the addition of 10 mg of bovine serum albumin (BSA; New England BioLabs). The 515F and 806R primers (0.1 fmol), which did not have the barcodes and adapters, were added to overcome initial primer binding inhibition, because the majority of the primers do not match the template priming site. The PCR reaction mixture was incubated at 95°C for 2 min, with 30 cycles of 30 s at 95.0°C, 60 s at 50.0°C, and 60 s at 72.0°C, followed by final extension at 72.0°C for 10 min. PCR products were pooled, quantified, and visualized using QIAxcel DNA fast analysis (Qiagen). PCR products were combined using a QIAgility robot for liquid handling after the products were normalized based on the concentration of DNA from 350 to 420 bp. The pooled PCR products were cleaned using Mag-Bind RxnPure Plus (Omega Bio-tek) according to the manufacturer’s protocol. The cleaned pool was sequenced on an Illumina MiSeq using a v.2 2_250 base pair kit (Illumina, Inc.). Two PCR controls were also sequenced to test for PCR reagent contamination.

### Sequence quality control and data processing.

The quality of the nine metagenomes and nine metatranscriptomes was analyzed using FastQC (66). Low-quality reads were trimmed using Trimmomatic (67) paired-end trimming, with a minimum phred score of 33, and reads less than 35 bp were removed. The quality-controlled metagenomes and metatranscriptomes were analyzed using HUMAnN2 v. 2.0 (53). HUMAnN2’s taxonomic profiling used MetaPhlAn v. 2.0 (68), and a translated protein search was done using the uniref50 reference database. The standard gene families identified were given as a UniProt ID. The gene families were converted and regrouped into KEGG orthologies (KOs) and KEGG enzyme commission numbers (ECs) (43–45, 52). The completed pathway abundance data were annotated using HUMAnN2’s standard Metabolic Pathway Database (MetaCyc) (47, 69). The KOs, ECs, and pathways for each sample were grouped and split based on stratification, producing a file that had only the KOs, ECs, and pathways present (unstratified) as well as files that had the organism-specific annotations (stratified).

Sequence processing for 16S rRNA samples was done using R v.4.1.0 (70). Sequences were quality controlled, denoised, and merged using DADA2 v.1.20.0 (71) to create a sample-by-amplicon sequence variant (ASV) matrix. An ASV is an operational taxonomic unit, defined as any unique sequence that passes stringent quality control. Taxonomy of ASVs was assigned using RDP’s naive Bayesian classifier with the Silva reference database v128 (72, 73). Sequences that were identified as mitochondria or chloroplasts or that were unable to be confidently assigned to any bacterial phylum were removed. To remove likely contaminants, we processed the sequences using the Decontam package v.1.12.0 (51), which uses the negative controls to identify likely contaminants. To calculate phylogenetic diversity metrics, we performed a multiple alignment of all ASVs using the DECIPHER package in R (74) and constructed a phylogenetic tree with the phangorn package v.2.4.0 (75).

The sequence files were converted into biom files in R. PICRUSt2 (26) v2.4.1 was used to generate the predicted metagenomes (simulatedMGs) using the ASV biom file. PICRUSt2 generated EC, KO, and MetaCyc pathway abundance tables for all 12 DNA extracts and were merged with the nine metagenome and metatranscriptome abundance tables. The three simulatedMGs that did not have complementary MGs or MTs were removed.

### Filtering eukaryotic and viral pathways.

Eukaryotic and viral pathways found in the metagenomes and metatranscriptomes were removed to assess the effects of nonprokaryotes between the simulatedMGs and the sequenced data. After merging the pathway tables, all the pathways that were shared by the simulatedMGs and the MGs and MTs were identified. Because the simulatedMGs were derived from 16S data, we assumed that if the same pathways were found in the MGs and the MTs, they were derived from prokaryotes. We then compiled the pathways that were found in either the MGs or MTs and not in the simulatedMGs. We wrote a Python datamining script to extract the taxonomic data from each pathway’s respective HTML file using each pathways’ associated MetaCyc URL. The text files were searched using regular expressions to find all text associated with taxonomic rank. The UniProt IDs and MetaCyc pathways that contained “Bacteria” or “Archaea” were coalesced into our “prokaryotic-only” data set. We were liberal in our classification; if a pathway was found in both prokaryotes and eukaryotes, we kept it with the prokaryotic data. The remaining data included UniProt IDs and MetaCyc pathways that were associated with plants, animals, other eukaryotes, or viruses or did not contain specific taxonomic data. The total data set and prokaryote-only EC, KO, and pathway data were used for downstream analysis. The datamining script used public packages available with Anaconda v.2.1.1 (76), including Beautiful soup (77), numpy (78), and pandas (79). All code is available in our GitHub public repository (80).

### Data analyses.

The three simulatedMGs that did not have complementary MGs or MTs were removed for all comparisons. HUMAnN2 and MetaPhlAn (68) were used to produce a taxonomic profile for each sample. All -omics samples were merged, downloaded, reformatted, and combined with the phylum and family relative abundance data from the 16S sequences. To compare the viruses in the MGs and MTs, the bacteria were removed from both the MGs and MTs, leaving only the viruses. The viruses were then converted to relative abundances.

To compare the number of ECs, KOs, and pathways identified in the simulatedMGs to the MGs and MTs, box and whisker plots were generated by summing the number ECs, KOs, and pathways in each sample for both the total and the prokaryote-only data sets. Significant differences were determined using a Wilcoxon rank-sum *t* test.

The EC, KO, and pathway tables were reformatted to be analogous to otu_tables and tax_table used in the R packages phyloseq v.1.36.0 (81) and vegan v.2.5.7 (82) and were used to make a phyloseq object. Jaccard similarity index was calculated to compare the KOs, ECs, and pathways found in each of the three groups (simulatedMGs, MGs, and MTs). The matrix produced by the Jaccard similarity index was then plotted on a principal coordinate analysis (PCoA) ordination plot. Significance between the simulatedMGs, MGs, and MTs was calculated using a permutational multivariant analysis of variance test (PERMANOVA) using adonis in the vegan package in R (82).

For percent similarities between methods (simulatedMG:MG, MG:MT, simulatedMG:MT, and simulatedMG:MG:MT), UpSet plots were constructed using the UpSetR package in R v.1.4.0 (83) for both the total and the prokaryote-only data sets. Results of the UpSet plots were transcribed to a table for every sample. We averaged the ECs, KOs, and pathways found in each region of the UpSet plots and then calculated the percent similarity for each intersection of the UpSet plots. We calculated similarity using the following equation: % similarity = (2[∑ of the intersection])/(2[∑ of the intersection] + [∑ of the remaining values outside the intersections]) * 100.

Based on the Canada goose diet, genes and pathways found in herbivores were selected for additional analysis, as they could be crucial for the breakdown of fibrous plant matter. First, the MetaCyc database was used to find all pathways involved in short-chain fatty acid (SCFA) metabolism, as they are critical in the breakdown of dietary fiber into absorbable fatty acids (48, 54, 55). A list of all the SCFAs that produce acetate, butyrate, lactate, or propionate was produced. We determined how many samples contained at least one of the completed pathways and compared the prevalence of the pathways between the MGs, MTs, and the simulatedMGs. Second, the specific enzymes that are critical for the breakdown of plant matter were investigated. Using the Carbohydrate-Active Enzyme database (CAZy), all the ECs associated with cellulose degradation in CAZy’s glycoside hydrolase (GH) groups 5, 6, and 7 (50, 84) were compiled. One hundred and two ECs were extracted from the EC tables and quantified for the MGs, MTs, and simulatedMGs. The total numbers of identified ECs were plotted and further visualized with a PCoA ordination using Jaccard index. Significance between number of identified ECs was calculated using the Wilcoxon rank-sum *t* test, and significance between groups was calculated using a PERMANOVA. Third, antimicrobial resistance (AMR) genes were identified using the KEGG Orthology (KO) database. One hundred and sixty-two KOs were associated with one of the following antimicrobials: aminoglycosides (39), fosfomycin (5), macrolide-lincosamide-streptogramin (19), methicillin (3), penicillin (66), phenicol (8), quinolone (2), rifamycin (4), sulfonamide (3), tetracycline (11), trimethoprim (6), and vancomycin (18). This list was cross-referenced with the KO table, and any matches were extracted for comparisons of the AMR profile. To assess the accuracy of AMR detection, the presence of each antimicrobial was recorded and compared for each of the three methods (simulatedMGs, MGs, and MTs). The AMR KOs were compared individually (example: sample 1, MGs versus MTs versus simulatedMGs), and each sample was merged to be compared between groups (averaged: MGs versus MTs versus simulatedMGs).

HUMAnN2 (53) provides taxonomic assignments for the functional data in the form of a stratified data table, where genus and species are assigned to a functional annotation when possible. To see if the bacteria that comprised a large majority of the microbiome were likewise responsible for providing the majority of the KOs, the taxa at greater than 5% of the relative abundance in each sample in the nine 16S rRNA samples were compiled. The genera greater than 5% were searched for in the KO table. The relative contributions of each genus for each sample were calculated by ∑ genera-specific KOs/∑ all KOs.

### Ethics approval and consent to participate.

The Connecticut Department of Energy and Environmental Protection and New York Department of Environmental Management hold all necessary state and federal permits to handle Canada geese. The research was not subject to the University of Connecticut’s Institutional Animal Care and Use Committee because the fecal samples were collected noninvasively.

### Data availability.

All data generated and analyzed during this study are included in this published article, its supplementary information files, and in the https://github.com/joshuacgil/multi-omics-CAGO-microbiome-code-.git repository (80). Sequence files are archived in the SRA under the BioProject ID PRJNA879251.
