# Supplementary material for: Multiomics Characterization of the Canada Goose Fecal Microbiome Reveals Selective Efficacy of Simulated Metagenomes
Source: Microbiol Spectr. 2022 Nov 1;10(6):e02384-22. doi: 10.1128/spectrum.02384-22 (PMC9769641; doi:10.1128/spectrum.02384-22)
Supplement: Supplemental file 1 — Fig. S1 and S2 and Tables S1 to S8. Download spectrum.02384-22-s0001.pdf, PDF file, 1.0 MB [file spectrum.02384-22-s0001.pdf]

Additional files for Gil & Hird, "Multi-omics characterization of the Canada goose fecal microbiome reveals selective efficacy of simulated metagenomes"

Figure S1. PCoA ordinations of the Jaccard similarity of the ECs (Left) in three data types and Jaccard similarity of the pathways (Right) in three data types. Distinct clustering observed between the three data types and PERMANOVA test shows significance, $p < 0.001$.

Figure S2. Rarefaction curve of the observed KO reads per kilobase (RPKs). The three data types are grouped by color MG = red, MT = green, and simulatedMG = blue. X-axis shows the sampling depth of curve ranging from 0-5,000,000 RPKs. Y-axis is the number of unique KOs identified at the different KO RPK depths. Plateauing event observed for most samples.

Table S1. Phylum level relative abundance with averaged relative abundance and standard deviation.

Table S2. Virus only family level relative abundance. Families were pulled and relative abundance was recalculated.

Table S3. CAZy enzymes prevalence heat map. Glycoside hydrolase (GH) groups 5, 6, and 7; enzyme associated with cellulolytic and hemicellulolytic activity. Samples in dark gray are found in zero samples, whereas white is found in all samples.

Table S4. Prevalence of antimicrobial resistance (AMR) associated KOs.

Table S5. Genus level relative abundance and standard deviation: Derived from the nine 16S rRNA samples. Genera less than .1% of the relative abundance in each sample are grouped. Averaged relative abundance and standard deviation (std dev) included.

Table S6. Relative contribution of 'core genera' to the total number of KOs identified.

Table S7. MetaPhlAn bug list of the nine MGs and nine MTs.

Table S8. Metadata file used for the analyses.

Figure S1. PCoA ordinations of the Jaccard similarity of the ECs (left) and pathways (right) in three data types. Distinct and significant clustering observed between the data types (PERMANOVA p<0.001).

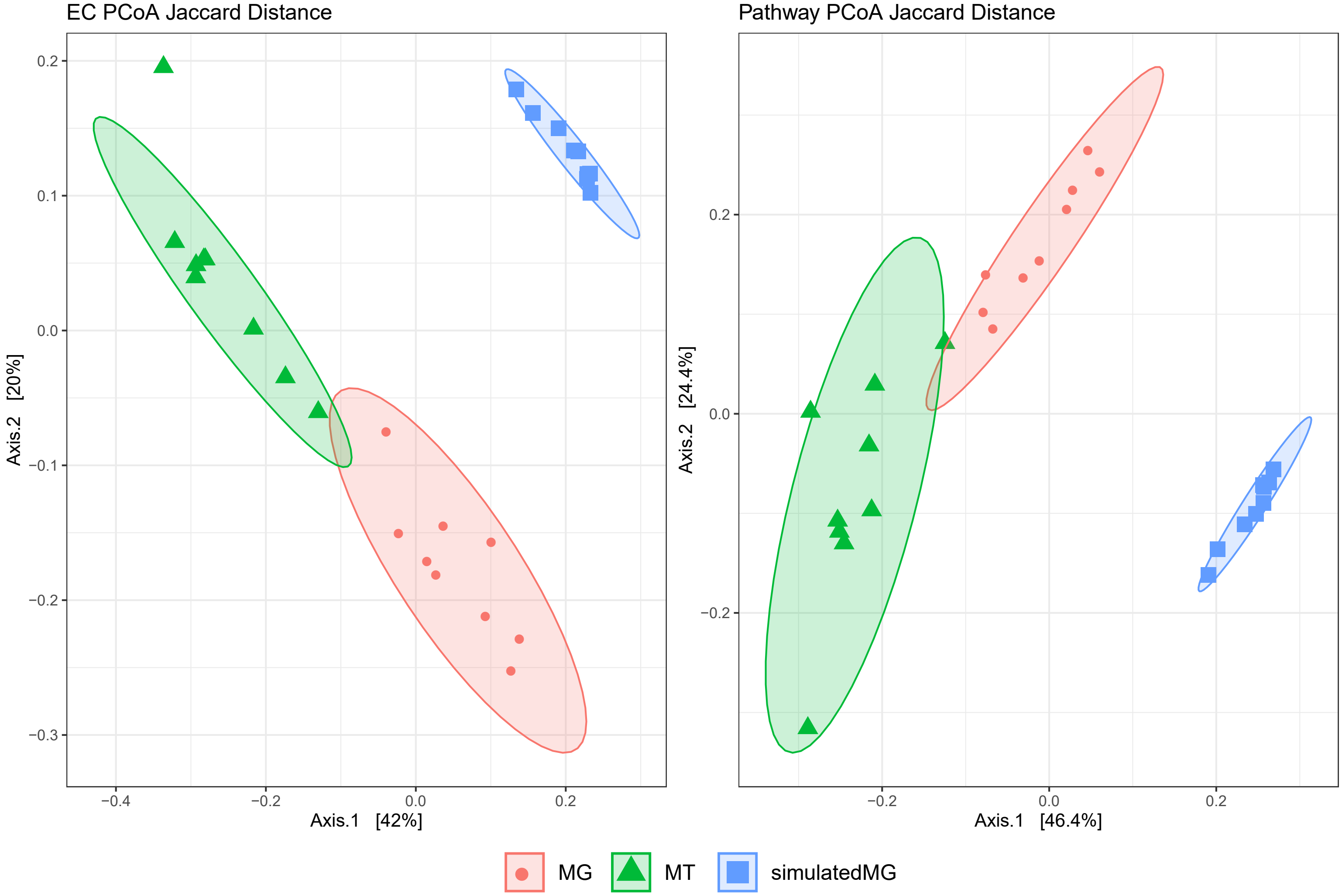

NEXT PAGE: Figure S2. Rarefaction curve of the observed KO reads per kilobase (RPKs). The three data types are grouped by color MG = red, MT = green, and simulatedMG = blue. X-axis shows the sampling depth of curve ranging from 0- 5,000,000 RPKs. Y-axis is the number of unique KOs identified at the different KO RPK depths. Plateauing event observed for most samples.

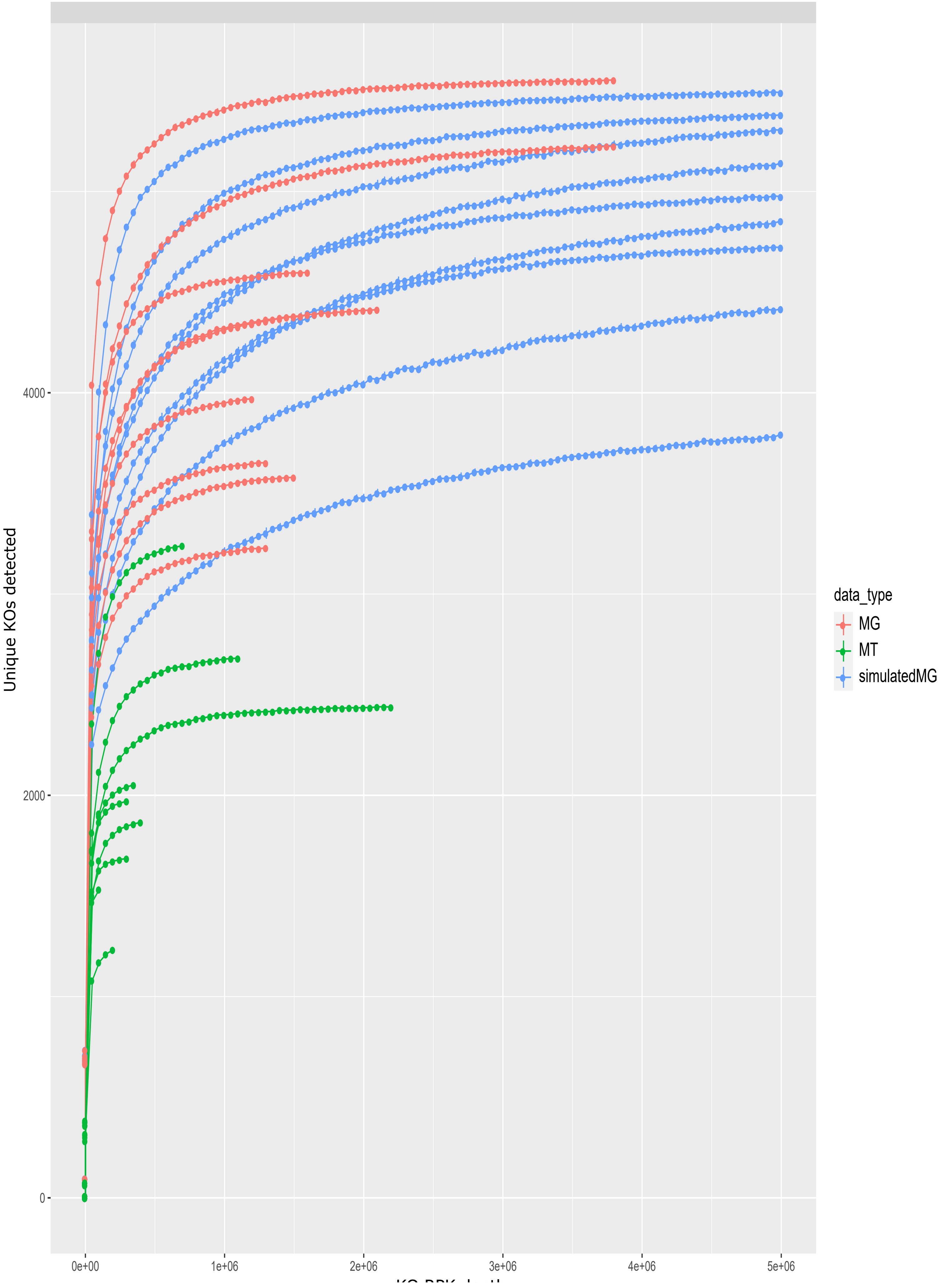
Unique KOs detected
0
2000
4000
0e+00
1e+06
2e+06
3e+06
4e+06
5e+06
data_type
MG
MT
simulatedMG

Supplemental Table 1: Phylum level relative abundance with averaged realative abundance and standard deviation.

SampleID	Actinobacteria	Bacteroidetes	Proteobacteria	Deferribacteres	Firmicutes	Spirochaetes	Deinococcus_Thermus	k__Eukaryota|p__Microsporidia	Tenericutes	Cyanobacteria	Viruses_noname	Parabasalia	Euryarchaeota	Fusobacteria	Phyla < 1 % abund.
**SimulatedMGs**															
Z1	0	0.387186328	0.031367176	0.038451832	0.506652005	0	0	0	0	0	0	0	0.010131237	0	0.026211422
Z2	0.099206073	0	0.575910579	0	0.21585765	0	0	0	0.108155164	0	0	0	0	0	0.000870534
Z3	0.047546127	0.182415776	0.059124524	0.013707328	0.670874729	0	0	0	0	0	0	0	0.010831404	0	0.015500112
Z5	0.061703242	0.142965248	0.014086967	0	0.763606729	0	0	0	0	0.010419698	0	0	0	0	0.007218115
Z6	0.159960859	0.182467047	0	0	0.649743855	0	0	0	0	0	0	0	0	0	0.007828239
Z7	0.189079684	0.054553347	0.016640942	0	0.727763843	0	0	0	0	0	0	0	0	0	0.011962184
Z9	0.065282424	0.089090684	0.022799285	0	0.739596056	0	0	0	0	0	0	0.041155541	0.031207406	0	0.010868603
Z10	0.059291349	0.061748351	0.073322126	0	0.780809518	0	0	0	0	0.013190224	0	0	0	0	0.011638433
Z11	0	0	0.200252242	0	0.628900411	0	0	0	0	0	0	0	0	0.16180867	0.009038677
**simulatedMGs average**	7.578552867	12.22696423	11.03893157	0.579546222	63.15338662	0	0	0	1.201724044	0.262332467	0	0.457283789	0.579667189	1.797874111	1.123736878
**simulatedMGs std dev**	6.085923125	11.40659365	17.41495719	1.23114571	16.69772256	0	0	0	3.398988884	0.495104506	0	1.293393872	0.995239123	5.085155903	0.652599523
**MGs**															
ZD1	2.02772	3.90665	19.61615	32.74782	39.1449	0	0	0	0	0	2.55675	0	0	0	0
ZD2	77.17361	0	16.75943	0	3.71808	0	0	0	0	0	0	0	0	0	0
ZD3	19.45662	7.34637	31.60727	0.78266	38.14196	0	0	0	0	0	2.66513	0	0	0	0
ZD5	7.58227	3.77744	3.03519	0.04654	82.37795	2.05348	0	0	0	0	1.12714	0	0	0	0
ZD6	5.75252	3.09091	3.00672	0	83.14701	0	0	0	0	0	5.00283	0	0	0	0
ZD7	56.17629	8.50167	1.36995	0	30.09612	0	0.12236	0	0	0	3.7336	0	0	0	0
ZD9	9.29476	5.86998	3.02209	0.92847	74.42587	0	0	0	0	0	6.45884	0	0	0	0
ZD10	13.70277	0.30231	24.83581	0.86187	18.14118	0	0	0.3076	0	0	41.84846	0	0	0	0
ZD11	0	0	0	0	97.60236	0	0	0	0.07504	0.34458	1.97802	0	0	0	0
**MG average**	21.24072889	3.643925556	11.47251222	3.929706667	51.86615889	0.228164444	0.013595556	0.034177778	0.008337778	0.038286667	7.263418889	0	0	0	0
**MG std dev**	25.3824897	2.988432369	11.18632357	10.19616466	31.25154584	0.645346504	0.038454038	0.096669354	0.023582797	0.108291047	12.36476134	0	0	0	0
**MTs**															
ZR1	0.01662	0.0013	0.09632	0.06171	0.34829	0	0	0	0	0	99.47576	0	0	0	0
ZR2	14.85175	0	7.65955	0	10.99065	0	0	0	0	0	66.49805	0	0	0	0
ZR3	0.00105	0.00071	0.00457	8.00E-05	0.23485	0	0	0	0	0	99.75874	0	0	0	0
ZR5	4.93216	0.61268	9.65563	0.03564	55.69019	4.78845	0	0	0	0	24.28525	0	0	0	0
ZR6	0.16245	0	0.69723	0	0.92816	0	0	0	0	0	98.21216	0	0	0	0
ZR7	0.00035	0.00021	0.00216	0	0.00205	0	0	0	0	0	99.99524	0	0	0	0
ZR9	0.00334	0	0.00709	0.00048	0.06631	0	0	0	0	0	99.92278	0	0	0	0
ZR10	0.00095	0	0.01456	0.00011	0.02955	0	0	0	0	0	99.95483	0	0	0	0
ZR11	97.06429	0	0.09387	0	0	0	0	0	0	0	2.84185	0	0	0	0
**MT average**	13.00366222	0.068322222	2.025664444	0.010891111	7.587783333	0.53205	0	0	0	0	76.77162889	0	0	0	0
**MT std dev**	30.08238073	0.192460006	3.581969594	0.021110913	17.3367457	1.504864652	0	0	0	0	35.65627971	0	0	0	0

Supplemental Table 2: Virus only family level relative abundance. Families were pulled and relative abundance was recalculated.

Sample ID	*Secoviridae*	*Alphaflexiviridae*	*Betaflexiviridae*	*Tymoviridae*	*Adenoviridae*	*Bromoviridae*	*Hepadnaviridae*	*Luteoviridae*	*Partitiviridae*	*Retroviridae*	*Virgaviridae*	Viruses_noname	Virus Less than 1%
MGs													
ZD1	0	0	0	0	0	71.452	0	0	0	0	0	28.548	0
ZD10	0	0	0	0	3.821	1.596	93.208	0	0	0	0	0	1.375
ZD11	0	20.840	0	0	0	63.146	0	0	0	16.014	0	0	0
ZD2	0	0	0	0	0	43.648	0	0	0	56.352	0	0	0
ZD3	0	0	0	0	0	52.415	0	0	0	8.395	1.967	37.223	0
ZD5	0	0	0	0	0	87.343	0	0	0	12.657	0	0	0
ZD6	0	0	0	0	0	62.036	0	0	0	0	4.915	33.048	0
ZD7	0	0	0	0	0	49.505	0	0	0	10.937	0	39.558	0
**ZD9**	0	0	0	0	0	68.126	0	0	0	7.679	0	24.195	0
**MGs average**	0	2.316	0	0	0	55.474	10.356	0	0	12.448	0	18.064	0.153
**MGs std dev**	0	6.549	0	0	1.201	22.658	29.292	0	0	16.488	1.590	16.688	0.432
ZR1	0	15.464	2.633	0	0	80.994	0	0	0	0	0	0	0.908
ZR10	0	2.656	57.490	0	0	29.698	0	0	0	0	0	9.845	0.310
ZR11	0	0	0	0	0	29.751	64.678	0	0	5.570	0	0	0
ZR2	0	0	0	0	0	53.978	0	0	0	46.022	0	0	0
ZR3	0	0	0	0	0	98.890	0	0	0	0	0	0	1.110
ZR5	3.877	0	0	0	0	37.348	0	0	0	0	58.776	0	0
ZR6	0	36.358	0	0	0	53.383	0	0	0	0	0	8.865	1.394
ZR7	0	1.885	0	0	0	0	0	0	0	0	0	97.556	0.559
ZR9	0	89.476	0	0	0	9.892	0	0	0	0	0	0	0.632
**MTs average**	0.431	16.204	6.680	0	0	43.770	7.186	0	0	5.732	6.531	12.919	0.546
**MTs std dev**	1.218	28.312	17.983	0	0	30.037	20.326	0	0	14.350	18.471	30.168	0.486

Supplemental table S3: CAZy enzymes prevalance heat map. Glycoside hydrolase (GH) groups 5, 6, and 7; enzyme associated with cellulolytic and hemicellulolytic activity. Samples in dark gray are found in zero samples, whereas white is found in all samples.

CAZy GH ECs	MGs	MTs	simulatedMGs
3.2.1.123	2	0	1
3.2.1.132	4	0	2
3.2.1.151	3	3	0
3.2.1.21	9	8	9
3.2.1.25	8	5	9
3.2.1.39	7	3	0
3.2.1.4	9	8	9
3.2.1.45	9	4	9
3.2.1.46	0	1	2
3.2.1.52	9	9	9
3.2.1.55	9	9	9
3.2.1.58	1	0	9
3.2.1.73	9	4	1
3.2.1.74	8	3	0
3.2.1.75	2	0	0
3.2.1.78	9	4	9
3.2.1.8	9	5	9
3.2.1.91	5	0	5
3.2.1.1	9	8	9
3.2.1.10	9	7	9
3.2.1.101	1	0	0
3.2.1.103	7	2	0
3.2.1.106	1	0	0
3.2.1.11	3	0	1
3.2.1.113	7	4	0
3.2.1.119	0	1	0
3.2.1.122	9	7	9
3.2.1.125	1	0	0
3.2.1.130	1	0	0
3.2.1.131	9	1	0
3.2.1.133	7	1	0
3.2.1.135	9	6	9
3.2.1.136	0	0	6
3.2.1.139	9	0	9
3.2.1.14	9	8	9
3.2.1.141	8	0	9
3.2.1.142	4	0	0
3.2.1.143	7	1	0
3.2.1.147	2	3	0
3.2.1.15	9	5	3
3.2.1.152	5	0	0
3.2.1.153	1	1	0
3.2.1.154	1	2	0
3.2.1.155	1	0	0
3.2.1.156	8	0	8
3.2.1.165	7	0	0
3.2.1.169	8	1	0
3.2.1.17	9	5	9
3.2.1.170	8	0	9
3.2.1.171	1	0	0
3.2.1.172	6	0	9

CAZy GH ECs	MGs	MTs	simulatedMGs
3.2.1.177	9	3	9
3.2.1.179	7	0	0
3.2.1.18	9	2	9
3.2.1.180	9	3	9
3.2.1.183	3	1	4
3.2.1.184	1	0	8
3.2.1.185	5	0	4
3.2.1.187	2	0	0
3.2.1.2	9	8	0
3.2.1.20	9	9	9
3.2.1.22	9	9	9
3.2.1.23	9	9	9
3.2.1.24	9	2	9
3.2.1.26	9	8	9
3.2.1.28	7	1	8
3.2.1.3	9	6	4
3.2.1.31	9	8	9
3.2.1.33	8	3	0
3.2.1.35	9	1	9
3.2.1.36	1	0	0
3.2.1.37	9	4	9
3.2.1.38	5	0	0
3.2.1.40	9	5	9
3.2.1.41	9	7	9
3.2.1.48	1	0	0
3.2.1.49	8	8	0
3.2.1.50	7	5	8
3.2.1.51	9	8	9
3.2.1.54	9	4	7
3.2.1.59	1	0	0
3.2.1.6	8	2	0
3.2.1.64	5	0	5
3.2.1.65	9	7	8
3.2.1.67	8	2	0
3.2.1.68	8	0	0
3.2.1.70	9	6	9
3.2.1.76	0	0	0
3.2.1.80	8	2	9
3.2.1.81	1	0	0
3.2.1.82	7	2	0
3.2.1.84	3	1	0
3.2.1.85	7	1	7
3.2.1.86	9	9	9
3.2.1.89	9	2	9
3.2.1.93	9	6	9
3.2.1.96	9	1	4
3.2.1.97	0	0	8
3.2.1.98	7	0	0
3.2.1.99	9	1	9
3.2.1.n1	7	2	0
3.2.1.n2	7	2	0

Supplemental table S4: Prevalence of antimicrobial resistance (AMR) associated KOs.

Antibiotic Group	KO	MGs	MTs	simulatedMGs
Aminoglycoside	K00662	6	0	9
	K00663	5	0	8
	K00897	8	0	9
	K00984	6	0	9
	K03395	2	0	0
	K04343	8	0	9
	K05593	6	0	9
	K10673	0	0	6
	K17840	5	0	7
	K17880	1	0	4
	K17910	0	0	3
	K18815	0	0	4
	K18816	8	0	9
	K18844	2	0	4
	K18846	1	0	2
	K19272	1	0	0
	K19273	0	0	4
	K19299	0	0	8
	K19300	1	0	4
Fosfomycin	K11210	0	0	6
Macrolide-Lincosamide-Streptogramin	K00561	4	0	9
	K06880	2	0	7
	K06979	0	0	3
	K08160	4	1	7
	K08217	8	0	9
	K14336	3	0	0
	K15632	0	0	1
	K18230	6	0	5
	K18231	8	0	8
	K18232	8	0	5
	K18234	9	2	9
	K18235	4	0	4
	K18236	0	0	4
	K18833	5	0	5
	K19349	4	0	0
	K19350	8	2	9
	K19545	7	0	6
Methicillin	K02545	0	0	2
	K02546	0	0	2
	K02547	0	0	2
	K01467	4	1	6
	K17836	9	5	9

Penicilin	K17837	9	6	9
	K17838	4	0	9
	K18698	1	0	0
	K18767	2	0	0
	K18768	3	0	0
	K19098	1	0	0
	K19212	0	0	2
	K19215	0	0	1
Phenicol	K00638	7	1	7
	K07552	9	3	9
	K15632	0	0	1
	K18552	0	0	6
	K18553	4	0	1
	K18554	3	0	7
	K19271	8	4	9
	K19646	0	0	2
Quinolone	K08167	8	0	9
Rifamycin	K19062	9	3	7
Sulfonamide	K18824	0	0	7
	K18974	0	0	1
Tetracycline	K08151	5	0	9
	K08168	7	2	9
	K18214	0	0	1
	K18215	4	0	1
	K18216	3	0	1
	K18217	2	0	1
	K18220	8	0	9
	K18233	6	0	6
Trimethoprim	K18590	0	0	2
	K18591	3	0	1
Vancomycin	K07260	9	6	9
	K08168	7	2	9
	K08641	9	2	9
	K15739	7	1	9
	K18344	9	4	9
	K18345	8	0	9
	K18346	8	0	8
	K18347	7	1	6
	K18348	8	1	9
	K18349	9	5	9
	K18350	8	1	9
	K18351	7	0	9
	K18352	0	0	9
	K18353	4	0	6
	K18354	0	0	2

	K18856	8	0	7
	K18866	8	0	7
	K18906	0	0	2

Additional table S5: Genus level relative abundance: Derived from the nine 16S rRNA samples. Genera less than .1% of the relative abundance in each sample

	ZD1	ZD10	ZD11	ZD2	ZD3	ZD5	ZD6	ZD7	ZD9	**average**	**std dev**
Faecalibacterium	13.4278	25.2613	0.0000	0.0000	14.6328	16.4499	13.3895	16.6897	21.1377	13.4432	8.0368
Subdoligranulum	5.9232	15.0523	0.0000	0.0000	10.9078	18.2493	13.3549	19.3307	17.8117	11.1811	7.1321
Helicobacter	1.2265	6.0408	0.0000	55.2694	0.5693	0.0000	0.0000	0.0000	0.0000	7.0118	17.1611
Clostridium_sensu_stricto_1	0.0000	0.5359	22.7803	19.0763	0.0000	0.0000	0.0000	1.1107	0.0000	4.8337	8.6544
Megamonas	5.3810	8.4491	0.0000	0.0000	3.9314	9.7393	7.9240	0.7961	1.6081	4.2032	3.6157
Prevotella_7	27.5435	0.0000	0.0000	0.0000	0.0000	2.9990	2.3720	0.0000	0.0000	3.6572	8.5173
Bacteroides	4.8805	3.6258	0.0000	0.0000	4.1339	7.5776	7.8375	0.7103	0.9900	3.3062	2.9061
Terrisporobacter	0.0000	0.8204	26.6504	0.1183	0.5120	0.0000	0.0000	0.0000	0.2781	3.1532	8.3119
Family_Lachnospiraceae	2.6040	3.1229	0.2957	0.1705	2.3573	5.4813	4.7614	2.4932	3.3349	2.7357	1.6634
Campylobacter	0.0000	0.1919	19.9744	0.1009	0.3439	0.0000	0.1097	0.0000	0.0000	2.3023	6.2490
Sellimonas	1.7233	1.9783	0.0000	0.0000	4.3440	3.2068	2.7587	2.4074	2.6424	2.1179	1.3357
Prevotellaceae_Ga6A1_group	1.3539	0.6881	0.0000	0.0000	4.9935	0.4276	5.7829	1.9069	3.1029	2.0284	2.0303
Fusobacterium	0.0000	0.0000	16.0671	0.0000	0.0000	0.0000	0.0000	0.0000	0.0000	1.7852	5.0494
Curtobacterium	0.1055	0.5558	0.0000	7.6048	1.5168	0.3801	0.1385	4.7004	0.1700	1.6858	2.5202
Family_Ruminococcaceae	2.8188	2.5010	0.0000	0.0000	1.1003	1.4431	1.4832	2.2453	2.5043	1.5662	0.9960
Pseudoflavonifractor	1.5086	1.8327	0.0000	0.0000	1.6314	1.1283	4.3170	2.2119	1.3513	1.5535	1.2113
Bifidobacterium	0.0000	0.5492	0.0000	0.0000	0.0000	1.9657	10.3596	0.1907	0.7527	1.5353	3.1776
Ruminiclostridium_9	1.3375	1.5681	0.0000	0.0000	3.5264	0.8017	0.9754	3.1511	2.1802	1.5045	1.1827
Senegalimassilia	0.3876	1.6673	0.0000	0.0000	0.5081	1.0868	1.1081	5.9208	2.2528	1.4368	1.7368
Genus < .1 % abund.	1.4176	1.4358	0.2911	1.3643	2.2159	0.7839	0.6695	1.7734	1.8012	1.3058	0.5804
Ureaplasma	0.0000	0.1853	0.0000	10.7963	0.1872	0.0000	0.0000	0.3242	0.0000	1.2770	3.3675
Turicibacter	0.0000	0.2382	6.7808	0.1114	2.4719	0.0000	0.0000	0.0000	0.4693	1.1191	2.1372
Dialister	1.7142	1.2703	0.0000	0.0000	0.7565	1.0036	2.3489	0.7198	1.8703	1.0760	0.7635
Romboutsia	0.0000	0.0000	3.8002	0.0000	4.8636	0.0000	0.0000	0.0000	0.2444	0.9898	1.8055
Solobacterium	0.0000	1.1049	0.0000	0.0000	0.0000	3.7235	2.1065	0.8867	0.5118	0.9259	1.1951
Butyricicoccus	1.0645	0.7873	0.0000	0.0000	0.8023	1.2946	1.1196	2.2072	0.9759	0.9168	0.6312
Alistipes	1.2156	0.0000	0.0000	0.0000	2.5904	0.7601	0.0000	1.8592	1.5532	0.8865	0.9166
Rikenellaceae_RC9_gut_group	0.5514	0.3705	0.0000	0.0000	2.7088	1.2471	0.0000	0.4004	2.1023	0.8201	0.9346
Ruminococcaceae_UCG-008	0.2420	1.3431	0.0000	0.0000	0.2407	0.6236	0.3751	2.9699	1.3425	0.7930	0.9076
Family_Coriobacteriaceae	0.1529	1.4490	0.0000	0.0000	0.7909	1.1105	0.8830	1.9736	0.6004	0.7734	0.6354
Anaerofilum	0.6023	0.7344	0.0000	0.0000	1.2111	0.5820	0.5194	1.7543	1.3124	0.7462	0.5544
Prevotellaceae_NK3B31_group	0.1656	0.0000	0.0000	0.0000	2.3573	1.4134	2.1643	0.0000	0.6039	0.7449	0.9211
Peptococcus	0.6005	0.6881	0.0000	0.0000	0.6075	0.0000	3.6186	0.0000	1.1034	0.7353	1.0877
Olsenella	0.3166	1.0255	0.0000	0.0000	0.2789	0.7067	1.6217	0.8867	1.7162	0.7280	0.6074
Megasphaera	0.9353	1.9386	0.0000	0.0000	0.0000	0.5939	1.2120	0.1335	1.2752	0.6765	0.6650
Ruminococcaceae_UCG-005	1.7579	0.2647	0.0000	0.0000	1.4022	0.8314	0.0000	0.9916	0.7261	0.6638	0.6113
Mucispirillum	3.9033	0.2382	0.0000	0.0000	1.3716	0.1010	0.0000	0.0000	0.3489	0.6626	1.2182
Family_Thermoplasmatales_Incertae_Sedis	1.0300	0.0000	0.0000	0.0000	1.1233	0.0000	0.0000	0.5292	3.1560	0.6487	0.9875
Lactobacillus	0.0000	0.1720	0.2212	0.0000	0.2063	0.0000	0.1385	4.9578	0.0000	0.6329	1.5317
Alloprevotella	2.6659	1.0454	0.0000	0.0000	0.5387	0.0000	0.3117	0.0000	0.0000	0.5069	0.8350
Erysipelotrichaceae_UCG-003	0.3694	0.7609	0.0000	0.0000	1.1194	0.8730	0.1385	0.5959	0.6482	0.5006	0.3756
Lachnospiraceae_NC2004_group	0.2839	0.4565	0.0000	0.0000	1.0239	0.7601	0.3174	0.9773	0.5349	0.4838	0.3576
Escherichia/Shigella	0.0000	0.0000	0.0000	1.0476	2.7203	0.3147	0.0000	0.0000	0.0000	0.4536	0.8657
Ruminococcus_2	0.2020	0.0000	0.0000	0.0000	0.3591	3.2722	0.0000	0.0000	0.0000	0.4259	1.0135
Sutterella	0.7516	0.4499	0.0000	0.0000	0.7297	0.2494	0.4155	0.4529	0.7208	0.4189	0.2749
Flavonifractor	0.4349	0.4433	0.0000	0.0000	0.4852	0.2910	0.2424	0.4386	1.3212	0.4063	0.3677
Order_Gastranaerophilales	0.5277	1.2703	0.0000	0.0000	0.4929	0.9086	0.0000	0.0000	0.1824	0.3758	0.4376
Desulfovibrio	0.7406	0.0000	0.0000	0.0000	0.7259	0.5998	0.0000	0.2002	1.1016	0.3742	0.3984
Erysipelotrichaceae_UCG-004	0.9572	0.5955	0.0000	0.0000	0.3897	0.4335	0.0000	0.3146	0.3967	0.3430	0.2996
Tetratrichomonas	0.5204	0.2117	0.0000	0.0000	0.1796	0.0000	0.0000	0.0000	2.0987	0.3345	0.6451
Ruminococcaceae_UCG-014	0.5969	0.6881	0.0000	0.0000	0.2407	0.6057	0.1327	0.1764	0.3471	0.3097	0.2495
Oscillospira	0.3858	0.5822	0.0000	0.0000	0.2522	0.3326	0.2424	0.3957	0.4746	0.2962	0.1866
Ruminococcaceae_UCG-007	0.1711	0.0000	0.0000	0.0000	1.5817	0.0000	0.0000	0.6197	0.1187	0.2768	0.4989
Family_Clostridiales_vadinBB60_group	0.5532	0.0000	0.0000	0.0000	0.5578	0.0000	0.0000	0.0000	1.3194	0.2701	0.4348
Class_Gf10_symbiont_group	0.1929	0.0000	0.0000	0.0000	0.0000	0.0000	0.0000	0.0000	2.1607	0.2615	0.6742
Prevotellaceae_UCG-001	1.0318	0.1125	0.0000	0.0000	0.2483	0.0000	0.3059	0.0000	0.1789	0.2086	0.3114
Enorma	0.0000	0.1191	0.0000	0.0000	0.4852	0.3385	0.3174	0.2860	0.3082	0.2060	0.1698
Anaerotruncus	0.3221	0.1456	0.0000	0.0000	0.4317	0.3266	0.1558	0.1955	0.2727	0.2056	0.1393
Slackia	0.0000	0.3573	0.0000	0.0000	0.0000	0.2138	0.3232	0.6483	0.1470	0.1877	0.2115
Frondihabitans	0.0000	0.0000	0.0000	0.3689	0.1566	0.0000	0.0000	1.1632	0.0000	0.1876	0.3647
Barnesiella	0.2402	0.0000	0.0000	0.0000	0.7679	0.1722	0.0000	0.3337	0.1488	0.1848	0.2365
Anaerostipes	0.0000	0.0000	0.0000	0.0000	0.1757	0.2969	0.7445	0.0000	0.4020	0.1799	0.2458
Lachnoclostridium	0.1219	0.2911	0.0000	0.0000	0.2369	0.2910	0.2424	0.1239	0.2798	0.1763	0.1120
Atopobium	0.0000	0.0000	0.0000	0.0000	0.0000	0.7245	0.0000	0.4529	0.3914	0.1743	0.2603

Kineococcus	0.0000	0.0000	0.0000	0.1357	0.2025	0.0000	0.0000	1.2299	0.0000	0.1742	0.3799
Enterococcus	0.0000	0.0000	0.0000	0.4768	0.8596	0.1663	0.0000	0.0000	0.0000	0.1670	0.2873
Faecalitalea	0.1237	0.4433	0.0000	0.0000	0.0000	0.1247	0.1212	0.1049	0.5437	0.1624	0.1860
Christensenellaceae_R-7_group	0.1510	0.0000	0.0000	0.0000	0.2369	0.0000	0.0000	0.5673	0.4782	0.1593	0.2111
Coprococcus_1	0.0000	0.0000	0.0000	0.0000	0.8023	0.3504	0.1501	0.0000	0.1010	0.1560	0.2539
Family_Bifidobacteriaceae	0.0000	0.0000	0.0000	0.0000	0.0000	0.0000	1.3332	0.0000	0.0000	0.1481	0.4190
Ruminococcaceae_UCG-013	0.1820	0.0000	0.0000	0.0000	0.1872	0.3741	0.3001	0.2574	0.0000	0.1445	0.1400
Family_Prevotellaceae	0.0000	0.8204	0.0000	0.0000	0.0000	0.0000	0.0000	0.0000	0.3949	0.1350	0.2718
Intestinimonas	0.2202	0.1919	0.0000	0.0000	0.2483	0.0000	0.0000	0.3671	0.1293	0.1285	0.1290
Class_Mollicutes	0.1237	0.3639	0.0000	0.0000	0.3859	0.1544	0.0000	0.1239	0.0000	0.1280	0.1447
Candidatus_Arthromitus	0.0000	0.0000	0.0000	1.0998	0.0000	0.0000	0.0000	0.0000	0.0000	0.1222	0.3456
Tyzzerella	0.4204	0.0000	0.0000	0.0000	0.0000	0.0000	0.5136	0.1144	0.0000	0.1165	0.1919
Intestinibacter	0.0000	0.5492	0.4727	0.0000	0.0000	0.0000	0.0000	0.0000	0.0000	0.1135	0.2132
Ruminiclostridium_5	0.1219	0.0000	0.0000	0.0000	0.0000	0.4335	0.0000	0.2050	0.1913	0.1057	0.1417
Blautia	0.0000	0.0000	0.0000	0.0000	0.1566	0.3326	0.0000	0.3051	0.1346	0.1032	0.1293
Lachnospiraceae_UCG-010	0.5186	0.1853	0.0000	0.0000	0.0000	0.1960	0.0000	0.0000	0.0000	0.1000	0.1672
Solibacillus	0.0000	0.0000	0.8639	0.0000	0.0000	0.0000	0.0000	0.0000	0.0000	0.0960	0.2715
Methylobacterium	0.0000	0.1125	0.0000	0.0000	0.1719	0.0000	0.0000	0.4195	0.1010	0.0894	0.1318
Neorhizobium	0.0000	0.2779	0.0000	0.3585	0.1566	0.0000	0.0000	0.0000	0.0000	0.0881	0.1335
Cellulosilyticum	0.0000	0.0000	0.7591	0.0000	0.0000	0.0000	0.0000	0.0000	0.0000	0.0843	0.2386
Family_Rhodospirillaceae	0.0000	0.1389	0.0000	0.0000	0.2560	0.2316	0.0000	0.0000	0.1169	0.0826	0.1004
Family_Microbacteriaceae	0.0000	0.1853	0.0000	0.1323	0.1643	0.0000	0.0000	0.2050	0.0000	0.0763	0.0872
Syntrophococcus	0.0000	0.0000	0.0000	0.0000	0.0000	0.0000	0.1154	0.0000	0.5561	0.0746	0.1740
Oscillibacter	0.1620	0.0000	0.0000	0.0000	0.0000	0.1247	0.0000	0.1621	0.2001	0.0721	0.0825
Order_Mollicutes_RF9	0.1856	0.0000	0.0000	0.0000	0.1452	0.3088	0.0000	0.0000	0.0000	0.0711	0.1082
Family_Pasteurellaceae	0.0000	0.0000	0.0000	0.6334	0.0000	0.0000	0.0000	0.0000	0.0000	0.0704	0.1991
Asteroleplasma	0.0000	0.5822	0.0000	0.0000	0.0000	0.0000	0.0000	0.0000	0.0000	0.0647	0.1830
Ruminococcaceae_NK4A214_group	0.0000	0.0000	0.0000	0.0000	0.0000	0.0000	0.0000	0.2813	0.2692	0.0612	0.1145
Family_XIII_UCG-001	0.0000	0.0000	0.0000	0.0000	0.1070	0.1960	0.0000	0.1096	0.1027	0.0573	0.0691
Rhodococcus	0.0000	0.0000	0.0000	0.3376	0.1643	0.0000	0.0000	0.0000	0.0000	0.0558	0.1120
Family_Erysipelotrichaceae	0.1256	0.1853	0.0000	0.0000	0.0000	0.0000	0.1270	0.0000	0.0000	0.0486	0.0706
Caproiciproducens	0.1165	0.1059	0.0000	0.0000	0.0000	0.1900	0.0000	0.0000	0.0000	0.0458	0.0683
Porphyromonas	0.0000	0.0000	0.3982	0.0000	0.0000	0.0000	0.0000	0.0000	0.0000	0.0442	0.1251
Erysipelatoclostridium	0.0000	0.2845	0.0000	0.0000	0.0000	0.1010	0.0000	0.0000	0.0000	0.0428	0.0911
Order_Bacteroidales	0.0000	0.0000	0.0000	0.0000	0.1910	0.0000	0.0000	0.1764	0.0000	0.0408	0.0765
Sanguibacter	0.0000	0.0000	0.0000	0.3411	0.0000	0.0000	0.0000	0.0000	0.0000	0.0379	0.1072
Collinsella	0.0000	0.0000	0.0000	0.0000	0.1108	0.0000	0.0000	0.0000	0.2232	0.0371	0.0743
Rothia	0.0000	0.0000	0.0000	0.1357	0.0000	0.0000	0.0000	0.1764	0.0000	0.0347	0.0656
Order_Clostridiales	0.1401	0.1389	0.0000	0.0000	0.0000	0.0000	0.0000	0.0000	0.0000	0.0310	0.0580
Lachnospiraceae_NK4A136_group	0.0000	0.0000	0.0000	0.0000	0.0000	0.0000	0.0000	0.1668	0.1116	0.0309	0.0593
Marvinbryantia	0.0000	0.0000	0.0000	0.0000	0.0000	0.0000	0.0000	0.1192	0.1399	0.0288	0.0541
Anaerobiospirillum	0.1347	0.0000	0.0000	0.0000	0.0000	0.0000	0.0000	0.0000	0.1063	0.0268	0.0505
Streptobacillus	0.0000	0.0000	0.2352	0.0000	0.0000	0.0000	0.0000	0.0000	0.0000	0.0261	0.0739
Clavibacter	0.0000	0.2183	0.0000	0.0000	0.0000	0.0000	0.0000	0.0000	0.0000	0.0243	0.0686
Candidatus_Soleaferrea	0.0000	0.0000	0.0000	0.0000	0.0000	0.0000	0.0000	0.0000	0.2161	0.0240	0.0679
Shuttleworthia	0.0000	0.0000	0.0000	0.0000	0.2101	0.0000	0.0000	0.0000	0.0000	0.0233	0.0660
Family_Micrococcaceae	0.0000	0.0000	0.0000	0.0000	0.0000	0.0000	0.0000	0.2098	0.0000	0.0233	0.0659
Akkermansia	0.0000	0.0000	0.0000	0.0000	0.0000	0.0000	0.0000	0.0000	0.2054	0.0228	0.0646
Phylum_Proteobacteria	0.1911	0.0000	0.0000	0.0000	0.0000	0.0000	0.0000	0.0000	0.0000	0.0212	0.0600
Family_P3OB-42	0.0000	0.0000	0.0000	0.0000	0.0000	0.0000	0.0000	0.1907	0.0000	0.0212	0.0599
Ruminococcaceae_UCG-004	0.0000	0.0000	0.0000	0.0000	0.0000	0.0000	0.0000	0.0000	0.1700	0.0189	0.0534
Paenibacillus	0.0000	0.0000	0.0000	0.1671	0.0000	0.0000	0.0000	0.0000	0.0000	0.0186	0.0525
Solirubrobacter	0.0000	0.0000	0.0000	0.0000	0.0000	0.0000	0.0000	0.1621	0.0000	0.0180	0.0509
Rhizobium	0.0000	0.0000	0.0000	0.1531	0.0000	0.0000	0.0000	0.0000	0.0000	0.0170	0.0481
Family_Actinomycetaceae	0.0000	0.0000	0.1490	0.0000	0.0000	0.0000	0.0000	0.0000	0.0000	0.0166	0.0468
Rikenella	0.0000	0.0000	0.0000	0.0000	0.1490	0.0000	0.0000	0.0000	0.0000	0.0166	0.0468
Cellulomonas	0.0000	0.0000	0.0000	0.0000	0.0000	0.0000	0.0000	0.1478	0.0000	0.0164	0.0464
Synechococcus	0.0000	0.0000	0.1467	0.0000	0.0000	0.0000	0.0000	0.0000	0.0000	0.0163	0.0461
Family_Intrasporangiaceae	0.0000	0.0000	0.0000	0.0000	0.0000	0.0000	0.0000	0.1287	0.0000	0.0143	0.0405
Nocardioides	0.0000	0.0000	0.0000	0.0000	0.0000	0.0000	0.0000	0.1287	0.0000	0.0143	0.0405
Victivallis	0.0000	0.0000	0.0000	0.0000	0.0000	0.0000	0.0000	0.0000	0.1240	0.0138	0.0390
Parabacteroides	0.1219	0.0000	0.0000	0.0000	0.0000	0.0000	0.0000	0.0000	0.0000	0.0135	0.0383
Ruminiclostridium	0.0000	0.0000	0.0000	0.0000	0.0000	0.0000	0.0000	0.1192	0.0000	0.0132	0.0375
Amnibacterium	0.0000	0.0000	0.0000	0.0000	0.0000	0.0000	0.0000	0.1144	0.0000	0.0127	0.0360
Mycoplasma	0.0000	0.0000	0.1141	0.0000	0.0000	0.0000	0.0000	0.0000	0.0000	0.0127	0.0359
Nakamurella	0.0000	0.0000	0.0000	0.0000	0.0000	0.0000	0.0000	0.1001	0.0000	0.0111	0.0315

Supplemental table S6: Relative contribution of 'core genera' to the total number of KOs identified

Sample ID	*Bacteroides*	*Campylobacter*	*Curtobacterium*	*Escherichia*	*Faecalibacterium*	*Helicobacter*	*Megamonas*	*Subdoligranulum*	*Turicibacter*	Other Taxa
ZD1	7.975	2.681	0.387	0	4.558	2.827	4.919	10.379	0	66.273
ZD2	0	0	32.772	14.734	0	0	0	0	0	52.494
ZD3	2.958	1.550	8.024	22.625	3.275	0	1.726	8.800	0	51.042
ZD5	7.278	3.741	2.064	3.336	4.820	0	17.510	17.458	0	43.792
ZD6	11.902	6.646	0.418	0	4.411	0	7.631	17.673	0	51.318
ZD7	2.040	1.258	21.247	0	3.811	0	0.197	11.238	0	60.209
ZD9	2.244	0.999	0.876	0	7.914	0.233	1.188	20.702	0	65.845
ZD10	2.680	1.306	6.578	0	5.647	8.321	3.814	13.091	0	58.563
ZD11	0	0	0	0	0	0	0	0	4.923	95.077

Supplemental Table S7: MetaPhlAn bug list of the nine MGs and nine MTs.

Kingdom	Phylum	Class	Order	Family	Genus	Species	Strain	ZD1	ZD10	ZD11	ZD2	ZD3	ZD5	ZD6	ZD7	ZD9	ZR1	ZR10	ZR11	ZR2	ZR3	ZR5	ZR6	ZR7	ZR9
Bacteria	Actinobacteria	Actinobacteria	Actinomycetales	Micromonosporaceae	Actinoplanes	NA	NA	0	0	0	0	0	0	0	0.11787	0	0	0	0	0	0	0	0	0	0
Bacteria	Actinobacteria	Actinobacteria	Actinomycetales	Micromonosporaceae	Actinoplanes	Actinoplanes_unclassified	NA	0	0	0	0	0	0	0	0.11787	0	0	0	0	0	0	0	0	0	0
Bacteria	Actinobacteria	Actinobacteria	Actinomycetales	Microbacteriaceae	Agromyces	NA	NA	0	0	0	0.04901	0	0	0	0.01936	0	0	0	0	0	0	0	0	0	0
Bacteria	Actinobacteria	Actinobacteria	Actinomycetales	Microbacteriaceae	Agromyces	Agromyces_unclassified	NA	0	0	0	0.04901	0	0	0	0.01936	0	0	0	0	0	0	0	0	0	0
Bacteria	Bacteroidetes	Bacteroidia	Bacteroidales	Rikenellaceae	Alistipes	NA	NA	2.33005	0.12233	0	0	6.56694	3.12911	0.13126	8.42416	5.54213	0	0	0	0	0.00021	0.60568	0	0.00021	0
Bacteria	Bacteroidetes	Bacteroidia	Bacteroidales	Rikenellaceae	Alistipes	Alistipes_unclassified	NA	2.33005	0.12233	0	0	6.56694	3.12911	0.13126	8.42416	5.54213	0	0	0	0	0.00021	0.60568	0	0.00021	0
Viruses	Viruses_noname	Viruses_noname	Viruses_noname	Retroviridae	Alpharetrovirus	NA	NA	0	0.0449	0.31676	1.32363	0.22374	0.14266	0	0.23292	0.34492	0.00974	0.00055	0.00781	30.60361	0.00057	0	0.15868	0.00224	0
Viruses	Viruses_noname	Viruses_noname	Viruses_noname	Retroviridae	Alpharetrovirus	Avian_myelocytomatosis_virus	NA	0	0	0.31676	1.32363	0.22374	0.14266	0	0	0.34492	0	0	0.00781	0	0	0	0	0	0
Viruses	Viruses_noname	Viruses_noname	Viruses_noname	Retroviridae	Alpharetrovirus	Avian_myelocytomatosis_virus	PRJNA14909	0	0	0.31676	1.32363	0.22374	0.14266	0	0	0.34492	0	0	0.00781	0	0	0	0	0	0
Viruses	Viruses_noname	Viruses_noname	Viruses_noname	Retroviridae	Alpharetrovirus	Fujinami_sarcoma_virus	NA	0	0.0449	0	0	0	0	0	0.23292	0	0	0.00055	0	0	0	0	0	0	0
Viruses	Viruses_noname	Viruses_noname	Viruses_noname	Retroviridae	Alpharetrovirus	Fujinami_sarcoma_virus	PRJNA14708	0	0.0449	0	0	0	0	0	0.23292	0	0	0.00055	0	0	0	0	0	0	0
Viruses	Viruses_noname	Viruses_noname	Viruses_noname	Retroviridae	Alpharetrovirus	UR2_sarcoma_virus	NA	0	0	0	0	0	0	0	0	0	0.00974	0	0	30.60361	0.00057	0	0.15868	0.00224	0
Viruses	Viruses_noname	Viruses_noname	Viruses_noname	Retroviridae	Alpharetrovirus	UR2_sarcoma_virus	PRJNA15322	0	0	0	0	0	0	0	0	0	0.00974	0	0	30.60361	0.00057	0	0.15868	0.00224	0
Bacteria	Firmicutes	Clostridia	Clostridiales	Ruminococcaceae	Anaerotruncus	NA	NA	0	0	0	0	0	0	0	0	0	0	0	0	0	0	0.15519	0	0	0
Bacteria	Firmicutes	Clostridia	Clostridiales	Ruminococcaceae	Anaerotruncus	Anaerotruncus_unclassified	NA	0	0	0	0	0	0	0	0	0	0	0	0	0	0	0.15519	0	0	0
Viruses	Viruses_noname	Viruses_noname	Viruses_noname	Adenoviridae	Aviadenovirus	NA	NA	0	1.59919	0	0	0	0	0	0	0	0	0	0	0	0	0	0	0	0
Viruses	Viruses_noname	Viruses_noname	Viruses_noname	Adenoviridae	Aviadenovirus	Goose_adenovirus_A	NA	0	1.59919	0	0	0	0	0	0	0	0	0	0	0	0	0	0	0	0
Viruses	Viruses_noname	Viruses_noname	Viruses_noname	Adenoviridae	Aviadenovirus	Goose_adenovirus_A	PRJNA167579	0	1.59919	0	0	0	0	0	0	0	0	0	0	0	0	0	0	0	0
Bacteria	Firmicutes	Bacilli	Bacillales	Bacillaceae	Bacillus	NA	NA	0	0	0	0	0	0	0	0.00542	0	0	0	0	0	0	0	0	0	0
Bacteria	Firmicutes	Bacilli	Bacillales	Bacillaceae	Bacillus	Bacillus_megaterium	NA	0	0	0	0	0	0	0	0.00542	0	0	0	0	0	0	0	0	0	0
Bacteria	Firmicutes	Bacilli	Bacillales	Bacillaceae	Bacillus	Bacillus_megaterium	Bacillus_megaterium_unclassified	0	0	0	0	0	0	0	0.00542	0	0	0	0	0	0	0	0	0	0
Bacteria	Bacteroidetes	Bacteroidia	Bacteroidales	Bacteroidaceae	Bacteroides	NA	NA	1.45591	0.17999	0	0	0.15878	0.64833	2.95965	0.07751	0.32785	0	0	0	0	0	0.007	0	0	0
Bacteria	Bacteroidetes	Bacteroidia	Bacteroidales	Bacteroidaceae	Bacteroides	Bacteroides_barnesiae	NA	0	0.00343	0	0	0	0	0	0	0	0	0	0	0	0	0	0	0	0
Bacteria	Bacteroidetes	Bacteroidia	Bacteroidales	Bacteroidaceae	Bacteroides	Bacteroides_barnesiae	GCF_000374585	0	0.00343	0	0	0	0	0	0	0	0	0	0	0	0	0	0	0	0
Bacteria	Bacteroidetes	Bacteroidia	Bacteroidales	Bacteroidaceae	Bacteroides	Bacteroides_coprocola	NA	0.20187	0	0	0	0.01124	0.11163	0.17449	0.00707	0.06559	0	0	0	0	0	0	0	0	0
Bacteria	Bacteroidetes	Bacteroidia	Bacteroidales	Bacteroidaceae	Bacteroides	Bacteroides_coprocola	GCF_000154845	0.20187	0	0	0	0.01124	0.11163	0.17449	0.00707	0.06559	0	0	0	0	0	0	0	0	0
Bacteria	Bacteroidetes	Bacteroidia	Bacteroidales	Bacteroidaceae	Bacteroides	Bacteroides_coprophilus	NA	0.45776	0.07302	0	0	0	0	0	0	0	0	0	0	0	0	0	0	0	0
Bacteria	Bacteroidetes	Bacteroidia	Bacteroidales	Bacteroidaceae	Bacteroides	Bacteroides_coprophilus	GCF_000157915	0.45776	0.07302	0	0	0	0	0	0	0	0	0	0	0	0	0	0	0	0
Bacteria	Bacteroidetes	Bacteroidia	Bacteroidales	Bacteroidaceae	Bacteroides	Bacteroides_plebeius	NA	0.79627	0.10354	0	0	0.14755	0.5367	2.78516	0.07044	0.26225	0	0	0	0	0	0.007	0	0	0
Bacteria	Bacteroidetes	Bacteroidia	Bacteroidales	Bacteroidaceae	Bacteroides	Bacteroides_plebeius	GCF_000187895	0.79627	0.10354	0	0	0.14755	0.5367	2.78516	0.07044	0.26225	0	0	0	0	0	0.007	0	0	0
Viruses	Viruses_noname	Viruses_noname	Viruses_noname	Partitiviridae	Betacryptovirus	NA	NA	0	0	0	0	0	0	0	0	0	0	0.06346	0	0	0.00068	0	0.06992	0.00099	0
Viruses	Viruses_noname	Viruses_noname	Viruses_noname	Partitiviridae	Betacryptovirus	Betacryptovirus_unclassified	NA	0	0	0	0	0	0	0	0	0	0	0.03254	0	0	0.00018	0	0	0	0
Viruses	Viruses_noname	Viruses_noname	Viruses_noname	Partitiviridae	Betacryptovirus	White_clover_cryptic_virus_2	NA	0	0	0	0	0	0	0	0	0	0	0.03092	0	0	0.0005	0	0.06992	0.00099	0
Viruses	Viruses_noname	Viruses_noname	Viruses_noname	Partitiviridae	Betacryptovirus	White_clover_cryptic_virus_2	PRJNA198685	0	0	0	0	0	0	0	0	0	0	0.03092	0	0	0.0005	0	0.06992	0.00099	0
Bacteria	Firmicutes	Clostridia	Clostridiales	Lachnospiraceae	Blautia	NA	NA	3.29382	1.80391	0	0	3.01831	5.61761	11.09589	1.6711	6.03641	0.18597	0.00716	0	0	0.00494	20.44276	0.84893	0.00195	0.00774
Bacteria	Firmicutes	Clostridia	Clostridiales	Lachnospiraceae	Blautia	Ruminococcus_torques	NA	3.29382	1.80391	0	0	3.01831	5.61761	11.09589	1.6711	6.03641	0.18597	0.00716	0	0	0.00494	20.44276	0.84893	0.00195	0.00774
Bacteria	Firmicutes	Clostridia	Clostridiales	Lachnospiraceae	Blautia	Ruminococcus_torques	Ruminococcus_torques_unclassified	3.29382	1.80391	0	0	3.01831	5.61761	11.09589	1.6711	6.03641	0.18597	0.00716	0	0	0.00494	20.44276	0.84893	0.00195	0.00774
Bacteria	Spirochaetes	Spirochaetia	Spirochaetales	Brachyspiraceae	Brachyspira	NA	NA	0	0	0	0	0	2.05348	0	0	0	0	0	0	0	0	4.78845	0	0	0
Bacteria	Spirochaetes	Spirochaetia	Spirochaetales	Brachyspiraceae	Brachyspira	Brachyspira_innocens	NA	0	0	0	0	0	0.12592	0	0	0	0	0	0	0	0	0	0	0	0
Bacteria	Spirochaetes	Spirochaetia	Spirochaetales	Brachyspiraceae	Brachyspira	Brachyspira_innocens	GCF_000384655	0	0	0	0	0	0.12592	0	0	0	0	0	0	0	0	0	0	0	0
Bacteria	Spirochaetes	Spirochaetia	Spirochaetales	Brachyspiraceae	Brachyspira	Brachyspira_murdochii	NA	0	0	0	0	0	0.25675	0	0	0	0	0	0	0	0	0	0	0	0

Bacteria	Spirochaetes	Spirochaetia	Spirochaetales	Brachyspiraceae	Brachyspira	Brachyspira_murdochii	GCF_000092845	0	0	0	0	0	0.25675	0	0	0	0	0	0	0	0	0	0	0	0
Bacteria	Spirochaetes	Spirochaetia	Spirochaetales	Brachyspiraceae	Brachyspira	Brachyspira_unclassified	NA	0	0	0	0	0	1.67081	0	0	0	0	0	0	0	0	4.78845	0	0	0
Viruses	Viruses_noname	Viruses_noname	Viruses_noname	Bromoviridae	Bromovirus	NA	NA	1.82685	0.66775	1.24905	1.02524	1.39692	0.98447	3.10358	1.05428	3.06025	75.91364	0.64841	0.82404	35.89444	98.59724	1.91922	50.11511	0.05426	0.30442
Viruses	Viruses_noname	Viruses_noname	Viruses_noname	Bromoviridae	Bromovirus	Brome_mosaic_virus	NA	1.82685	0.66775	1.24905	1.02524	1.39692	0.98447	3.10358	1.05428	3.06025	75.91364	0.64841	0.82404	35.89444	98.59724	1.91922	50.11511	0.05426	0.30442
Viruses	Viruses_noname	Viruses_noname	Viruses_noname	Bromoviridae	Bromovirus	Brome_mosaic_virus	PRJNA15052	1.82685	0.66775	1.24905	1.02524	1.39692	0.98447	3.10358	1.05428	3.06025	75.91364	0.64841	0.82404	35.89444	98.59724	1.91922	50.11511	0.05426	0.30442
Bacteria	Proteobacteria	Betaproteobacteria	Burkholderiales	Burkholderiaceae	Burkholderia	NA	NA	0	0	0	0	0	0	0	0.10807	0	0	0	0	0	0	0	0	0	0
Bacteria	Proteobacteria	Betaproteobacteria	Burkholderiales	Burkholderiaceae	Burkholderia	Burkholderia_unclassified	NA	0	0	0	0	0	0	0	0.10807	0	0	0	0	0	0	0	0	0	0
Bacteria	Firmicutes	Clostridia	Clostridiales	Clostridiaceae	Butyricicoccus	NA	NA	1.09409	0.29494	0	0	0.25414	0.27687	1.588	0.79154	1.25977	0	0	0	0	0	0	0	0	0
Bacteria	Firmicutes	Clostridia	Clostridiales	Clostridiaceae	Butyricicoccus	Butyricicoccus_pullicaecorum	NA	1.09409	0.29494	0	0	0.25414	0.27687	1.588	0.79154	1.25977	0	0	0	0	0	0	0	0	0
Bacteria	Firmicutes	Clostridia	Clostridiales	Clostridiaceae	Butyricicoccus	Butyricicoccus_pullicaecorum	GCF_000398925	1.09409	0.29494	0	0	0.25414	0.27687	1.588	0.79154	1.25977	0	0	0	0	0	0	0	0	0
Bacteria	Proteobacteria	Epsilonproteobacteria	Campylobacterales	Campylobacteraceae	Campylobacter	NA	NA	0	1.0137	0	0	2.12769	0	0	0	0	0	0	0	0	0.00027	0	0	0	0
Bacteria	Proteobacteria	Epsilonproteobacteria	Campylobacterales	Campylobacteraceae	Campylobacter	Campylobacter_jejuni	NA	0	1.0137	0	0	2.12769	0	0	0	0	0	0	0	0	0.00027	0	0	0	0
Bacteria	Proteobacteria	Epsilonproteobacteria	Campylobacterales	Campylobacteraceae	Campylobacter	Campylobacter_jejuni	Campylobacter_jejuni_unclassified	0	1.0137	0	0	2.12769	0	0	0	0	0	0	0	0	0.00027	0	0	0	0
Viruses	Viruses_noname	Viruses_noname	Tymovirales	Betaflexiviridae	Carlavirus	NA	NA	0	0	0	0	0	0	0	0	0	2.61901	57.46443	0	0	0.15135	0	0	0	0.01435
Viruses	Viruses_noname	Viruses_noname	Tymovirales	Betaflexiviridae	Carlavirus	Red_clover_vein_mosaic_virus	NA	0	0	0	0	0	0	0	0	0	2.61901	57.46443	0	0	0.15135	0	0	0	0.01435
Viruses	Viruses_noname	Viruses_noname	Tymovirales	Betaflexiviridae	Carlavirus	Red_clover_vein_mosaic_virus	PRJNA34841	0	0	0	0	0	0	0	0	0	2.61901	57.46443	0	0	0.15135	0	0	0	0.01435
Bacteria	Actinobacteria	Actinobacteria	Actinomycetales	Cellulomonadaceae	Cellulomonas	NA	NA	0	0.01137	0	0	0	0	0	0.18923	0	0	0	0	0	0	0	0	0	0
Bacteria	Actinobacteria	Actinobacteria	Actinomycetales	Cellulomonadaceae	Cellulomonas	Cellulomonas_unclassified	NA	0	0.01137	0	0	0	0	0	0.18923	0	0	0	0	0	0	0	0	0	0
Bacteria	Actinobacteria	Actinobacteria	Actinomycetales	Microbacteriaceae	Clavibacter	NA	NA	0.07305	7.53967	0	0.60621	0.10582	0	0.01438	0.09875	0.96487	0	0	0	0	0	0	0	0	0
Bacteria	Actinobacteria	Actinobacteria	Actinomycetales	Microbacteriaceae	Clavibacter	Clavibacter_michiganensis	NA	0.07305	7.53967	0	0.60621	0.10582	0	0.01438	0.09875	0.96487	0	0	0	0	0	0	0	0	0
Bacteria	Actinobacteria	Actinobacteria	Actinomycetales	Microbacteriaceae	Clavibacter	Clavibacter_michiganensis	Clavibacter_michiganensis_unclassified	0.07305	7.53967	0	0.60621	0.10582	0	0.01438	0.09875	0.96487	0	0	0	0	0	0	0	0	0
Bacteria	Firmicutes	Clostridia	Clostridiales	Clostridiaceae	Clostridium	NA	NA	0	0	0.58137	0	0	0	0	0	0	0	0	0.00224	0	0	0	0	0	0
Bacteria	Firmicutes	Clostridia	Clostridiales	Clostridiaceae	Clostridium	Clostridium_celatum	NA	0	0	0.15027	0	0	0	0	0	0	0	0	0	0	0	0	0	0	0
Bacteria	Firmicutes	Clostridia	Clostridiales	Clostridiaceae	Clostridium	Clostridium_celatum	GCF_000320405	0	0	0.15027	0	0	0	0	0	0	0	0	0	0	0	0	0	0	0
Bacteria	Firmicutes	Clostridia	Clostridiales	Clostridiaceae	Clostridium	Clostridium_perfringens	NA	0	0	0.43109	0	0	0	0	0	0	0	0	0.00224	0	0	0	0	0	0
Bacteria	Firmicutes	Clostridia	Clostridiales	Clostridiaceae	Clostridium	Clostridium_perfringens	Clostridium_perfringens_unclassified	0	0	0.43109	0	0	0	0	0	0	0	0	0.00224	0	0	0	0	0	0
Bacteria	Actinobacteria	Actinobacteria	Coriobacteriales	Coriobacteriaceae	Collinsella	NA	NA	0.89229	0.84683	0	0	1.69936	3.37044	2.48509	0.72533	3.69462	0.0149	0.00086	0	0	0.00094	3.85847	0.0423	2.00E-05	0.00208
Bacteria	Actinobacteria	Actinobacteria	Coriobacteriales	Coriobacteriaceae	Collinsella	Collinsella_unclassified	NA	0.89229	0.84683	0	0	1.69936	3.37044	2.48509	0.72533	3.69462	0.0149	0.00086	0	0	0.00094	3.85847	0.0423	2.00E-05	0.00208
Bacteria	Proteobacteria	Betaproteobacteria	Burkholderiales	Comamonadaceae	Comamonas	NA	NA	0	0	0	0	0	0	0	0	0	0	0	0	1.6556	0	0	0	0	0
Bacteria	Proteobacteria	Betaproteobacteria	Burkholderiales	Comamonadaceae	Comamonas	Comamonas_unclassified	NA	0	0	0	0	0	0	0	0	0	0	0	0	1.6556	0	0	0	0	0
Viruses	Viruses_noname	Viruses_noname	Viruses_noname	Bromoviridae	Cucumovirus	NA	NA	0	0	0	0	0	0	0	0	0	4.65613	29.03613	0.02145	0	0.05415	7.15079	2.31316	0	9.57994
Viruses	Viruses_noname	Viruses_noname	Viruses_noname	Bromoviridae	Cucumovirus	Cucumber_mosaic_virus	NA	0	0	0	0	0	0	0	0	0	0	0	0	0	0	7.15079	0	0	0
Viruses	Viruses_noname	Viruses_noname	Viruses_noname	Bromoviridae	Cucumovirus	Cucumber_mosaic_virus	PRJNA15470	0	0	0	0	0	0	0	0	0	0	0	0	0	0	7.15079	0	0	0
Viruses	Viruses_noname	Viruses_noname	Viruses_noname	Bromoviridae	Cucumovirus	Peanut_stunt_virus	NA	0	0	0	0	0	0	0	0	0	4.65613	29.03613	0.02145	0	0.05415	0	2.31316	0	9.57994
Viruses	Viruses_noname	Viruses_noname	Viruses_noname	Bromoviridae	Cucumovirus	Peanut_stunt_virus	PRJNA15471	0	0	0	0	0	0	0	0	0	4.65613	29.03613	0.02145	0	0.05415	0	2.31316	0	9.57994
Bacteria	Actinobacteria	Actinobacteria	Actinomycetales	Microbacteriaceae	Curtobacterium	NA	NA	0.63411	4.31521	0	69.80751	16.40817	3.21164	1.7457	50.7503	2.79648	0	0	0	0	9.00E-05	0	0	5.00E-05	0
Bacteria	Actinobacteria	Actinobacteria	Actinomycetales	Microbacteriaceae	Curtobacterium	Curtobacterium_flaccumfaciens	NA	0.22572	3.73668	0	41.42079	9.12286	2.16594	0.48306	10.27624	0.87077	0	0	0	0	0	0	0	0	0
Bacteria	Actinobacteria	Actinobacteria	Actinomycetales	Microbacteriaceae	Curtobacterium	Curtobacterium_flaccumfaciens	GCF_000349565	0.22572	3.73668	0	41.42079	9.12286	2.16594	0.48306	10.27624	0.87077	0	0	0	0	0	0	0	0	0
Bacteria	Actinobacteria	Actinobacteria	Actinomycetales	Microbacteriaceae	Curtobacterium	Curtobacterium_unclassified	NA	0.40839	0.57854	0	28.38672	7.28531	1.0457	1.26264	40.47407	1.92571	0	0	0	0	9.00E-05	0	0	5.00E-05	0
Bacteria	Cyanobacteria	Cyanobacteria_noname	Chroococcales	Chroococcales_noname	Cyanobium	NA	NA	0	0	0.34458	0	0	0	0	0	0	0	0	0	0	0	0	0	0	0
Bacteria	Cyanobacteria	Cyanobacteria_noname	Chroococcales	Chroococcales_noname	Cyanobium	Cyanobium_unclassified	NA	0	0	0.34458	0	0	0	0	0	0	0	0	0	0	0	0	0	0	0

Bacteria	Proteobacteria	Deltaproteobacteria	Desulfovibrionales	Desulfovibrionaceae	Desulfovibrio	NA	NA	0	0	0	0	0.09051	0.16372	0	0	0.1324	0	0	0	0	3.00E-05	0	0	0	0
Bacteria	Proteobacteria	Deltaproteobacteria	Desulfovibrionales	Desulfovibrionaceae	Desulfovibrio	Desulfovibrio_desulfuricans	NA	0	0	0	0	0.09051	0.16372	0	0	0.1324	0	0	0	0	3.00E-05	0	0	0	0
Bacteria	Proteobacteria	Deltaproteobacteria	Desulfovibrionales	Desulfovibrionaceae	Desulfovibrio	Desulfovibrio_desulfuricans	Desulfovibrio_desulfuricans_unclassified	0	0	0	0	0.09051	0.16372	0	0	0.1324	0	0	0	0	3.00E-05	0	0	0	0
Bacteria	Firmicutes	Clostridia	Clostridiales	Lachnospiraceae	Dorea	NA	NA	0	0	0	0	0.02052	0	0	0	0	0	0	0	0	0	0	0	0	0
Bacteria	Firmicutes	Clostridia	Clostridiales	Lachnospiraceae	Dorea	Dorea_unclassified	NA	0	0	0	0	0.02052	0	0	0	0	0	0	0	0	0	0	0	0	0
Bacteria	Firmicutes	Bacilli	Lactobacillales	Enterococcaceae	Enterococcus	NA	NA	0	0.41956	0	3.33674	7.63838	1.14519	0	0	0.15661	0	0	0	0	0.00015	0	0	0	0
Bacteria	Firmicutes	Bacilli	Lactobacillales	Enterococcaceae	Enterococcus	Enterococcus_casseliflavus	NA	0	0	0	0.29379	0	0	0	0	0	0	0	0	0	0	0	0	0	0
Bacteria	Firmicutes	Bacilli	Lactobacillales	Enterococcaceae	Enterococcus	Enterococcus_casseliflavus	Enterococcus_casseliflavus_unclassified	0	0	0	0.29379	0	0	0	0	0	0	0	0	0	0	0	0	0	0
Bacteria	Firmicutes	Bacilli	Lactobacillales	Enterococcaceae	Enterococcus	Enterococcus_cecorum	NA	0	0	0	0.22481	0	0	0	0	0.15661	0	0	0	0	0	0	0	0	0
Bacteria	Firmicutes	Bacilli	Lactobacillales	Enterococcaceae	Enterococcus	Enterococcus_cecorum	Enterococcus_cecorum_unclassified	0	0	0	0.22481	0	0	0	0	0.15661	0	0	0	0	0	0	0	0	0
Bacteria	Firmicutes	Bacilli	Lactobacillales	Enterococcaceae	Enterococcus	Enterococcus_faecalis	NA	0	0.41956	0	0	0	0	0	0	0	0	0	0	0	0	0	0	0	0
Bacteria	Firmicutes	Bacilli	Lactobacillales	Enterococcaceae	Enterococcus	Enterococcus_faecalis	Enterococcus_faecalis_unclassified	0	0.41956	0	0	0	0	0	0	0	0	0	0	0	0	0	0	0	0
Bacteria	Firmicutes	Bacilli	Lactobacillales	Enterococcaceae	Enterococcus	Enterococcus_hirae	NA	0	0	0	2.81815	7.63838	1.14519	0	0	0	0	0	0	0	0.00015	0	0	0	0
Bacteria	Firmicutes	Bacilli	Lactobacillales	Enterococcaceae	Enterococcus	Enterococcus_hirae	Enterococcus_hirae_unclassified	0	0	0	2.81815	7.63838	1.14519	0	0	0	0	0	0	0	0.00015	0	0	0	0
Eukaryota	Microsporidia	Microsporidia_noname	Microsporidia_noname	Enterocytozoonidae	Enterocytozoon	NA	NA	0	0.3076	0	0	0	0	0	0	0	0	0	0	0	0	0	0	0	0
Eukaryota	Microsporidia	Microsporidia_noname	Microsporidia_noname	Enterocytozoonidae	Enterocytozoon	Enterocytozoon_bieneusi	NA	0	0.3076	0	0	0	0	0	0	0	0	0	0	0	0	0	0	0	0
Eukaryota	Microsporidia	Microsporidia_noname	Microsporidia_noname	Enterocytozoonidae	Enterocytozoon	Enterocytozoon_bieneusi	GCA_000209485	0	0.3076	0	0	0	0	0	0	0	0	0	0	0	0	0	0	0	0
Bacteria	Proteobacteria	Gammaproteobacteria	Enterobacteriales	Enterobacteriaceae	Escherichia	NA	NA	0.10334	0.02628	0	16.61839	27.3495	2.16393	0	0	0.15388	0	0	0	0	0.00143	0	0	0	0
Bacteria	Proteobacteria	Gammaproteobacteria	Enterobacteriales	Enterobacteriaceae	Escherichia	Escherichia_coli	NA	0	0	0	11.28618	21.02075	2.07482	0	0	0	0	0	0	0	0.00143	0	0	0	0
Bacteria	Proteobacteria	Gammaproteobacteria	Enterobacteriales	Enterobacteriaceae	Escherichia	Escherichia_coli	Escherichia_coli_unclassified	0	0	0	11.28618	21.02075	2.07482	0	0	0	0	0	0	0	0.00143	0	0	0	0
Bacteria	Proteobacteria	Gammaproteobacteria	Enterobacteriales	Enterobacteriaceae	Escherichia	Escherichia_unclassified	NA	0.10334	0.02628	0	5.33221	6.32875	0.08911	0	0	0.15388	0	0	0	0	0	0	0	0	0
Bacteria	Firmicutes	Bacilli	Bacillales	Bacillales_noname	Exiguobacterium	NA	NA	0	0.06514	0	0	0	0	0	0	0	0	0	0	0	0	0	0	0	0
Bacteria	Firmicutes	Bacilli	Bacillales	Bacillales_noname	Exiguobacterium	Exiguobacterium_sibiricum	NA	0	0.00392	0	0	0	0	0	0	0	0	0	0	0	0	0	0	0	0
Bacteria	Firmicutes	Bacilli	Bacillales	Bacillales_noname	Exiguobacterium	Exiguobacterium_sibiricum	GCF_000019905	0	0.00392	0	0	0	0	0	0	0	0	0	0	0	0	0	0	0	0
Bacteria	Firmicutes	Bacilli	Bacillales	Bacillales_noname	Exiguobacterium	Exiguobacterium_unclassified	NA	0	0.06123	0	0	0	0	0	0	0	0	0	0	0	0	0	0	0	0
Bacteria	Firmicutes	Clostridia	Clostridiales	Ruminococcaceae	Faecalibacterium	NA	NA	0.18852	0.11816	0	0	0.19167	0.13798	0.36031	0.15269	0.69958	0	0	0	0	0	0	0	0	0
Bacteria	Firmicutes	Clostridia	Clostridiales	Ruminococcaceae	Faecalibacterium	Faecalibacterium_prausnitzii	NA	0.18852	0.11816	0	0	0.19167	0.13798	0.36031	0.15269	0.69958	0	0	0	0	0	0	0	0	0
Bacteria	Firmicutes	Clostridia	Clostridiales	Ruminococcaceae	Faecalibacterium	Faecalibacterium_prausnitzii	Faecalibacterium_prausnitzii_unclassified	0.18852	0.11816	0	0	0.19167	0.13798	0.36031	0.15269	0.69958	0	0	0	0	0	0	0	0	0
Viruses	Viruses_noname	Viruses_noname	Viruses_noname	Retroviridae	Gammaretrovirus	NA	NA	0	0.25214	0	0	0	0	0	0	0	0	0	0.15049	0	0	0	0	0	0
Viruses	Viruses_noname	Viruses_noname	Viruses_noname	Retroviridae	Gammaretrovirus	Murine_osteosarcoma_virus	NA	0	0.25214	0	0	0	0	0	0	0	0	0	0.15049	0	0	0	0	0	0
Viruses	Viruses_noname	Viruses_noname	Viruses_noname	Retroviridae	Gammaretrovirus	Murine_osteosarcoma_virus	PRJNA14655	0	0.25214	0	0	0	0	0	0	0	0	0	0.15049	0	0	0	0	0	0
Bacteria	Actinobacteria	Actinobacteria	Actinomycetales	Geodermatophilaceae	Geodermatophilaceae_unclassified	NA	NA	0	0	0	0	0.00688	0	0	0.23304	0	0	0	0	0	0	0	0	0	0
Bacteria	Proteobacteria	Epsilonproteobacteria	Campylobacterales	Helicobacteraceae	Helicobacter	NA	NA	17.52089	23.44612	0	0	0	0	0	0	1.14132	0.01684	0.00288	0	0	0	0	0	0	0.00046
Bacteria	Proteobacteria	Epsilonproteobacteria	Campylobacterales	Helicobacteraceae	Helicobacter	Helicobacter_canadensis	NA	17.52089	23.44612	0	0	0	0	0	0	1.14132	0.01684	0.00288	0	0	0	0	0	0	0.00046
Bacteria	Proteobacteria	Epsilonproteobacteria	Campylobacterales	Helicobacteraceae	Helicobacter	Helicobacter_canadensis	Helicobacter_canadensis_unclassified	17.52089	23.44612	0	0	0	0	0	0	1.14132	0.01684	0.00288	0	0	0	0	0	0	0.00046
Bacteria	Proteobacteria	Alphaproteobacteria	Rhizobiales	Hyphomicrobiaceae	Hyphomicrobiaceae_unclassified	NA	NA	0	0	0	0	0.13437	0	0	0.06881	0	0	0	0	0	0	0	0	0	0
Bacteria	Actinobacteria	Actinobacteria	Actinomycetales	Kineosporiaceae	Kineococcus	NA	NA	0	0	0	0	0	0	0	0.05497	0	0	0	0	0	0	0	0	0	0

Bacteria	Actinobacteria	Actinobacteria	Actinomycetales	Kineosporiaceae	Kineococcus	Kineococcus_radiotolerans	NA	0	0	0	0	0	0	0	0.05497	0	0	0	0	0	0	0	0	0	0
Bacteria	Actinobacteria	Actinobacteria	Actinomycetales	Kineosporiaceae	Kineococcus	Kineococcus_radiotolerans	GCF_000017305	0	0	0	0	0	0	0	0.05497	0	0	0	0	0	0	0	0	0	0
Bacteria	Firmicutes	Clostridia	Clostridiales	Lachnospiraceae	Lachnospiraceae_noname	NA	NA	0.27477	0	0	0	0.10937	0	0	0	0	0	0	0	0	0	0	0	0	0
Bacteria	Firmicutes	Clostridia	Clostridiales	Lachnospiraceae	Lachnospiraceae_noname	Lachnospiraceae_bacterium_8_1_57FAA	NA	0.27477	0	0	0	0.10937	0	0	0	0	0	0	0	0	0	0	0	0	0
Bacteria	Firmicutes	Clostridia	Clostridiales	Lachnospiraceae	Lachnospiraceae_noname	Lachnospiraceae_bacterium_8_1_57FAA	GCF_000185545	0.27477	0	0	0	0.10937	0	0	0	0	0	0	0	0	0	0	0	0	0
Bacteria	Actinobacteria	Actinobacteria	Actinomycetales	Microbacteriaceae	Leifsonia	NA	NA	0	0	0	0.49862	0.13111	0	0	1.24064	0	0	0	0	0	0	0	0	0	0
Bacteria	Actinobacteria	Actinobacteria	Actinomycetales	Microbacteriaceae	Leifsonia	Leifsonia_unclassified	NA	0	0	0	0.49862	0.13111	0	0	1.24064	0	0	0	0	0	0	0	0	0	0
Bacteria	Actinobacteria	Actinobacteria	Actinomycetales	Microbacteriaceae	Leucobacter	NA	NA	0	0	0	0	0	0	0	0.02624	0	0	0	0	0	0	0	0	0	0
Bacteria	Actinobacteria	Actinobacteria	Actinomycetales	Microbacteriaceae	Leucobacter	Leucobacter_unclassified	NA	0	0	0	0	0	0	0	0.02624	0	0	0	0	0	0	0	0	0	0
Bacteria	Firmicutes	Negativicutes	Selenomonadales	Veillonellaceae	Megamonas	NA	NA	6.04103	0.93919	0	0	0.26028	47.0631	5.34088	0.09417	1.16982	0.00144	5.00E-05	0	0	0	27.5882	0.00127	0	0
Bacteria	Firmicutes	Negativicutes	Selenomonadales	Veillonellaceae	Megamonas	Megamonas_funiformis	NA	0.01483	0	0	0	0	0	0.22644	0	0	0	0	0	0	0	0	0	0	0
Bacteria	Firmicutes	Negativicutes	Selenomonadales	Veillonellaceae	Megamonas	Megamonas_funiformis	GCF_000245775	0.01483	0	0	0	0	0	0.22644	0	0	0	0	0	0	0	0	0	0	0
Bacteria	Firmicutes	Negativicutes	Selenomonadales	Veillonellaceae	Megamonas	Megamonas_hypermegale	NA	0.31477	0.10738	0	0	0	8.71704	0.95097	0	0.02284	0	0	0	0	0	1.54602	0	0	0
Bacteria	Firmicutes	Negativicutes	Selenomonadales	Veillonellaceae	Megamonas	Megamonas_hypermegale	GCF_000209975	0.31477	0.10738	0	0	0	8.71704	0.95097	0	0.02284	0	0	0	0	0	1.54602	0	0	0
Bacteria	Firmicutes	Negativicutes	Selenomonadales	Veillonellaceae	Megamonas	Megamonas_rupellensis	NA	5.71143	0.83181	0	0	0.19306	2.50821	4.16348	0.09417	1.14698	0.00144	0	0	0	0	0.66711	0	0	0
Bacteria	Firmicutes	Negativicutes	Selenomonadales	Veillonellaceae	Megamonas	Megamonas_rupellensis	GCF_000378365	5.71143	0.83181	0	0	0.19306	2.50821	4.16348	0.09417	1.14698	0.00144	0	0	0	0	0.66711	0	0	0
Bacteria	Firmicutes	Negativicutes	Selenomonadales	Veillonellaceae	Megamonas	Megamonas_unclassified	NA	0	0	0	0	0.06722	35.83785	0	0	0	0	5.00E-05	0	0	0	25.37508	0.00127	0	0
Bacteria	Firmicutes	Negativicutes	Selenomonadales	Veillonellaceae	Megasphaera	NA	NA	1.66004	1.47737	0	0	0.10305	0.5153	4.28447	0	3.81267	0	0	0	0	0	0.63946	0.04966	0	0.00206
Bacteria	Firmicutes	Negativicutes	Selenomonadales	Veillonellaceae	Megasphaera	Megasphaera_unclassified	NA	1.66004	1.47737	0	0	0.10305	0.5153	4.28447	0	3.81267	0	0	0	0	0	0.63946	0.04966	0	0.00206
Bacteria	Deinococcus_Thermus	Deinococci	Thermales	Thermaceae	Meiothermus	NA	NA	0	0	0	0	0	0	0	0.12236	0	0	0	0	0	0	0	0	0	0
Bacteria	Deinococcus_Thermus	Deinococci	Thermales	Thermaceae	Meiothermus	Meiothermus_silvanus	NA	0	0	0	0	0	0	0	0.12236	0	0	0	0	0	0	0	0	0	0
Bacteria	Deinococcus_Thermus	Deinococci	Thermales	Thermaceae	Meiothermus	Meiothermus_silvanus	GCF_000092125	0	0	0	0	0	0	0	0.12236	0	0	0	0	0	0	0	0	0	0
Bacteria	Proteobacteria	Alphaproteobacteria	Rhizobiales	Phyllobacteriaceae	Mesorhizobium	NA	NA	0	0	0	0	0	0	0	0.03333	0	0	0	0	0	0	0	0	0	0
Bacteria	Proteobacteria	Alphaproteobacteria	Rhizobiales	Phyllobacteriaceae	Mesorhizobium	Mesorhizobium_unclassified	NA	0	0	0	0	0	0	0	0.03333	0	0	0	0	0	0	0	0	0	0
Bacteria	Actinobacteria	Actinobacteria	Actinomycetales	Microbacteriaceae	Microbacterium	NA	NA	0	0.0398	0	0.00881	0	0	0	0	0	0	0	0	0	0	0	0	0	0
Bacteria	Actinobacteria	Actinobacteria	Actinomycetales	Microbacteriaceae	Microbacterium	Microbacterium_laevaniformans	NA	0	0	0	0.00881	0	0	0	0	0	0	0	0	0	0	0	0	0	0
Bacteria	Actinobacteria	Actinobacteria	Actinomycetales	Microbacteriaceae	Microbacterium	Microbacterium_laevaniformans	GCF_000255595	0	0	0	0.00881	0	0	0	0	0	0	0	0	0	0	0	0	0	0
Bacteria	Actinobacteria	Actinobacteria	Actinomycetales	Microbacteriaceae	Microbacterium	Microbacterium_paraoxydans	NA	0	0.0398	0	0	0	0	0	0	0	0	0	0	0	0	0	0	0	0
Bacteria	Actinobacteria	Actinobacteria	Actinomycetales	Microbacteriaceae	Microbacterium	Microbacterium_paraoxydans	GCF_000380465	0	0.0398	0	0	0	0	0	0	0	0	0	0	0	0	0	0	0	0
Bacteria	Deferribacteres	Deferribacteres	Deferribacterales	Deferribacteraceae	Mucispirillum	NA	NA	32.74782	0.86187	0	0	0.78266	0.04654	0	0	0.92847	0.06171	0.00011	0	0	8.00E-05	0.03564	0	0	0.00048
Bacteria	Deferribacteres	Deferribacteres	Deferribacterales	Deferribacteraceae	Mucispirillum	Mucispirillum_schaedleri	NA	32.74782	0.86187	0	0	0.78266	0.04654	0	0	0.92847	0.06171	0.00011	0	0	8.00E-05	0.03564	0	0	0.00048
Bacteria	Deferribacteres	Deferribacteres	Deferribacterales	Deferribacteraceae	Mucispirillum	Mucispirillum_schaedleri	GCF_000487995	32.74782	0.86187	0	0	0.78266	0.04654	0	0	0.92847	0.06171	0.00011	0	0	8.00E-05	0.03564	0	0	0.00048
Bacteria	Tenericutes	Mollicutes	Mycoplasmatales	Mycoplasmataceae	Mycoplasma	NA	NA	0	0	0.07504	0	0	0	0	0	0	0	0	0	0	0	0	0	0	0
Bacteria	Tenericutes	Mollicutes	Mycoplasmatales	Mycoplasmataceae	Mycoplasma	Mycoplasma_anatis	NA	0	0	0.07504	0	0	0	0	0	0	0	0	0	0	0	0	0	0	0
Bacteria	Tenericutes	Mollicutes	Mycoplasmatales	Mycoplasmataceae	Mycoplasma	Mycoplasma_anatis	GCF_000221305	0	0	0.07504	0	0	0	0	0	0	0	0	0	0	0	0	0	0	0
Viruses	Viruses_noname	Viruses_noname	Picornavirales	Secoviridae	Nepovirus	NA	NA	0	0	0	0	0	0	0	0	0	0	0	0	0	0.0073	0.94145	0	0.50147	0
Viruses	Viruses_noname	Viruses_noname	Picornavirales	Secoviridae	Nepovirus	Tobacco_ringspot_virus	NA	0	0	0	0	0	0	0	0	0	0	0	0	0	0.0073	0.94145	0	0.50147	0
Viruses	Viruses_noname	Viruses_noname	Picornavirales	Secoviridae	Nepovirus	Tobacco_ringspot_virus	PRJNA14933	0	0	0	0	0	0	0	0	0	0	0	0	0	0.0073	0.94145	0	0.50147	0
Bacteria	Actinobacteria	Actinobacteria	Actinomycetales	Nocardioidaceae	Nocardioides	NA	NA	0	0.01224	0	0.64013	0	0	0	0.22519	0	0	0	0	0	0	0	0	0	0
Bacteria	Actinobacteria	Actinobacteria	Actinomycetales	Nocardioidaceae	Nocardioides	Nocardioides_unclassified	NA	0	0.01224	0	0.64013	0	0	0	0.22519	0	0	0	0	0	0	0	0	0	0

Bacteria	Actinobacteria	Actinobacteria	Coriobacteriales	Coriobacteriaceae	Olsenella	NA	NA	0.42827	0.25282	0	0	0.17772	0.85643	1.3765	0.43828	1.77735	0.00172	0.0001	0	0	2.00E-05	0.59723	0.02389	0	0.00026
Bacteria	Actinobacteria	Actinobacteria	Coriobacteriales	Coriobacteriaceae	Olsenella	Olsenella_unclassified	NA	0.42827	0.25282	0	0	0.17772	0.85643	1.3765	0.43828	1.77735	0.00172	0.0001	0	0	2.00E-05	0.59723	0.02389	0	0.00026
Viruses	Viruses_noname	Viruses_noname	Viruses_noname	Hepadnaviridae	Orthohepadnavirus	NA	NA	0	39.00609	0	0	0	0	0	0	0	0	0	1.83806	0	0	0	0	0	0
Viruses	Viruses_noname	Viruses_noname	Viruses_noname	Hepadnaviridae	Orthohepadnavirus	Orthohepadnavirus_unclassified	NA	0	39.00609	0	0	0	0	0	0	0	0	0	1.83806	0	0	0	0	0	0
Bacteria	Firmicutes	Clostridia	Clostridiales	Oscillospiraceae	Oscillibacter	NA	NA	0.61178	0.13764	0	0	0.22485	0.20308	0.16294	0.184	0.35551	0	0	0	0	0	0	0	0	0
Bacteria	Firmicutes	Clostridia	Clostridiales	Oscillospiraceae	Oscillibacter	Oscillibacter_unclassified	NA	0.61178	0.13764	0	0	0.22485	0.20308	0.16294	0.184	0.35551	0	0	0	0	0	0	0	0	0
Bacteria	Proteobacteria	Gammaproteobacteria	Enterobacteriales	Enterobacteriaceae	Pantoea	NA	NA	0	0	0	0	0.06756	0	0	0.06409	0	0	0	0	0	0	0	0	0	0
Bacteria	Proteobacteria	Gammaproteobacteria	Enterobacteriales	Enterobacteriaceae	Pantoea	Pantoea_agglomerans	NA	0	0	0	0	0	0	0	0.02086	0	0	0	0	0	0	0	0	0	0
Bacteria	Proteobacteria	Gammaproteobacteria	Enterobacteriales	Enterobacteriaceae	Pantoea	Pantoea_agglomerans	Pantoea_agglomerans_unclassified	0	0	0	0	0	0	0	0.02086	0	0	0	0	0	0	0	0	0	0
Bacteria	Proteobacteria	Gammaproteobacteria	Enterobacteriales	Enterobacteriaceae	Pantoea	Pantoea_unclassified	NA	0	0	0	0	0.06756	0	0	0.04323	0	0	0	0	0	0	0	0	0	0
Bacteria	Bacteroidetes	Bacteroidia	Bacteroidales	Porphyromonadaceae	Parabacteroides	NA	NA	0.12069	0	0	0	0.62065	0	0	0	0	0.0013	0	0	0	0.0005	0	0	0	0
Bacteria	Bacteroidetes	Bacteroidia	Bacteroidales	Porphyromonadaceae	Parabacteroides	Parabacteroides_unclassified	NA	0.12069	0	0	0	0.62065	0	0	0	0	0.0013	0	0	0	0.0005	0	0	0	0
Bacteria	Actinobacteria	Actinobacteria	Solirubrobacterales	Patulibacteraceae	Patulibacter	NA	NA	0	0	0	0.01263	0	0	0	0	0	0	0	0	0	0	0	0	0	0
Bacteria	Actinobacteria	Actinobacteria	Solirubrobacterales	Patulibacteraceae	Patulibacter	Patulibacter_americanus	NA	0	0	0	0.01263	0	0	0	0	0	0	0	0	0	0	0	0	0	0
Bacteria	Actinobacteria	Actinobacteria	Solirubrobacterales	Patulibacteraceae	Patulibacter	Patulibacter_americanus	GCF_000420025	0	0	0	0.01263	0	0	0	0	0	0	0	0	0	0	0	0	0	0
Bacteria	Firmicutes	Clostridia	Clostridiales	Peptostreptococcaceae	Peptostreptococcaceae_noname	NA	NA	0.31465	0.40003	88.20273	0.25685	4.25478	0	0.47515	0	0	0.14697	0.01878	96.77642	10.99065	0.22925	5.28887	0	0	0.05322
Bacteria	Firmicutes	Clostridia	Clostridiales	Peptostreptococcaceae	Peptostreptococcaceae_noname	Clostridium_glycolicum	NA	0	0	2.35869	0	0	0	0	0	0	0	0	0.01706	0	0	0	0	0	0
Bacteria	Firmicutes	Clostridia	Clostridiales	Peptostreptococcaceae	Peptostreptococcaceae_noname	Clostridium_glycolicum	GCF_000373865	0	0	2.35869	0	0	0	0	0	0	0	0	0.01706	0	0	0	0	0	0
Bacteria	Firmicutes	Clostridia	Clostridiales	Peptostreptococcaceae	Peptostreptococcaceae_noname	Peptostreptococcaceae_noname_unclassified	NA	0.31465	0.40003	85.84403	0.25685	4.25478	0	0.47515	0	0	0.14697	0.01878	96.75936	10.99065	0.22925	5.28887	0	0	0.05322
Viruses	Viruses_noname	Viruses_noname	Viruses_noname	Luteoviridae	Polerovirus	NA	NA	0	0	0	0	0	0	0	0	0	0.02275	0.04128	0	0	0.00114	0	0.70135	0	0.01167
Viruses	Viruses_noname	Viruses_noname	Viruses_noname	Luteoviridae	Polerovirus	Cereal_yellow_dwarf_virus_RPV	NA	0	0	0	0	0	0	0	0	0	0	0.01856	0	0	0	0	0	0	0
Viruses	Viruses_noname	Viruses_noname	Viruses_noname	Luteoviridae	Polerovirus	Cereal_yellow_dwarf_virus_RPV	PRJNA14883	0	0	0	0	0	0	0	0	0	0	0.01856	0	0	0	0	0	0	0
Viruses	Viruses_noname	Viruses_noname	Viruses_noname	Luteoviridae	Polerovirus	Chickpea_chlorotic_stunt_virus	NA	0	0	0	0	0	0	0	0	0	0.02275	0.02272	0	0	0.00114	0	0.70135	0	0.01167
Viruses	Viruses_noname	Viruses_noname	Viruses_noname	Luteoviridae	Polerovirus	Chickpea_chlorotic_stunt_virus	PRJNA17363	0	0	0	0	0	0	0	0	0	0.02275	0.02272	0	0	0.00114	0	0.70135	0	0.01167
Viruses	Viruses_noname	Viruses_noname	Tymovirales	Alphaflexiviridae	Potexvirus	NA	NA	0	0	0.41221	0	0	0	0	0	0	15.38324	2.65495	0	0	0.92995	0	35.70817	1.8846	89.40646
Viruses	Viruses_noname	Viruses_noname	Tymovirales	Alphaflexiviridae	Potexvirus	Alternanthera_mosaic_virus	NA	0	0	0.41221	0	0	0	0	0	0	8.90958	0.04526	0	0	0	0	4.70615	1.8846	88.0256
Viruses	Viruses_noname	Viruses_noname	Tymovirales	Alphaflexiviridae	Potexvirus	Alternanthera_mosaic_virus	PRJNA16333	0	0	0.41221	0	0	0	0	0	0	8.90958	0.04526	0	0	0	0	4.70615	1.8846	88.0256
Viruses	Viruses_noname	Viruses_noname	Tymovirales	Alphaflexiviridae	Potexvirus	White_clover_mosaic_virus	NA	0	0	0	0	0	0	0	0	0	6.47365	2.60969	0	0	0.92995	0	31.00202	0	1.38086
Viruses	Viruses_noname	Viruses_noname	Tymovirales	Alphaflexiviridae	Potexvirus	White_clover_mosaic_virus	PRJNA15069	0	0	0	0	0	0	0	0	0	6.47365	2.60969	0	0	0.92995	0	31.00202	0	1.38086
Bacteria	Actinobacteria	Actinobacteria	Actinomycetales	Propionibacteriaceae	Propionibacteriaceae_unclassified	NA	NA	0	0	0	0.41635	0	0	0	0.16088	0	0	0	0	14.85175	0	0.47646	0.09626	0.00027	0.001
Bacteria	Actinobacteria	Actinobacteria	Actinomycetales	Propionibacteriaceae	Propionibacterium	NA	NA	0	0	0	0.2778	0.00577	0	0	1.24126	0	0	0	0	0	0	0	0	0	0
Bacteria	Actinobacteria	Actinobacteria	Actinomycetales	Propionibacteriaceae	Propionibacterium	Propionibacterium_acnes	NA	0	0	0	0.2778	0.00577	0	0	1.22089	0	0	0	0	0	0	0	0	0	0
Bacteria	Actinobacteria	Actinobacteria	Actinomycetales	Propionibacteriaceae	Propionibacterium	Propionibacterium_acnes	Propionibacterium_acnes_unclassified	0	0	0	0.2778	0.00577	0	0	1.22089	0	0	0	0	0	0	0	0	0	0
Bacteria	Actinobacteria	Actinobacteria	Actinomycetales	Propionibacteriaceae	Propionibacterium	Propionibacterium_granulosum	NA	0	0	0	0	0	0	0	0.02037	0	0	0	0	0	0	0	0	0	0

Bacteria	Actinobacteria	Actinobacteria	Actinomycetales	Propionibacteriaceae	Propionibacterium	Propionibacterium_granulosum	Propionibacterium_granulosum_unclassified	0	0	0	0	0	0	0	0.02037	0	0	0	0	0	0	0	0	0	0
Bacteria	Firmicutes	Clostridia	Clostridiales	Clostridiales_noname	Pseudoflavonifractor	NA	NA	0.43977	0.09997	0	0	0.29822	0.1329	0.09017	0.27583	0.42259	0	0	0	0	0	0	0	0	0
Bacteria	Firmicutes	Clostridia	Clostridiales	Clostridiales_noname	Pseudoflavonifractor	Pseudoflavonifractor_capillosus	NA	0.43977	0.09997	0	0	0.29822	0.1329	0.09017	0.27583	0.42259	0	0	0	0	0	0	0	0	0
Bacteria	Firmicutes	Clostridia	Clostridiales	Clostridiales_noname	Pseudoflavonifractor	Pseudoflavonifractor_capillosus	GCF_000169255	0.43977	0.09997	0	0	0.29822	0.1329	0.09017	0.27583	0.42259	0	0	0	0	0	0	0	0	0
Bacteria	Actinobacteria	Actinobacteria	Actinomycetales	Nocardiaceae	Rhodococcus	NA	NA	0	0	0	0.20261	0	0	0	0	0	0	0	0	0	0	0	0	0	0
Bacteria	Actinobacteria	Actinobacteria	Actinomycetales	Nocardiaceae	Rhodococcus	Rhodococcus_erythropolis	NA	0	0	0	0.20261	0	0	0	0	0	0	0	0	0	0	0	0	0	0
Bacteria	Actinobacteria	Actinobacteria	Actinomycetales	Nocardiaceae	Rhodococcus	Rhodococcus_erythropolis	Rhodococcus_erythropolis_unclassified	0	0	0	0.20261	0	0	0	0	0	0	0	0	0	0	0	0	0	0
Bacteria	Proteobacteria	Alphaproteobacteria	Rhizobiales	Bradyrhizobiaceae	Rhodopseudomonas	NA	NA	0	0	0	0	0	0	0	0.05236	0	0	0	0	0	0	0	0	0	0
Bacteria	Proteobacteria	Alphaproteobacteria	Rhizobiales	Bradyrhizobiaceae	Rhodopseudomonas	Rhodopseudomonas_unclassified	NA	0	0	0	0	0	0	0	0.05236	0	0	0	0	0	0	0	0	0	0
Bacteria	Firmicutes	Clostridia	Clostridiales	Ruminococcaceae	Ruminococcaceae_noname	NA	NA	1.94723	0.78503	0	0	1.1444	0.7286	4.54075	2.37086	3.39976	0	0	0	0	0	0	0	0	0
Bacteria	Firmicutes	Clostridia	Clostridiales	Ruminococcaceae	Ruminococcaceae_noname	Ruminococcaceae_bacterium_D16	NA	1.94723	0.78503	0	0	1.1444	0.7286	4.54075	2.37086	3.39976	0	0	0	0	0	0	0	0	0
Bacteria	Firmicutes	Clostridia	Clostridiales	Ruminococcaceae	Ruminococcaceae_noname	Ruminococcaceae_bacterium_D16	GCF_000177015	1.94723	0.78503	0	0	1.1444	0.7286	4.54075	2.37086	3.39976	0	0	0	0	0	0	0	0	0
Bacteria	Actinobacteria	Actinobacteria	Actinomycetales	Sanguibacteraceae	Sanguibacter	NA	NA	0	0	0	0.06648	0.0358	0	0	0	0	0	0	0	0	0	0	0	0	0
Bacteria	Actinobacteria	Actinobacteria	Actinomycetales	Sanguibacteraceae	Sanguibacter	Sanguibacter_keddieii	NA	0	0	0	0.06648	0.0358	0	0	0	0	0	0	0	0	0	0	0	0	0
Bacteria	Actinobacteria	Actinobacteria	Actinomycetales	Sanguibacteraceae	Sanguibacter	Sanguibacter_keddieii	GCF_000024925	0	0	0	0.06648	0.0358	0	0	0	0	0	0	0	0	0	0	0	0	0
Viruses	Viruses_noname	Viruses_noname	Picornavirales	Picornaviridae	Sapelovirus	NA	NA	0	0	0	0	0	0	0	0	0	0	0	0	0	7.00E-05	0	0	0	0
Viruses	Viruses_noname	Viruses_noname	Picornavirales	Picornaviridae	Sapelovirus	Avian_sapelovirus	NA	0	0	0	0	0	0	0	0	0	0	0	0	0	7.00E-05	0	0	0	0
Viruses	Viruses_noname	Viruses_noname	Picornavirales	Picornaviridae	Sapelovirus	Avian_sapelovirus	PRJNA15039	0	0	0	0	0	0	0	0	0	0	0	0	0	7.00E-05	0	0	0	0
Bacteria	Actinobacteria	Actinobacteria	Bifidobacteriales	Bifidobacteriaceae	Scardovia	NA	NA	0	0	0	0	0	0	0.13085	0	0	0	0	0	0	0	0	0	0	0
Bacteria	Actinobacteria	Actinobacteria	Bifidobacteriales	Bifidobacteriaceae	Scardovia	Scardovia_unclassified	NA	0	0	0	0	0	0	0.13085	0	0	0	0	0	0	0	0	0	0	0
Viruses	Viruses_noname	Viruses_noname	Caudovirales	Siphoviridae	Siphoviridae_noname	NA	NA	0	0	0	0	0	0	0	1.60394	1.96681	0	0	0	0	0	0	0	0	0
Viruses	Viruses_noname	Viruses_noname	Caudovirales	Siphoviridae	Siphoviridae_noname	Propionibacterium_phage_P14_4	NA	0	0	0	0	0	0	0	1.60394	0	0	0	0	0	0	0	0	0	0
Viruses	Viruses_noname	Viruses_noname	Caudovirales	Siphoviridae	Siphoviridae_noname	Propionibacterium_phage_P14_4	PRJNA177530	0	0	0	0	0	0	0	1.60394	0	0	0	0	0	0	0	0	0	0
Viruses	Viruses_noname	Viruses_noname	Caudovirales	Siphoviridae	Siphoviridae_noname	Propionibacterium_phage_PHL060L00	NA	0	0	0	0	0	0	0	0	1.96681	0	0	0	0	0	0	0	0	0
Viruses	Viruses_noname	Viruses_noname	Caudovirales	Siphoviridae	Siphoviridae_noname	Propionibacterium_phage_PHL060L00	PRJNA219122	0	0	0	0	0	0	0	0	1.96681	0	0	0	0	0	0	0	0	0
Viruses	Viruses_noname	Viruses_noname	Viruses_noname	Viruses_noname	Sobemovirus	NA	NA	0	0	0	0	0	0	0	0	0	0.24793	0.04128	0	0	0	0	0	0	0.23828
Viruses	Viruses_noname	Viruses_noname	Viruses_noname	Viruses_noname	Sobemovirus	Cocksfoot_mottle_virus	NA	0	0	0	0	0	0	0	0	0	0	0.04128	0	0	0	0	0	0	0
Viruses	Viruses_noname	Viruses_noname	Viruses_noname	Viruses_noname	Sobemovirus	Cocksfoot_mottle_virus	PRJNA15078	0	0	0	0	0	0	0	0	0	0	0.04128	0	0	0	0	0	0	0
Viruses	Viruses_noname	Viruses_noname	Viruses_noname	Viruses_noname	Sobemovirus	Subterranean_clover_mottle_virus	NA	0	0	0	0	0	0	0	0	0	0.24793	0	0	0	0	0	0	0	0.23828
Viruses	Viruses_noname	Viruses_noname	Viruses_noname	Viruses_noname	Sobemovirus	Subterranean_clover_mottle_virus	PRJNA15403	0	0	0	0	0	0	0	0	0	0.24793	0	0	0	0	0	0	0	0.23828
Bacteria	Firmicutes	Erysipelotrichia	Erysipelotrichales	Erysipelotrichaceae	Solobacterium	NA	NA	0	0	0	0.026	0	0	0	0	0	0	0	0	0	0	0	0	0	0
Bacteria	Firmicutes	Erysipelotrichia	Erysipelotrichales	Erysipelotrichaceae	Solobacterium	Solobacterium_moorei	NA	0	0	0	0.026	0	0	0	0	0	0	0	0	0	0	0	0	0	0
Bacteria	Firmicutes	Erysipelotrichia	Erysipelotrichales	Erysipelotrichaceae	Solobacterium	Solobacterium_moorei	GCF_000186945	0	0	0	0.026	0	0	0	0	0	0	0	0	0	0	0	0	0	0
Bacteria	Proteobacteria	Alphaproteobacteria	Sphingomonadales	Sphingomonadaceae	Sphingomonas	NA	NA	0	0	0	0	0	0	0	0	0	0	0.00149	0.09387	6.00394	0.00099	1.85942	0.0698	0.00085	0.00114
Bacteria	Proteobacteria	Alphaproteobacteria	Sphingomonadales	Sphingomonadaceae	Sphingomonas	Sphingomonas_echinoides	NA	0	0	0	0	0	0	0	0	0	0	0.00149	0.09387	6.00394	0.00099	1.85942	0.0698	0.00085	0.00114
Bacteria	Proteobacteria	Alphaproteobacteria	Sphingomonadales	Sphingomonadaceae	Sphingomonas	Sphingomonas_echinoides	GCF_000241465	0	0	0	0	0	0	0	0	0	0	0.00149	0.09387	6.00394	0.00099	1.85942	0.0698	0.00085	0.00114

Bacteria	Firmicutes	Bacilli	Lactobacillales	Streptococcaceae	Streptococcus	NA	NA	0	0	0	0.09849	0	0	0	0	0	0	0	0	0	0	0	0	0	0
Bacteria	Firmicutes	Bacilli	Lactobacillales	Streptococcaceae	Streptococcus	Streptococcus_suis	NA	0	0	0	0.09849	0	0	0	0	0	0	0	0	0	0	0	0	0	0
Bacteria	Firmicutes	Bacilli	Lactobacillales	Streptococcaceae	Streptococcus	Streptococcus_suis	Streptococcus_suis_unclassified	0	0	0	0.09849	0	0	0	0	0	0	0	0	0	0	0	0	0	0
Bacteria	Firmicutes	Clostridia	Clostridiales	Ruminococcaceae	Subdoligranulum	NA	NA	23.27921	11.60023	0	0	20.62398	26.55732	55.20846	24.55052	57.11315	0.0139	0.00356	0	0	0.00051	1.57571	0.0283	0.0001	0.0033
Bacteria	Firmicutes	Clostridia	Clostridiales	Ruminococcaceae	Subdoligranulum	Subdoligranulum_unclassified	NA	17.85137	8.86103	0	0	16.53054	14.78682	40.35159	19.82959	43.7236	0.0139	0.00353	0	0	0.0005	1.30374	0.0283	0.0001	0.0033
Bacteria	Firmicutes	Clostridia	Clostridiales	Ruminococcaceae	Subdoligranulum	Subdoligranulum_variabile	NA	5.42783	2.7392	0	0	4.09344	11.77051	14.85686	4.72093	13.38955	0	3.00E-05	0	0	1.00E-05	0.27196	0	0	0
Bacteria	Firmicutes	Clostridia	Clostridiales	Ruminococcaceae	Subdoligranulum	Subdoligranulum_variabile	GCF_000157955	5.42783	2.7392	0	0	4.09344	11.77051	14.85686	4.72093	13.38955	0	3.00E-05	0	0	1.00E-05	0.27196	0	0	0
Bacteria	Proteobacteria	Betaproteobacteria	Burkholderiales	Sutterellaceae	Sutterellaceae_unclassified	NA	NA	1.99192	0.34971	0	0	1.83764	0.70754	3.00672	1.04329	1.59449	0.07948	0.01018	0	0	0.00186	7.7962	0.62743	0.00131	0.00548
Viruses	Viruses_noname	Viruses_noname	Viruses_noname	Virgaviridae	Tobamovirus	NA	NA	0	0	0	0	0.05243	0	0.2459	0	0	0	0	0	0	0	14.2738	0.43941	0	0
Viruses	Viruses_noname	Viruses_noname	Viruses_noname	Virgaviridae	Tobamovirus	Tobacco_mosaic_virus	NA	0	0	0	0	0	0	0	0	0	0	0	0	0	0	14.2738	0.43941	0	0
Viruses	Viruses_noname	Viruses_noname	Viruses_noname	Virgaviridae	Tobamovirus	Tobacco_mosaic_virus	PRJNA15071	0	0	0	0	0	0	0	0	0	0	0	0	0	0	14.2738	0.43941	0	0
Viruses	Viruses_noname	Viruses_noname	Viruses_noname	Virgaviridae	Tobamovirus	Youcai_mosaic_virus	NA	0	0	0	0	0.05243	0	0.2459	0	0	0	0	0	0	0	0	0	0	0
Viruses	Viruses_noname	Viruses_noname	Viruses_noname	Virgaviridae	Tobamovirus	Youcai_mosaic_virus	PRJNA14869	0	0	0	0	0.05243	0	0.2459	0	0	0	0	0	0	0	0	0	0	0
Bacteria	Firmicutes	Erysipelotrichia	Erysipelotrichales	Erysipelotrichaceae	Turicibacter	NA	NA	0	0	8.81827	0	0	0	0	0	0	0	0	0.28563	0	0	0	0	0	0
Bacteria	Firmicutes	Erysipelotrichia	Erysipelotrichales	Erysipelotrichaceae	Turicibacter	Turicibacter_sanguinis	NA	0	0	4.20121	0	0	0	0	0	0	0	0	0.03175	0	0	0	0	0	0
Bacteria	Firmicutes	Erysipelotrichia	Erysipelotrichales	Erysipelotrichaceae	Turicibacter	Turicibacter_sanguinis	GCF_000178255	0	0	4.20121	0	0	0	0	0	0	0	0	0.03175	0	0	0	0	0	0
Bacteria	Firmicutes	Erysipelotrichia	Erysipelotrichales	Erysipelotrichaceae	Turicibacter	Turicibacter_unclassified	NA	0	0	4.61706	0	0	0	0	0	0	0	0	0.25388	0	0	0	0	0	0
Viruses	Viruses_noname	Viruses_noname	Tymovirales	Tymoviridae	Tymovirus	NA	NA	0	0	0	0	0	0	0	0	0	0.23753	0.20474	0	0	0.00135	0	0	0	0.3249
Viruses	Viruses_noname	Viruses_noname	Tymovirales	Tymoviridae	Tymovirus	Plantago_mottle_virus	NA	0	0	0	0	0	0	0	0	0	0.23753	0.20474	0	0	0.00135	0	0	0	0.3249
Viruses	Viruses_noname	Viruses_noname	Tymovirales	Tymoviridae	Tymovirus	Plantago_mottle_virus	PRJNA32683	0	0	0	0	0	0	0	0	0	0.23753	0.20474	0	0	0.00135	0	0	0	0.3249
Viruses	Viruses_noname	Viruses_noname	Viruses_noname	Viruses_noname	Viruses_noname	NA	NA	0.72991	0.27839	0	0	0.99204	0	1.65336	0.84246	1.08685	0.38578	9.79959	0	0	0.01494	0	8.70636	97.55168	0.04277
Viruses	Viruses_noname	Viruses_noname	Viruses_noname	Viruses_noname	Viruses_noname	Acyrthosiphon_pisum_virus	NA	0	0	0	0	0	0	0	0	0	0.01634	0	0	0	0	0	0	0	0
Viruses	Viruses_noname	Viruses_noname	Viruses_noname	Viruses_noname	Viruses_noname	Acyrthosiphon_pisum_virus	PRJNA40357	0	0	0	0	0	0	0	0	0	0.01634	0	0	0	0	0	0	0	0
Viruses	Viruses_noname	Viruses_noname	Viruses_noname	Viruses_noname	Viruses_noname	Peanut_stunt_virus_satellite_RNA	NA	0	0	0	0	0.63953	0	0	0	0	0.04418	9.54584	0	0	0	0	0.17987	0.00509	0.00933
Viruses	Viruses_noname	Viruses_noname	Viruses_noname	Viruses_noname	Viruses_noname	Peanut_stunt_virus_satellite_RNA	PRJNA14502	0	0	0	0	0.63953	0	0	0	0	0.04418	9.54584	0	0	0	0	0.17987	0.00509	0.00933
Viruses	Viruses_noname	Viruses_noname	Viruses_noname	Viruses_noname	Viruses_noname	Sclerophthora_macrospora_virus_A	NA	0.72991	0.27839	0	0	0.3525	0	1.65336	0.84246	1.08685	0.25164	0.25276	0	0	0.00142	0	8.52649	97.54598	0.03343
Viruses	Viruses_noname	Viruses_noname	Viruses_noname	Viruses_noname	Viruses_noname	Sclerophthora_macrospora_virus_A	PRJNA14361	0.72991	0.27839	0	0	0.3525	0	1.65336	0.84246	1.08685	0.25164	0.25276	0	0	0.00142	0	8.52649	97.54598	0.03343
Viruses	Viruses_noname	Viruses_noname	Viruses_noname	Viruses_noname	Viruses_noname	Sclerophthora_macrospora_virus_B	NA	0	0	0	0	0	0	0	0	0	0.07362	0.00099	0	0	0.01352	0	0	0.00061	0
Viruses	Viruses_noname	Viruses_noname	Viruses_noname	Viruses_noname	Viruses_noname	Sclerophthora_macrospora_virus_B	PRJNA14912	0	0	0	0	0	0	0	0	0	0.07362	0.00099	0	0	0.01352	0	0	0.00061	0
Bacteria	Proteobacteria	Gammaproteobacteria	Xanthomonadales	Xanthomonadaceae	Xanthomonas	NA	NA	0	0	0	0.14104	0	0	0	0	0	0	0	0	0	0	0	0	0	0
Bacteria	Proteobacteria	Gammaproteobacteria	Xanthomonadales	Xanthomonadaceae	Xanthomonas	Xanthomonas_translucens	NA	0	0	0	0.14104	0	0	0	0	0	0	0	0	0	0	0	0	0	0
Bacteria	Proteobacteria	Gammaproteobacteria	Xanthomonadales	Xanthomonadaceae	Xanthomonas	Xanthomonas_translucens	Xanthomonas_translucens_unclassified	0	0	0	0.14104	0	0	0	0	0	0	0	0	0	0	0	0	0	0
Viruses	Viruses_noname	Viruses_noname	Viruses_noname	Adenoviridae	NA	NA	NA	0	1.59919	0	0	0	0	0	0	0	0	0	0	0	0	0	0	0	0
Viruses	Viruses_noname	Viruses_noname	Tymovirales	Alphaflexiviridae	NA	NA	NA	0	0	0.41221	0	0	0	0	0	0	15.38324	2.65495	0	0	0.92995	0	35.70817	1.8846	89.40646
Bacteria	Firmicutes	Bacilli	Bacillales	Bacillaceae	NA	NA	NA	0	0	0	0	0	0	0	0.00542	0	0	0	0	0	0	0	0	0	0
Bacteria	Firmicutes	Bacilli	Bacillales	Bacillales_noname	NA	NA	NA	0	0.06514	0	0	0	0	0	0	0	0	0	0	0	0	0	0	0	0
Bacteria	Bacteroidetes	Bacteroidia	Bacteroidales	Bacteroidaceae	NA	NA	NA	1.45591	0.17999	0	0	0.15878	0.64833	2.95965	0.07751	0.32785	0	0	0	0	0	0.007	0	0	0
Viruses	Viruses_noname	Viruses_noname	Tymovirales	Betaflexiviridae	NA	NA	NA	0	0	0	0	0	0	0	0	0	2.61901	57.46443	0	0	0.15135	0	0	0	0.01435
Bacteria	Actinobacteria	Actinobacteria	Bifidobacteriales	Bifidobacteriaceae	NA	NA	NA	0	0	0	0	0	0	0.13085	0	0	0	0	0	0	0	0	0	0	0
Bacteria	Spirochaetes	Spirochaetia	Spirochaetales	Brachyspiraceae	NA	NA	NA	0	0	0	0	0	2.05348	0	0	0	0	0	0	0	0	4.78845	0	0	0

Bacteria	Proteobacteria	Alphaproteobacteria	Rhizobiales	Bradyrhizobiaceae	NA	NA	NA	0	0	0	0	0	0	0	0.05236	0	0	0	0	0	0	0	0	0	0
Viruses	Viruses_noname	Viruses_noname	Viruses_noname	Bromoviridae	NA	NA	NA	1.82685	0.66775	1.24905	1.02524	1.39692	0.98447	3.10358	1.05428	3.06025	80.56977	29.68453	0.84549	35.89444	98.65139	9.07001	52.42827	0.05426	9.88436
Bacteria	Proteobacteria	Betaproteobacteria	Burkholderiales	Burkholderiaceae	NA	NA	NA	0	0	0	0	0	0	0	0.10807	0	0	0	0	0	0	0	0	0	0
Bacteria	Proteobacteria	Epsilonproteobacteria	Campylobacterales	Campylobacteraceae	NA	NA	NA	0	1.0137	0	0	2.12769	0	0	0	0	0	0	0	0	0.00027	0	0	0	0
Bacteria	Actinobacteria	Actinobacteria	Actinomycetales	Cellulomonadaceae	NA	NA	NA	0	0.01137	0	0	0	0	0	0.18923	0	0	0	0	0	0	0	0	0	0
Bacteria	Cyanobacteria	Cyanobacteria_noname	Chroococcales	Chroococcales_noname	NA	NA	NA	0	0	0.34458	0	0	0	0	0	0	0	0	0	0	0	0	0	0	0
Bacteria	Firmicutes	Clostridia	Clostridiales	Clostridiaceae	NA	NA	NA	1.09409	0.29494	0.58137	0	0.25414	0.27687	1.588	0.79154	1.25977	0	0	0.00224	0	0	0	0	0	0
Bacteria	Firmicutes	Clostridia	Clostridiales	Clostridiales_noname	NA	NA	NA	0.43977	0.09997	0	0	0.29822	0.1329	0.09017	0.27583	0.42259	0	0	0	0	0	0	0	0	0
Bacteria	Proteobacteria	Betaproteobacteria	Burkholderiales	Comamonadaceae	NA	NA	NA	0	0	0	0	0	0	0	0	0	0	0	0	1.6556	0	0	0	0	0
Bacteria	Actinobacteria	Actinobacteria	Coriobacteriales	Coriobacteriaceae	NA	NA	NA	1.32056	1.09966	0	0	1.87708	4.22688	3.86159	1.16361	5.47197	0.01662	0.00095	0	0	0.00096	4.4557	0.06619	2.00E-05	0.00234
Bacteria	Deferribacteres	Deferribacteres	Deferribacterales	Deferribacteraceae	NA	NA	NA	32.74782	0.86187	0	0	0.78266	0.04654	0	0	0.92847	0.06171	0.00011	0	0	8.00E-05	0.03564	0	0	0.00048
Bacteria	Proteobacteria	Deltaproteobacteria	Desulfovibrionales	Desulfovibrionaceae	NA	NA	NA	0	0	0	0	0.09051	0.16372	0	0	0.1324	0	0	0	0	3.00E-05	0	0	0	0
Bacteria	Proteobacteria	Gammaproteobacteria	Enterobacteriales	Enterobacteriaceae	NA	NA	NA	0.10334	0.02628	0	16.61839	27.41706	2.16393	0	0.06409	0.15388	0	0	0	0	0.00143	0	0	0	0
Bacteria	Firmicutes	Bacilli	Lactobacillales	Enterococcaceae	NA	NA	NA	0	0.41956	0	3.33674	7.63838	1.14519	0	0	0.15661	0	0	0	0	0.00015	0	0	0	0
Eukaryota	Microsporidia	Microsporidia_noname	Microsporidia_noname	Enterocytozoonidae	NA	NA	NA	0	0.3076	0	0	0	0	0	0	0	0	0	0	0	0	0	0	0	0
Bacteria	Firmicutes	Erysipelotrichia	Erysipelotrichales	Erysipelotrichaceae	NA	NA	NA	0	0	8.81827	0.026	0	0	0	0	0	0	0	0.28563	0	0	0	0	0	0
Bacteria	Actinobacteria	Actinobacteria	Actinomycetales	Geodermatophilaceae	NA	NA	NA	0	0	0	0	0.00688	0	0	0.23304	0	0	0	0	0	0	0	0	0	0
Bacteria	Proteobacteria	Epsilonproteobacteria	Campylobacterales	Helicobacteraceae	NA	NA	NA	17.52089	23.44612	0	0	0	0	0	0	1.14132	0.01684	0.00288	0	0	0	0	0	0	0.00046
Viruses	Viruses_noname	Viruses_noname	Viruses_noname	Hepadnaviridae	NA	NA	NA	0	39.00609	0	0	0	0	0	0	0	0	0	1.83806	0	0	0	0	0	0
Bacteria	Proteobacteria	Alphaproteobacteria	Rhizobiales	Hyphomicrobiaceae	NA	NA	NA	0	0	0	0	0.13437	0	0	0.06881	0	0	0	0	0	0	0	0	0	0
Bacteria	Actinobacteria	Actinobacteria	Actinomycetales	Kineosporiaceae	NA	NA	NA	0	0	0	0	0	0	0	0.05497	0	0	0	0	0	0	0	0	0	0
Bacteria	Firmicutes	Clostridia	Clostridiales	Lachnospiraceae	NA	NA	NA	3.56859	1.80391	0	0	3.14821	5.61761	11.09589	1.6711	6.03641	0.18597	0.00716	0	0	0.00494	20.44276	0.84893	0.00195	0.00774
Viruses	Viruses_noname	Viruses_noname	Viruses_noname	Luteoviridae	NA	NA	NA	0	0	0	0	0	0	0	0	0	0.02275	0.04128	0	0	0.00114	0	0.70135	0	0.01167
Bacteria	Actinobacteria	Actinobacteria	Actinomycetales	Microbacteriaceae	NA	NA	NA	0.70716	11.89468	0	70.97016	16.6451	3.21164	1.76008	52.13529	3.76136	0	0	0	0	9.00E-05	0	0	5.00E-05	0
Bacteria	Actinobacteria	Actinobacteria	Actinomycetales	Micromonosporaceae	NA	NA	NA	0	0	0	0	0	0	0	0.11787	0	0	0	0	0	0	0	0	0	0
Bacteria	Tenericutes	Mollicutes	Mycoplasmatales	Mycoplasmataceae	NA	NA	NA	0	0	0.07504	0	0	0	0	0	0	0	0	0	0	0	0	0	0	0
Bacteria	Actinobacteria	Actinobacteria	Actinomycetales	Nocardiaceae	NA	NA	NA	0	0	0	0.20261	0	0	0	0	0	0	0	0	0	0	0	0	0	0
Bacteria	Actinobacteria	Actinobacteria	Actinomycetales	Nocardioidaceae	NA	NA	NA	0	0.01224	0	0.64013	0	0	0	0.22519	0	0	0	0	0	0	0	0	0	0
Bacteria	Firmicutes	Clostridia	Clostridiales	Oscillospiraceae	NA	NA	NA	0.61178	0.13764	0	0	0.22485	0.20308	0.16294	0.184	0.35551	0	0	0	0	0	0	0	0	0
Viruses	Viruses_noname	Viruses_noname	Viruses_noname	Partitiviridae	NA	NA	NA	0	0	0	0	0	0	0	0	0	0	0.06346	0	0	0.00068	0	0.06992	0.00099	0
Bacteria	Actinobacteria	Actinobacteria	Solirubrobacterales	Patulibacteraceae	NA	NA	NA	0	0	0	0.01263	0	0	0	0	0	0	0	0	0	0	0	0	0	0
Bacteria	Firmicutes	Clostridia	Clostridiales	Peptostreptococcaceae	NA	NA	NA	0.31465	0.40003	88.20273	0.25685	4.25478	0	0.47515	0	0	0.14697	0.01878	96.77642	10.99065	0.22925	5.28887	0	0	0.05322
Bacteria	Proteobacteria	Alphaproteobacteria	Rhizobiales	Phyllobacteriaceae	NA	NA	NA	0	0	0	0	0	0	0	0.03333	0	0	0	0	0	0	0	0	0	0
Viruses	Viruses_noname	Viruses_noname	Picornavirales	Picornaviridae	NA	NA	NA	0	0	0	0	0	0	0	0	0	0	0	0	0	7.00E-05	0	0	0	0
Bacteria	Bacteroidetes	Bacteroidia	Bacteroidales	Porphyromonadaceae	NA	NA	NA	0.12069	0	0	0	0.62065	0	0	0	0	0.0013	0	0	0	0.0005	0	0	0	0
Bacteria	Actinobacteria	Actinobacteria	Actinomycetales	Propionibacteriaceae	NA	NA	NA	0	0	0	0.69416	0.00577	0	0	1.40214	0	0	0	0	14.85175	0	0.47646	0.09626	0.00027	0.001
Viruses	Viruses_noname	Viruses_noname	Viruses_noname	Retroviridae	NA	NA	NA	0	0.29704	0.31676	1.32363	0.22374	0.14266	0	0.23292	0.34492	0.00974	0.00055	0.1583	30.60361	0.00057	0	0.15868	0.00224	0
Bacteria	Bacteroidetes	Bacteroidia	Bacteroidales	Rikenellaceae	NA	NA	NA	2.33005	0.12233	0	0	6.56694	3.12911	0.13126	8.42416	5.54213	0	0	0	0	0.00021	0.60568	0	0.00021	0
Bacteria	Firmicutes	Clostridia	Clostridiales	Ruminococcaceae	NA	NA	NA	25.41495	12.50342	0	0	21.96005	27.4239	60.10952	27.07407	61.2125	0.0139	0.00356	0	0	0.00051	1.73089	0.0283	0.0001	0.0033
Bacteria	Actinobacteria	Actinobacteria	Actinomycetales	Sanguibacteraceae	NA	NA	NA	0	0	0	0.06648	0.0358	0	0	0	0	0	0	0	0	0	0	0	0	0
Viruses	Viruses_noname	Viruses_noname	Picornavirales	Secoviridae	NA	NA	NA	0	0	0	0	0	0	0	0	0	0	0	0	0	0.0073	0.94145	0	0.50147	0
Viruses	Viruses_noname	Viruses_noname	Caudovirales	Siphoviridae	NA	NA	NA	0	0	0	0	0	0	0	1.60394	1.96681	0	0	0	0	0	0	0	0	0
Bacteria	Actinobacteria	Actinobacteria	Solirubrobacterales	Solirubrobacterales_unclassified	NA	NA	NA	0	0.68482	0	4.58745	0.88599	0.14375	0	0.65495	0.06143	0	0	0	0	0	0	0	0	0
Bacteria	Proteobacteria	Alphaproteobacteria	Sphingomonadales	Sphingomonadaceae	NA	NA	NA	0	0	0	0	0	0	0	0	0	0	0.00149	0.09387	6.00394	0.00099	1.85942	0.0698	0.00085	0.00114
Bacteria	Firmicutes	Bacilli	Lactobacillales	Streptococcaceae	NA	NA	NA	0	0	0	0.09849	0	0	0	0	0	0	0	0	0	0	0	0	0	0
Bacteria	Proteobacteria	Betaproteobacteria	Burkholderiales	Sutterellaceae	NA	NA	NA	1.99192	0.34971	0	0	1.83764	0.70754	3.00672	1.04329	1.59449	0.07948	0.01018	0	0	0.00186	7.7962	0.62743	0.00131	0.00548
Bacteria	Deinococcus_Thermus	Deinococci	Thermales	Thermaceae	NA	NA	NA	0	0	0	0	0	0	0	0.12236	0	0	0	0	0	0	0	0	0	0

Viruses	Viruses_noname	Viruses_noname	Tymovirales	Tymoviridae	NA	NA	NA	0	0	0	0	0	0	0	0	0	0.23753	0.20474	0	0	0.00135	0	0	0	0.3249
Bacteria	Firmicutes	Negativicutes	Selenomonadales	Veillonellaceae	NA	NA	NA	7.70107	2.41656	0	0	0.36333	47.5784	9.62536	0.09417	4.98249	0.00144	5.00E-05	0	0	0	28.22766	0.05093	0	0.00206
Viruses	Viruses_noname	Viruses_noname	Viruses_noname	Virgaviridae	NA	NA	NA	0	0	0	0	0.05243	0	0.2459	0	0	0	0	0	0	0	14.2738	0.43941	0	0
Viruses	Viruses_noname	Viruses_noname	Viruses_noname	Viruses_noname	NA	NA	NA	0.72991	0.27839	0	0	0.99204	0	1.65336	0.84246	1.08685	0.63371	9.84088	0	0	0.01494	0	8.70636	97.55168	0.28104
Bacteria	Proteobacteria	Gammaproteobacteria	Xanthomonadales	Xanthomonadaceae	NA	NA	NA	0	0	0	0.14104	0	0	0	0	0	0	0	0	0	0	0	0	0	0
Bacteria	NA	NA	NA	NA	NA	NA	NA	97.44325	57.84395	98.02198	97.65112	97.33487	98.87286	94.99717	96.2664	93.54116	0.52424	0.04517	97.15815	33.50195	0.24126	75.71475	1.78784	0.00476	0.07722
Bacteria	Actinobacteria	NA	NA	NA	NA	NA	NA	2.02772	13.70277	0	77.17361	19.45662	7.58227	5.75252	56.17629	9.29476	0.01662	0.00095	0	14.85175	0.00105	4.93216	0.16245	0.00035	0.00334
Bacteria	Actinobacteria	Actinobacteria	NA	NA	NA	NA	NA	2.02772	13.70277	0	77.17361	19.45662	7.58227	5.75252	56.17629	9.29476	0.01662	0.00095	0	14.85175	0.00105	4.93216	0.16245	0.00035	0.00334
Bacteria	Actinobacteria	Actinobacteria	Actinomycetales	NA	NA	NA	NA	0.70716	11.9183	0	72.57354	16.69354	3.21164	1.76008	54.35773	3.76136	0	0	0	14.85175	9.00E-05	0.47646	0.09626	0.00032	0.001
Bacteria	Actinobacteria	Actinobacteria	Bifidobacteriales	NA	NA	NA	NA	0	0	0	0	0	0	0.13085	0	0	0	0	0	0	0	0	0	0	0
Bacteria	Actinobacteria	Actinobacteria	Coriobacteriales	NA	NA	NA	NA	1.32056	1.09966	0	0	1.87708	4.22688	3.86159	1.16361	5.47197	0.01662	0.00095	0	0	0.00096	4.4557	0.06619	2.00E-05	0.00234
Bacteria	Actinobacteria	Actinobacteria	Solirubrobacterales	NA	NA	NA	NA	0	0.68482	0	4.60008	0.88599	0.14375	0	0.65495	0.06143	0	0	0	0	0	0	0	0	0
Bacteria	Bacteroidetes	NA	NA	NA	NA	NA	NA	3.90665	0.30231	0	0	7.34637	3.77744	3.09091	8.50167	5.86998	0.0013	0	0	0	0.00071	0.61268	0	0.00021	0
Bacteria	Bacteroidetes	Bacteroidia	NA	NA	NA	NA	NA	3.90665	0.30231	0	0	7.34637	3.77744	3.09091	8.50167	5.86998	0.0013	0	0	0	0.00071	0.61268	0	0.00021	0
Bacteria	Bacteroidetes	Bacteroidia	Bacteroidales	NA	NA	NA	NA	3.90665	0.30231	0	0	7.34637	3.77744	3.09091	8.50167	5.86998	0.0013	0	0	0	0.00071	0.61268	0	0.00021	0
Bacteria	Cyanobacteria	NA	NA	NA	NA	NA	NA	0	0	0.34458	0	0	0	0	0	0	0	0	0	0	0	0	0	0	0
Bacteria	Cyanobacteria	Cyanobacteria_noname	NA	NA	NA	NA	NA	0	0	0.34458	0	0	0	0	0	0	0	0	0	0	0	0	0	0	0
Bacteria	Cyanobacteria	Cyanobacteria_noname	Chroococcales	NA	NA	NA	NA	0	0	0.34458	0	0	0	0	0	0	0	0	0	0	0	0	0	0	0
Bacteria	Deferribacteres	NA	NA	NA	NA	NA	NA	32.74782	0.86187	0	0	0.78266	0.04654	0	0	0.92847	0.06171	0.00011	0	0	8.00E-05	0.03564	0	0	0.00048
Bacteria	Deferribacteres	Deferribacteres	NA	NA	NA	NA	NA	32.74782	0.86187	0	0	0.78266	0.04654	0	0	0.92847	0.06171	0.00011	0	0	8.00E-05	0.03564	0	0	0.00048
Bacteria	Deferribacteres	Deferribacteres	Deferribacterales	NA	NA	NA	NA	32.74782	0.86187	0	0	0.78266	0.04654	0	0	0.92847	0.06171	0.00011	0	0	8.00E-05	0.03564	0	0	0.00048
Bacteria	Deinococcus_Thermus	NA	NA	NA	NA	NA	NA	0	0	0	0	0	0	0	0.12236	0	0	0	0	0	0	0	0	0	0
Bacteria	Deinococcus_Thermus	Deinococci	NA	NA	NA	NA	NA	0	0	0	0	0	0	0	0.12236	0	0	0	0	0	0	0	0	0	0
Bacteria	Deinococcus_Thermus	Deinococci	Thermales	NA	NA	NA	NA	0	0	0	0	0	0	0	0.12236	0	0	0	0	0	0	0	0	0	0
Bacteria	Firmicutes	NA	NA	NA	NA	NA	NA	39.1449	18.14118	97.60236	3.71808	38.14196	82.37795	83.14701	30.09612	74.42587	0.34829	0.02955	97.06429	10.99065	0.23485	55.69019	0.92816	0.00205	0.06631
Bacteria	Firmicutes	Bacilli	NA	NA	NA	NA	NA	0	0.48471	0	3.43523	7.63838	1.14519	0	0.00542	0.15661	0	0	0	0	0.00015	0	0	0	0
Bacteria	Firmicutes	Bacilli	Bacillales	NA	NA	NA	NA	0	0.06514	0	0	0	0	0	0.00542	0	0	0	0	0	0	0	0	0	0
Bacteria	Firmicutes	Bacilli	Lactobacillales	NA	NA	NA	NA	0	0.41956	0	3.43523	7.63838	1.14519	0	0	0.15661	0	0	0	0	0.00015	0	0	0	0
Bacteria	Firmicutes	Clostridia	NA	NA	NA	NA	NA	31.44382	15.23991	88.78409	0.25685	30.14024	33.65436	73.52166	29.99653	69.28677	0.34684	0.0295	96.77865	10.99065	0.23469	27.46253	0.87722	0.00205	0.06426
Bacteria	Firmicutes	Clostridia	Clostridiales	NA	NA	NA	NA	31.44382	15.23991	88.78409	0.25685	30.14024	33.65436	73.52166	29.99653	69.28677	0.34684	0.0295	96.77865	10.99065	0.23469	27.46253	0.87722	0.00205	0.06426
Bacteria	Firmicutes	Erysipelotrichia	NA	NA	NA	NA	NA	0	0	8.81827	0.026	0	0	0	0	0	0	0	0.28563	0	0	0	0	0	0
Bacteria	Firmicutes	Erysipelotrichia	Erysipelotrichales	NA	NA	NA	NA	0	0	8.81827	0.026	0	0	0	0	0	0	0	0.28563	0	0	0	0	0	0
Bacteria	Firmicutes	Negativicutes	NA	NA	NA	NA	NA	7.70107	2.41656	0	0	0.36333	47.5784	9.62536	0.09417	4.98249	0.00144	5.00E-05	0	0	0	28.22766	0.05093	0	0.00206
Bacteria	Firmicutes	Negativicutes	Selenomonadales	NA	NA	NA	NA	7.70107	2.41656	0	0	0.36333	47.5784	9.62536	0.09417	4.98249	0.00144	5.00E-05	0	0	0	28.22766	0.05093	0	0.00206
Bacteria	Proteobacteria	NA	NA	NA	NA	NA	NA	19.61615	24.83581	0	16.75943	31.60727	3.03519	3.00672	1.36995	3.02209	0.09632	0.01456	0.09387	7.65955	0.00457	9.65563	0.69723	0.00216	0.00709
Bacteria	Proteobacteria	Alphaproteobacteria	NA	NA	NA	NA	NA	0	0	0	0	0.13437	0	0	0.1545	0	0	0.00149	0.09387	6.00394	0.00099	1.85942	0.0698	0.00085	0.00114
Bacteria	Proteobacteria	Alphaproteobacteria	Rhizobiales	NA	NA	NA	NA	0	0	0	0	0.13437	0	0	0.1545	0	0	0	0	0	0	0	0	0	0
Bacteria	Proteobacteria	Alphaproteobacteria	Sphingomonadales	NA	NA	NA	NA	0	0	0	0	0	0	0	0	0	0	0.00149	0.09387	6.00394	0.00099	1.85942	0.0698	0.00085	0.00114
Bacteria	Proteobacteria	Betaproteobacteria	NA	NA	NA	NA	NA	1.99192	0.34971	0	0	1.83764	0.70754	3.00672	1.15137	1.59449	0.07948	0.01018	0	1.6556	0.00186	7.7962	0.62743	0.00131	0.00548
Bacteria	Proteobacteria	Betaproteobacteria	Burkholderiales	NA	NA	NA	NA	1.99192	0.34971	0	0	1.83764	0.70754	3.00672	1.15137	1.59449	0.07948	0.01018	0	1.6556	0.00186	7.7962	0.62743	0.00131	0.00548
Bacteria	Proteobacteria	Deltaproteobacteria	NA	NA	NA	NA	NA	0	0	0	0	0.09051	0.16372	0	0	0.1324	0	0	0	0	3.00E-05	0	0	0	0
Bacteria	Proteobacteria	Deltaproteobacteria	Desulfovibrionales	NA	NA	NA	NA	0	0	0	0	0.09051	0.16372	0	0	0.1324	0	0	0	0	3.00E-05	0	0	0	0
Bacteria	Proteobacteria	Epsilonproteobacteria	NA	NA	NA	NA	NA	17.52089	24.45982	0	0	2.12769	0	0	0	1.14132	0.01684	0.00288	0	0	0.00027	0	0	0	0.00046
Bacteria	Proteobacteria	Epsilonproteobacteria	Campylobacterales	NA	NA	NA	NA	17.52089	24.45982	0	0	2.12769	0	0	0	1.14132	0.01684	0.00288	0	0	0.00027	0	0	0	0.00046
Bacteria	Proteobacteria	Gammaproteobacteria	NA	NA	NA	NA	NA	0.10334	0.02628	0	16.75943	27.41706	2.16393	0	0.06409	0.15388	0	0	0	0	0.00143	0	0	0	0
Bacteria	Proteobacteria	Gammaproteobacteria	Enterobacteriales	NA	NA	NA	NA	0.10334	0.02628	0	16.61839	27.41706	2.16393	0	0.06409	0.15388	0	0	0	0	0.00143	0	0	0	0
Bacteria	Proteobacteria	Gammaproteobacteria	Xanthomonadales	NA	NA	NA	NA	0	0	0	0.14104	0	0	0	0	0	0	0	0	0	0	0	0	0	0
Bacteria	Spirochaetes	NA	NA	NA	NA	NA	NA	0	0	0	0	0	2.05348	0	0	0	0	0	0	0	0	4.78845	0	0	0
Bacteria	Spirochaetes	Spirochaetia	NA	NA	NA	NA	NA	0	0	0	0	0	2.05348	0	0	0	0	0	0	0	0	4.78845	0	0	0
Bacteria	Spirochaetes	Spirochaetia	Spirochaetales	NA	NA	NA	NA	0	0	0	0	0	2.05348	0	0	0	0	0	0	0	0	4.78845	0	0	0
Bacteria	Tenericutes	NA	NA	NA	NA	NA	NA	0	0	0.07504	0	0	0	0	0	0	0	0	0	0	0	0	0	0	0
Bacteria	Tenericutes	Mollicutes	NA	NA	NA	NA	NA	0	0	0.07504	0	0	0	0	0	0	0	0	0	0	0	0	0	0	0
Bacteria	Tenericutes	Mollicutes	Mycoplasmatales	NA	NA	NA	NA	0	0	0.07504	0	0	0	0	0	0	0	0	0	0	0	0	0	0	0
Eukaryota	NA	NA	NA	NA	NA	NA	NA	0	0.3076	0	0	0	0	0	0	0	0	0	0	0	0	0	0	0	0
Eukaryota	Microsporidia	NA	NA	NA	NA	NA	NA	0	0.3076	0	0	0	0	0	0	0	0	0	0	0	0	0	0	0	0
Eukaryota	Microsporidia	Microsporidia_noname	NA	NA	NA	NA	NA	0	0.3076	0	0	0	0	0	0	0	0	0	0	0	0	0	0	0	0
Eukaryota	Microsporidia	Microsporidia_noname	Microsporidia_noname	NA	NA	NA	NA	0	0.3076	0	0	0	0	0	0	0	0	0	0	0	0	0	0	0	0
Viruses	NA	NA	NA	NA	NA	NA	NA	2.55675	41.84846	1.97802	2.34888	2.66513	1.12714	5.00283	3.7336	6.45884	99.47576	99.95483	2.84185	66.49805	99.75874	24.28525	98.21216	99.99524	99.92278
Viruses	Viruses_noname	NA	NA	NA	NA	NA	NA	2.55675	41.84846	1.97802	2.34888	2.66513	1.12714	5.00283	3.7336	6.45884	99.47576	99.95483	2.84185	66.49805	99.75874	24.28525	98.21216	99.99524	99.92278
Viruses	Viruses_noname	Viruses_noname	NA	NA	NA	NA	NA	2.55675	41.84846	1.97802	2.34888	2.66513	1.12714	5.00283	3.7336	6.45884	99.47576	99.95483	2.84185	66.49805	99.75874	24.28525	98.21216	99.99524	99.92278
Viruses	Viruses_noname	Viruses_noname	Caudovirales	NA	NA	NA	NA	0	0	0	0	0	0	0	1.60394	1.96681	0	0	0	0	0	0	0	0	0
Viruses	Viruses_noname	Viruses_noname	Picornavirales	NA	NA	NA	NA	0	0	0	0	0	0	0	0	0	0	0	0	0	0.00736	0.94145	0	0.50147	0
Viruses	Viruses_noname	Viruses_noname	Tymovirales	NA	NA	NA	NA	0	0	0.41221	0	0	0	0	0	0	18.23978	60.32412	0	0	1.08265	0	35.70817	1.8846	89.74571
Viruses	Viruses_noname	Viruses_noname	Viruses_noname	NA	NA	NA	NA	2.55675	41.84846	1.5658	2.34888	2.66513	1.12714	5.00283	2.12966	4.49203	81.23598	39.63071	2.84185	66.49805	98.66872	23.3438	62.50399	97.60917	10.17707

Supplemental Table 8: Metadata for the samples.

ID	data_type	designation	band_number	sex	age	location	date	buffer	County	State	CleanedZymo	Kit	sample	latitude	longitude	EC_counts	KO_counts	Paths_counts	Paths_Pro	EC_Pro	KO_Pro	CAZy_ECs
Z1	simulatedMG	Sample 1	120855854	F	HY	Stewart Park	26-Jun	Y	Tompkins	NY	No	Zymo	F_HY_NY	42.4614	-76.5032	1710	5141	339	339	1710	5141	52
Z2	simulatedMG	Sample 2	120848766	F	HY	Walker Reservoir	25-Jun	Y	Tolland	CT	Yes	Zymo	F_HY_CT	41.8491	-72.4308	1760	5478	353	353	1760	5478	55
Z3	simulatedMG	Sample 3	120848474	M	HY	Enfield	21-Jun	Y	Hartford	CT	Yes	Zymo	M_HY_CT	42.0027	-72.5441	1799	5591	357	357	1799	5591	53
Z5	simulatedMG	Sample 5	120848765	F	HY	Walker Reservoir	25-Jun	Y	Tolland	CT	Yes	Zymo	F_HY_CT	41.8491	-72.4308	1535	4780	311	311	1535	4780	58
Z6	simulatedMG	Sample 6	120848501	M	adult	Windsor	21-Jun	Y	Hartford	CT	No	Zymo	M_adult_CT	41.8526	-72.6437	1582	4921	324	324	1582	4921	48
Z7	simulatedMG	Sample 7	120848658	F	adult	Essex	22-Jun	Y	Essex	CT	No	Zymo	F_adult_CT	41.3504	-72.4052	1859	5659	366	366	1859	5659	54
Z9	simulatedMG	Sample 9	120855856	F	HY	Stewart Park	26-Jun	Y	Tompkins	NY	No	Zymo	F_HY_NY	42.4614	-76.5032	1806	5720	355	355	1806	5720	50
Z10	simulatedMG	Sample 10	120855974	M	HY	Cass Park	26-Jun	Y	Tompkins	NY	No	Zymo	M_HY_NY	42.4526	-76.5144	1707	5086	341	341	1707	5086	53
Z11	simulatedMG	Sample 11	120848638	F	adult	Essex	22-Jun	Y	Essex	CT	No	Zymo	F_adult_CT	41.3504	-72.4052	1436	4205	293	293	1436	4205	43
ZD1	MG	Sample 1	120855854	F	HY	Stewart Park	26-Jun	Y	Tompkins	NY	No	Zymo	F_HY_NY	42.4614	-76.5032	1666	4443	371	324	1546	3549	68
ZD2	MG	Sample 2	120848766	F	HY	Walker Reservoir	25-Jun	Y	Tolland	CT	Yes	Zymo	F_HY_CT	41.8491	-72.4308	1993	5739	468	399	1828	4631	61
ZD3	MG	Sample 3	120848474	M	HY	Enfield	21-Jun	Y	Hartford	CT	Yes	Zymo	M_HY_CT	42.0027	-72.5441	2404	6851	529	450	2195	5434	80
ZD5	MG	Sample 5	120848765	F	HY	Walker Reservoir	25-Jun	Y	Tolland	CT	Yes	Zymo	F_HY_CT	41.8491	-72.4308	1743	4591	404	355	1654	4299	71
ZD6	MG	Sample 6	120848501	M	adult	Windsor	21-Jun	Y	Hartford	CT	No	Zymo	M_adult_CT	41.8526	-72.6437	1732	3925	387	337	1637	3603	70
ZD7	MG	Sample 7	120848658	F	adult	Essex	22-Jun	Y	Essex	CT	No	Zymo	F_adult_CT	41.3504	-72.4052	2621	7871	559	452	2198	5136	90
ZD9	MG	Sample 9	120855856	F	HY	Stewart Park	26-Jun	Y	Tompkins	NY	No	Zymo	F_HY_NY	42.4614	-76.5032	1827	4990	421	366	1698	3869	69
ZD10	MG	Sample 10	120855974	M	HY	Cass Park	26-Jun	Y	Tompkins	NY	No	Zymo	M_HY_NY	42.4526	-76.5144	2108	5776	484	415	1957	4430	76
ZD11	MG	Sample 11	120848638	F	adult	Essex	22-Jun	Y	Essex	CT	No	Zymo	F_adult_CT	41.3504	-72.4052	1559	4112	375	323	1433	3250	51
ZR1	MT	Sample 1	120855854	F	HY	Stewart Park	26-Jun	Y	Tompkins	NY	No	Zymo	F_HY_NY	42.4614	-76.5032	1042	2828	268	241	907	1920	29
ZR2	MT	Sample 2	120848766	F	HY	Walker Reservoir	25-Jun	Y	Tolland	CT	Yes	Zymo	F_HY_CT	41.8491	-72.4308	737	2017	147	145	589	1282	13
ZR3	MT	Sample 3	120848474	M	HY	Enfield	21-Jun	Y	Hartford	CT	Yes	Zymo	M_HY_CT	42.0027	-72.5441	1568	4554	376	319	1294	3137	48
ZR5	MT	Sample 5	120848765	F	HY	Walker Reservoir	25-Jun	Y	Tolland	CT	Yes	Zymo	F_HY_CT	41.8491	-72.4308	1271	3416	299	265	1116	2594	38
ZR6	MT	Sample 6	120848501	M	adult	Windsor	21-Jun	Y	Hartford	CT	No	Zymo	M_adult_CT	41.8526	-72.6437	1129	2851	266	240	909	1896	35
ZR7	MT	Sample 7	120848658	F	adult	Essex	22-Jun	Y	Essex	CT	No	Zymo	F_adult_CT	41.3504	-72.4052	1143	2921	291	232	761	1475	28
ZR9	MT	Sample 9	120855856	F	HY	Stewart Park	26-Jun	Y	Tompkins	NY	No	Zymo	F_HY_NY	42.4614	-76.5032	1055	2919	245	222	906	1844	30
ZR10	MT	Sample 10	120855974	M	HY	Cass Park	26-Jun	Y	Tompkins	NY	No	Zymo	M_HY_NY	42.4526	-76.5144	1002	2472	251	227	867	1684	29
ZR11	MT	Sample 11	120848638	F	adult	Essex	22-Jun	Y	Essex	CT	No	Zymo	F_adult_CT	41.3504	-72.4052	1489	5747	339	271	1068	2442	39
